# Innate Immunity and the Inter-exposure Interval Determine the Dynamics of Secondary Influenza Virus Infection and Explain Observed Viral Hierarchies

**DOI:** 10.1371/journal.pcbi.1004334

**Published:** 2015-08-18

**Authors:** Pengxing Cao, Ada W. C. Yan, Jane M. Heffernan, Stephen Petrie, Robert G. Moss, Louise A. Carolan, Teagan A. Guarnaccia, Anne Kelso, Ian G. Barr, Jodie McVernon, Karen L. Laurie, James M. McCaw

**Affiliations:** 1 Centre for Epidemiology and Biostatistics, Melbourne School of Population and Global Health, The University of Melbourne, Melbourne, Australia; 2 Modelling Infection and Immunity Lab, Centre for Disease Modelling, York Institute for Health Research, York University, Toronto, Ontario, Canada; 3 Mathematics and Statistics, York University, Toronto, Ontario, Canada; 4 WHO Collaborating Centre for Reference and Research on Influenza at the Peter Doherty Institute for Infection and Immunity, Parkville, Victoria, Australia; 5 Modelling and Simulation, Infection and Immunity Theme, Murdoch Childrens Research Institute, The Royal Children’s Hospital, Parkville, Victoria, Australia; 6 School of Mathematics and Statistics, The University of Melbourne, Melbourne, Australia; Duke University, UNITED STATES

## Abstract

Influenza is an infectious disease that primarily attacks the respiratory system. Innate immunity provides both a very early defense to influenza virus invasion and an effective control of viral growth. Previous modelling studies of virus–innate immune response interactions have focused on infection with a single virus and, while improving our understanding of viral and immune dynamics, have been unable to effectively evaluate the relative feasibility of different hypothesised mechanisms of antiviral immunity. In recent experiments, we have applied consecutive exposures to different virus strains in a ferret model, and demonstrated that viruses differed in their ability to induce a state of temporary immunity or viral interference capable of modifying the infection kinetics of the subsequent exposure. These results imply that virus-induced early immune responses may be responsible for the observed viral hierarchy. Here we introduce and analyse a family of within-host models of re-infection viral kinetics which allow for different viruses to stimulate the innate immune response to different degrees. The proposed models differ in their hypothesised mechanisms of action of the non-specific innate immune response. We compare these alternative models in terms of their abilities to reproduce the re-exposure data. Our results show that 1) a model with viral control mediated solely by a virus-resistant state, as commonly considered in the literature, is not able to reproduce the observed viral hierarchy; 2) the synchronised and desynchronised behaviour of consecutive virus infections is highly dependent upon the interval between primary virus and challenge virus exposures and is consistent with virus-dependent stimulation of the innate immune response. Our study provides the first mechanistic explanation for the recently observed influenza viral hierarchies and demonstrates the importance of understanding the host response to multi-strain viral infections. Re-exposure experiments provide a new paradigm in which to study the immune response to influenza and its role in viral control.

## Author Summary

Infection with the influenza virus is responsible for serious morbidity and mortality. In otherwise-healthy individuals, infection is usually acute, lasting between 5 to 10 days. Over this time, the virus initially replicates rapidly, before peaking and then being cleared from the body. Despite extensive study, we do not yet fully understand the processes which lead to viral control and viral clearance from the host. Experimental and modelling studies of single infections have previously indicated that both the innate and adaptive immune responses play an important role in combating infection. Here we study a novel dataset on how the host responds to sequential exposure to two different strains of influenza. We introduce a family of mathematical models of the within-host dynamics of influenza infection which allow for re-infection. Our models allow us, for the first time, to differentiate between alternative hypothesised mechanisms by which the innate immune response acts to control viral replication. This study improves our understanding of the innate immune response to influenza and demonstrates that re-exposure studies provide a new paradigm for further experimental research. Our findings may contribute to the development of next-generation treatment and vaccination strategies which rely upon an understanding of the host’s immunological response to influenza infection.

## Introduction

Influenza is an infectious respiratory disease affecting and threatening millions of people worldwide [1]. The invasion of the influenza virus into a host’s upper respiratory tract (URT) starts from a sufficient number of virions (single viral particles) entering the URT and infecting healthy epithelial cells (henceforth referred to as target cells) [2]. The infected cells then produce progeny virions, leading to further infection of target cells and inter-host transmission.

Immune responses are activated during influenza virus infection, and contribute to the control of infection and viral clearance from the host [3]. The innate immune response, initiated in the early stage of infection, involves production of a variety of antiviral cytokines, which provide immediate non-specific protection to the target cells against infection [4, 5]. Of particular importance is the cytokine interferon (IFN, type 1), whose protective functions include inducing a virus-resistant state in target cells, reducing viral replication, and activating natural killer (NK) cells to induce apoptosis in infected cells [6–9]. The adaptive immune response, once stimulated by presentation of viral epitopes to lymphocytes, plays an important role in viral control. B lymphocytes (or B cells) produce antibodies that neutralise free virus, and cytotoxic T lymphocytes (or T cells) produce cytotoxic granules that kill infected epithelial cells and other leukocytes [3]. Following viral clearance, a portion of those B cells and T cells become long-lived memory cells which can be activated rapidly to form a defense upon re-exposure to the same or an antigenically related virus.

**Fig 1:**
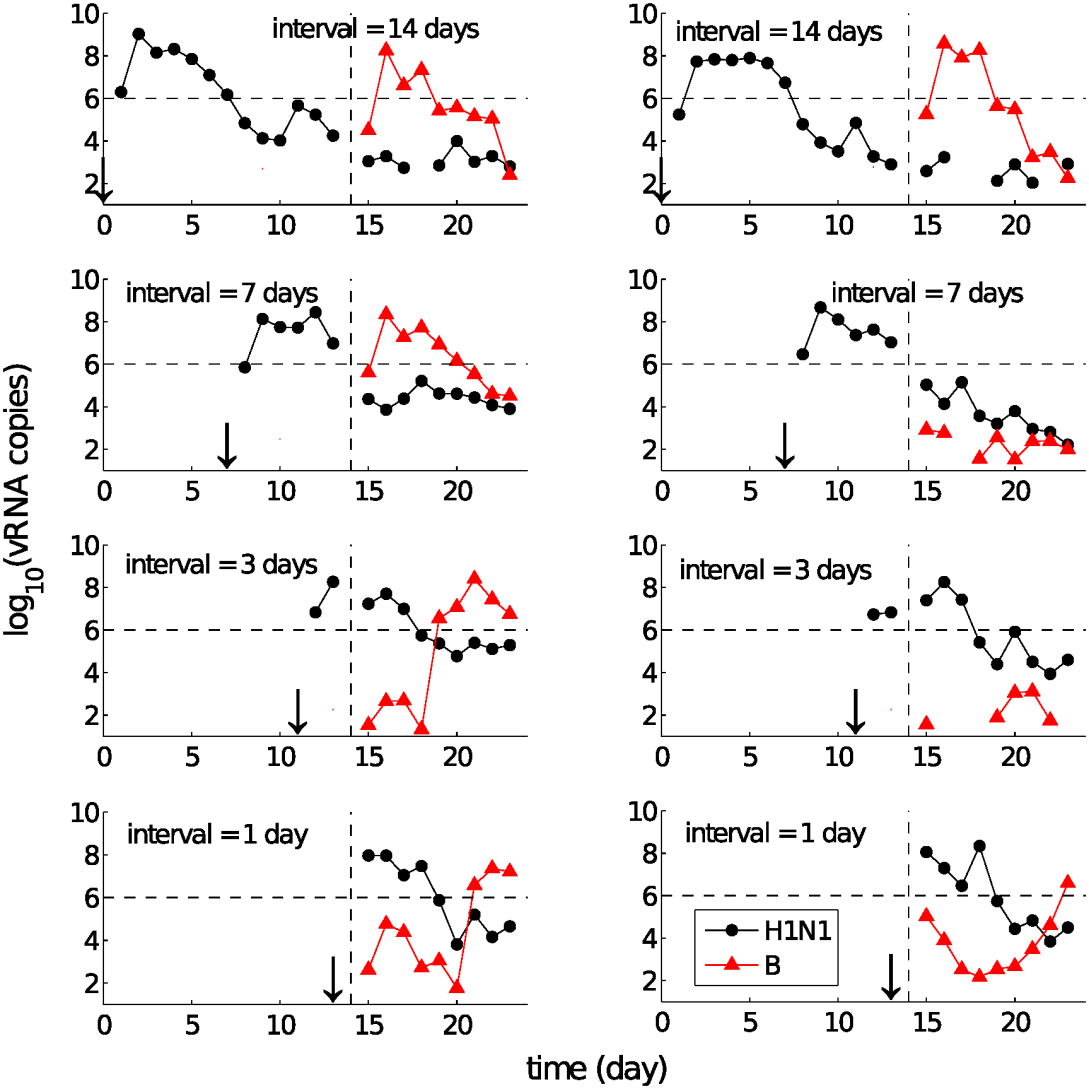
Re-exposure experimental data for primary exposure with A(H1N1)pdm09 (black dots) and challenge with influenza B virus (red triangles), for IEI between 1 and 14 days. Viral RNA copy number (per 100 *μl*) was measured to indicate the level of viral load in ferret nasal washes. By varying the IEI from 1 to 14 days as indicated in each subfigure we see that A(H1N1)pdm09 virus could block (i.e. the influenza B viral load was never above the defined threshold of 10^6^ vRNA copies indicated by the horizontal dashed lines) or delay the infection induced by influenza B virus for ≤ 7 days. The threshold of 10^6^ is the limit of detection for the TCID_50_ assay which measures infectious virus [10]. For a clearer view, data are presented here such that the time of challenge (exposure to the second virus) is fixed at day 14 (indicated by the dashed lines) whereas the primary exposure occurs an appropriate number of days earlier (indicated by arrows and the exposure interval numbers). Missing data points in the curves indicate undetectable levels of viral load and no sample was collected on the day of challenge. Each graph represents the data from a single ferret, so that two ferrets within each interval are shown here. Data used from [10].

**Fig 2:**
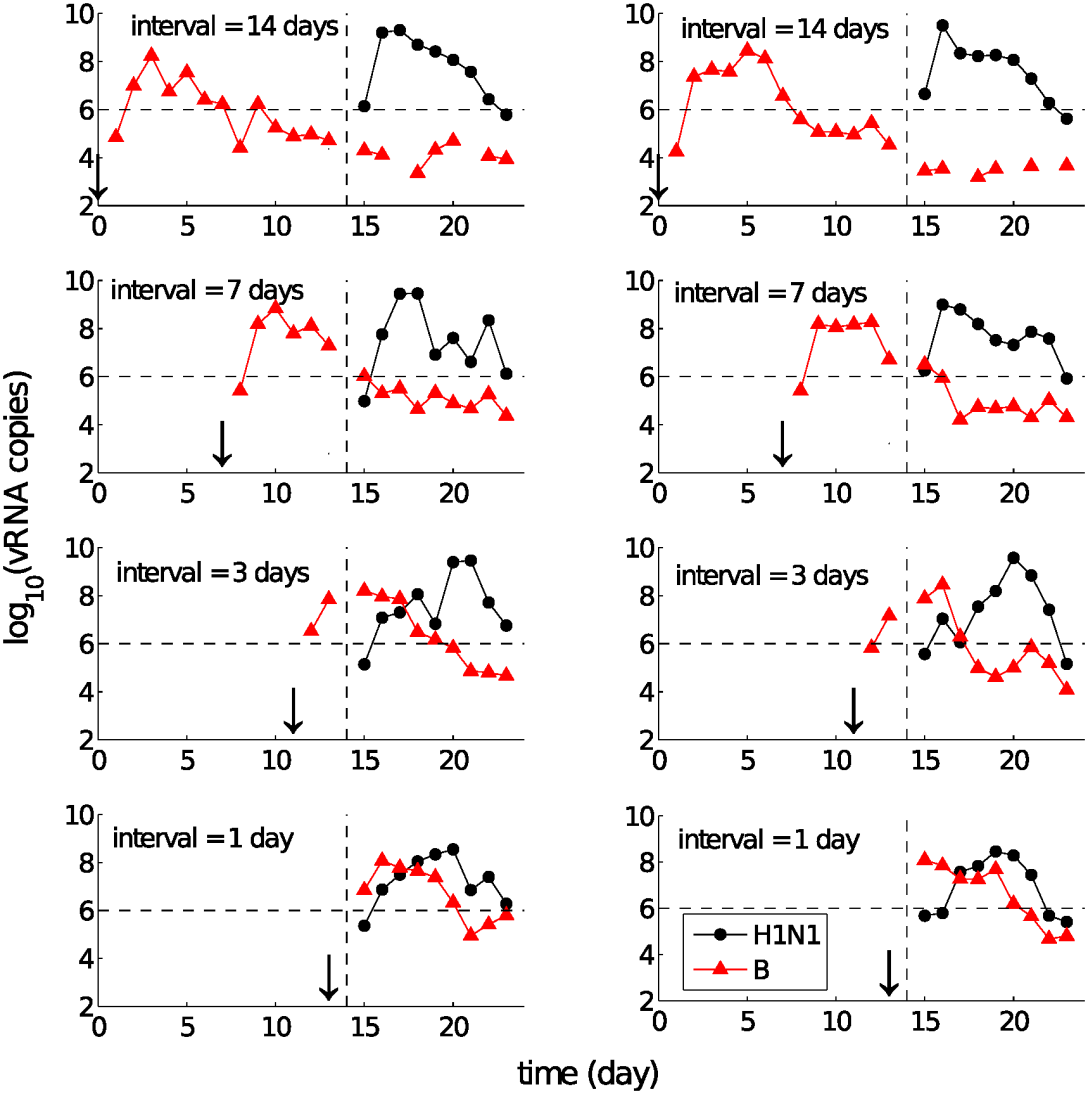
Re-exposure experimental data for primary exposure with influenza B virus followed by challenge with A(H1N1)pdm09. In contrast to Fig 1, changing the IEI does not block the infection of A(H1N1)pdm09 virus for any infection period. It does, however, result in a reduced growth rate and delayed time to peak virus titre for A(H1N1)pdm09 at short IEIs. Missing data points in the curves indicate undetectable levels of viral load and no sample was taken on the day of challenge. Each graph represents the data from a single ferret, so that two ferrets within each interval are shown here. All symbols are the same as those in Fig 1. Data used from [10].

Due to its non-specific nature, the innate response induced by an initial exposure (henceforth “primary infection”) would be expected to modify the host environment and provide some protection to subsequent exposure (henceforth “challenge”). We have experimentally studied this phenomenon in detail by examining the behaviour of consecutive influenza infections as a function of the delay between exposures [10]. By varying influenza virus types and subtypes (three viruses, A(H1N1)pdm09, A(H3N2) and influenza B, were investigated) and the delay between the exposures (henceforth the “inter-exposure interval” (IEI)), we found that a state of temporary immunity induced by A(H1N1)pdm09 was able to block or delay infection with influenza B virus (Fig 1). Conversely, influenza B virus showed little or no inhibitory effect on subsequent infection with A(H1N1)pdm09 (Fig 2). See [10] for all available data and a full exposition of the experimental observations.

**Fig 3:**
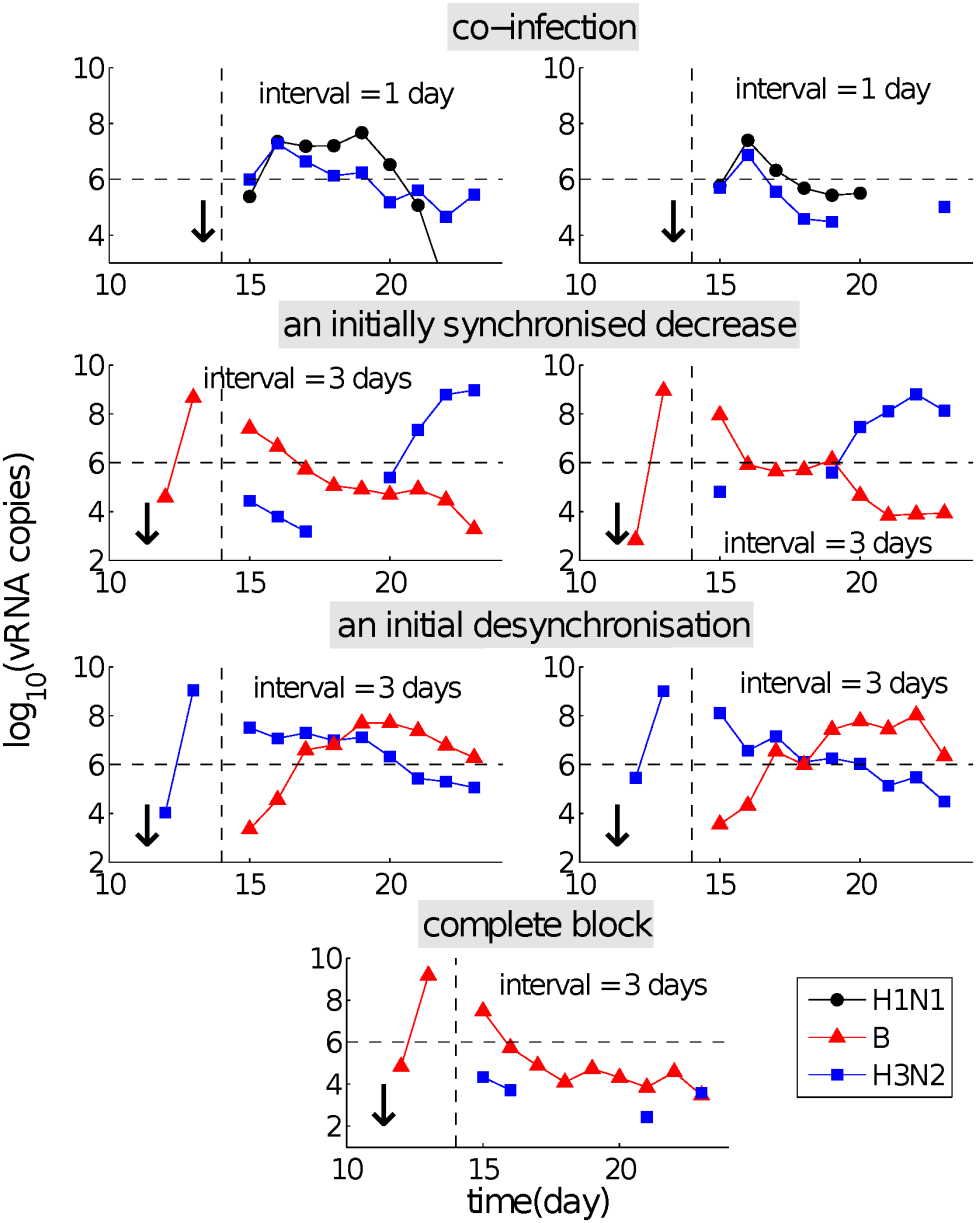
Re-exposure experimental data showing the four dominant patterns observed in the viral kinetics for primary–challenge virus pairs. The data shown here are distinct from those shown in Figs 1 and 2, where the same phenomena may also be observed, and a subset of the full data presented in [10]. Top panels show the case of co-infection, whereby both the primary (H1N1) and challenge (H3N2) viruses experience a synchronised increase in the very early stage of infection, followed by a synchronised decrease. Panels in the second row show examples of delayed infection, in which an initial synchronised decrease gives way to growth and successful infection with the challenge virus. The undetectable points between days 15 and 19 for the challenge virus in the right figure (second row) show a rapid decrease to undetectable viral level followed by a rapid upstroke back to a detectable level. Desynchronised viral kinetics in the early stage of infection are also observed for short IEIs, with examples shown in the third row of panels. The last well-observed pattern is that of a complete block, whereby the challenge virus is unable to replicate to a productive infection level (bottom panel). All symbols are the same as those in Fig 1. Data used from [10].

Although such experimental studies have improved our understanding of temporary immunity and viral interference, the underlying mechanisms of how a virus is controlled and cleared by the immune system are still not fully understood. In particular, the re-exposure experimental data revealed a number of novel properties and phenomena:

- Viruses differed in their ability to induce a state of temporary immunity or viral interference capable of modifying the infection kinetics of the subsequent exposure. For example, following primary infection with A(H1N1)pdm09 virus, subsequent challenge with influenza B virus was strongly inhibited (Fig 1), whereas the latter showed a very limited ability to inhibit the former (Fig 2; only weak delays for an IEI of 1–3 days are observed). These data suggest the existence of a “viral hierarchy” [10]. What are the mechanisms accounting for the interactions between the two different viruses and the induced hierarchy for different primary–challenge virus combinations?
- By looking at the details of viral kinetic time series, four types of patterns were identified (see Fig 3), which suggest some dynamical interactions between the two viruses. For example, an initial period of synchronised viral growth was often observed for short exposure intervals (≤ 3 days, see Figs 1–3). In addition to this, an initially synchronised decrease of the primary and challenge viruses was also frequently observed (Fig 3). What are the underlying mechanisms accounting for these phenomena?

Virus dynamics modelling has been employed to great success to gain insight into the host-pathogen interaction. For HIV in particular, mathematical models have proven invaluable in uncovering the mechanisms of immunity, anti-retroviral drug action and developing strategies to avoid or combat drug-resistance [11–15]. For influenza, due largely to a paucity of data and the difficulty in working with a short-lived transient infection, models have traditionally had less of an impact on our understanding of the immune response to influenza and the mechanism of viral control. In recent years however, both qualitative and quantitative modelling studies [16–33] have begun to probe these interactions more deeply, as recently reviewed by Beauchemin et al. [34] and Dobrovolny et al. [35]. While some studies have focused on the role of antiviral drugs in viral control (e.g. [30]) and others on the immune response, the majority have considered only a single viral infection. Exceptions include the development of models of multi-strain infection that have been used to study the within-host emergence of drug-resistant [20] and pandemic influenza [33] viruses and the relative fitness of drug-resistant variants [31, 32]. Here, with our focus on the immune response, it is immediately obvious that the classic Target cell–Infected cell–Virus (TIV) model, with control solely mediated by target-cell depletion and with no allowance for cell re-growth, is unable to explain re-infection with a different challenge virus. This provides the motivation to study experiments in which re-infection occurs as a means to explore the role of the immune response in influenza viral dynamics. Our data affords us the opportunity to examine the relative importance and feasibility of different hypothesised mechanisms of the innate immune response, complementing the work of others who have studied how immunity may influence viral kinetics using data from single viral infections [17, 18, 23, 24, 28].

In this paper we introduce and analyse a family of within-host models of re-infection viral kinetics which allow for different viruses to stimulate the innate immune response to different degrees. The proposed models differ in their hypothesised mechanisms of the non-specific innate immune response. We evaluate the models’ capability in terms of their ability to reproduce the patterns observed in the re-exposure data, including co-infection with and suppression, delay or blocking of the challenge virus (Figs 1–3). Our analyses demonstrate that the occurrence of those phenomena is highly dependent upon the inter-exposure interval and consistent with virus-dependent stimulation of the innate immune response. Our paper provides the first mechanistic explanation for the recently observed influenza viral hierarchies.

## Materials and Methods

In this section, we first introduce a single virus model with different mechanisms utilised by innate immunity to control viral infection, and then extend the model to allow for consecutive exposures to two virus strains (henceforth the re-exposure model). Due to the well-established importance of IFN in mediating the innate immune response, this study focuses on modelling IFN-induced antiviral functions. However, the conceptual formulation of IFN-mediated innate immune dynamics is broadly applicable and suitable for describing similar non-specific immune processes, therefore not limiting the generality of the model results.

### Three mechanisms for the IFN-induced control of viral infection

**Fig 4:**
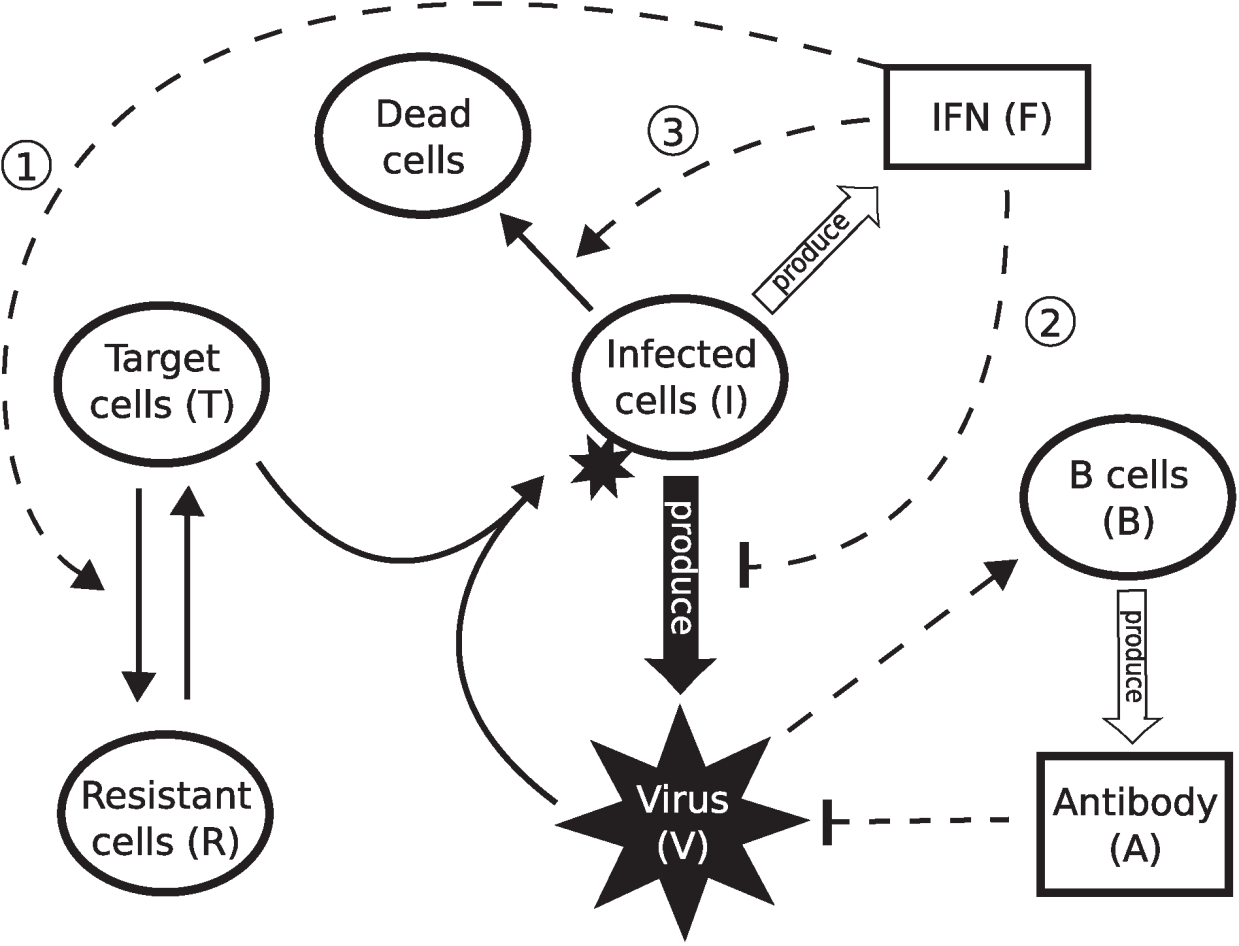
Schematic diagram showing the three major functions of IFN. The three major functions of IFN labelled by ①, ② and ③ correspond to Models 1–3 defined in the main text. Dashed curves with arrows indicate up-regulation while those with bars indicate down-regulation. Binding of virus to target cells leads to infected cells which then produce new virions. Infected cells produce IFN which hinders viral infection via three different hypothesised mechanisms: Model ① converting target cells to virus-resistant state; Model ② decreasing the viral production rate; and Model ③ inducing killing of infected cells by activation of NK cells. Virus activates B cells which produce antibodies to inactivate free virus.

Three possible antiviral mechanisms of IFN are allowed for in our model: 1) induction of a virus-resistant state for target cells; 2) a reduction in the viral production rate from infected cells; and 3) activation of NK cells to induce apoptosis in infected cells (Fig 4). With the additional inclusion of a strain-specific antibody response, the following equations describe the single virus-strain system:

$$\begin{array}{c} \frac{dV}{dt}=\frac{pI}{1+sF}-cV-\mu VA-\beta VT, \end{array}$$ (1)

$$\begin{array}{c} \frac{dT}{dt}=g\left(T+R\right)\left(1-\frac{T+R+I}{C_{t}}\right)-\beta^{\prime }VT+\rho R-\phi FT, \end{array}$$ (2)

$$\begin{array}{c} \frac{dI}{dt}=\beta^{\prime }VT-\delta I-\kappa IF, \end{array}$$ (3)

$$\begin{array}{c} \frac{dR}{dt}=\phi FT-\rho R-\xi R, \end{array}$$ (4)

$$\begin{array}{c} \frac{dF}{dt}=qI-dF, \end{array}$$ (5)

$$\begin{array}{c} \frac{dB}{dt}=m_{1}V\left(1-B\right)-m_{2}B, \end{array}$$ (6)

$$\begin{array}{c} \frac{dA}{dt}=m_{3}B-rA-\mu^{\prime }VA. \end{array}$$ (7)

The change in viral load (*dV*/*dt*) includes four components, the production term (*pI*/(1+*sF*)) in which virions are produced by infected cells (*I*) at a rate *p* subject to an IFN-dependent scaling factor of (1/(1+*sF*)) [17, 23, 28], the viral natural decay/clearance (*cV*) with a decay rate of *c*, the neutralisation term (*μVA*) by antibody (*A*), and a consumption term (*βVT*) due to binding to and infection of target cells (*T*). *s* indicates the sensitivity of the production rate to IFN. The term *g*(*T* + *R*)(1 − (*T* + *R* + *I*)/*C*_*t*_) models target cell (re-)growth by both target cells and resistant cells (those protected by the IFN) but limited by a maximum cell number *C*_*t*_ (e.g. due to the spatial capacity, [18]). Target cells (*T*) are consumed by virus (*V*) due to binding (*β*′ *VT*), the same process as *βVT*, where *β* ≠ *β*′ allows for different measurement units of assays used to detect virus. IFN (*F*) induces the protective transition from *T* to *R* at rate *ϕFT* and resistant cells (*R*) lose protection, reverting to susceptible target cells at a rate *ρ* [28].

Infected cells (*I*) increase due to the infection of target cells by virus (*β*′ *VT*) and die at a (base) rate *δ*. The term *κIF* models the killing of infected cells by IFN-activated NK cells [28]. IFN (*F*) is modelled using simple dynamics that only include production (*qI*) and natural decay (*dF*) [36]. Antibodies (*A*) are produced by activated B cells. We model the proportion of activated B cells by state *B*. The activation of B cells is induced by an increase in *V*. Parameter values and their justification are given in Table 1.

**Table 1:** Parameter values for the three models. The units of *V*, *F* and *A* are denoted as *u*_*v*_, *u*_*F*_ and *u*_*A*_ respectively. *T*, *I*, and *R* have the same unit of *u*_*T*_; the number of cells. Time (*t*) has a unit of days (*d*). Some units are symbolised, as our study is highly qualitative and thus substantially independent of the choice of units. Such units would need to be transformed for different experimental protocols. “varied” indicates that the parameter was assigned different values for different simulations, with the value/s specified whenever necessary. Other parameters are taken or estimated from the literature (references are provided beside those parameter values), and others chosen such that 1) the viral load during infection experiences at least a three orders of magnitude increase and peaks at around the second day post infection [10, 17, 22, 28]; 2) IFN is maximally activated at around 2–4 days post infection [28]; and 3) antibodies are observable (i.e. rise above a lower detection threshold limit) later than six days post infection [24, 35].

Par.	Description	Model 1	Model 2	Model 3	Units
*p*	viral production rate	0.35			$u_{V}u_{T}^{-1}d^{-1}$
*c*	viral clearance rate	20 [28]			*d* ^−1^
*μ*	rate of viral neutralisation by binding of antibodies	0.2			$u_{A}^{-1}d^{-1}$
*μ*′	rate of antibody consumption by binding to virions	0.04			$u_{V}^{-1}d^{-1}$
*β*	rate of viral consumption by binding to target cells	5 × 10^−7^			$u_{T}^{-1}d^{-1}$
*β*′	rate of conversion from target cells to infected cells by viral infection	2 × 10^−5^			$u_{V}^{-1}d^{-1}$
*g*	basal growth rate of healthy cells	0.8			*d* ^−1^
*C*_*t*_	total number of epithelial cells in the ferret URT	7 × 10^7^ [29]			*u*_*T*_
*δ*	death/removal rate of infected cells	3			*d* ^−1^
*ϕ*	rate of IFN-induced conversion from target cells to virus-resistant cells	0.14	0	0	$u_{F}^{-1}d^{-1}$
*ρ*	rate of recovery from virus-resistant cells to target cells	0.05	0	0	*d* ^−1^
*ξ*	death rate of virus-resistant cells	0.1	0	0	*d* ^−1^
*s*	sensitivity of viral production rate to IFN	0	varied	0	——
*κ*	killing rate of infected cells by NK cells	0	0	varied	$u_{F}^{-1}d^{-1}$
*q*	IFN production rate	varied			$u_{F}u_{T}^{-1}d^{-1}$
*d*	IFN degradation rate	2 [28, 36]			*d* ^−1^
*m*_1_	rate of virus-induced B cell activation	1 × 10^−4^			$u_{V}^{-1}d^{-1}$
*m*_2_	rate of B cell deactivation	0.01			*d* ^−1^
*m*_3_	antibody production rate	12000			*u*_*A*_ *d* ^−1^
*r*	antibody degradation rate	0.2 [16, 24]			*d* ^−1^

For a clearer comparison between the different hypothesised mechanisms by which the innate response contributes to viral control, we consider three models of single virus, each of which includes only one of the mechanisms shown in Fig 4:

- **Model 1**: including an IFN-induced virus-resistant state of the target cells (by letting *s* = 0 and *κ* = 0),
- **Model 2**: including an IFN-induced diminished viral production rate (by letting *ϕ* = *ρ* = *ξ* = 0 and *κ* = 0),
- **Model 3**: including killing of infected cells by IFN-activated NK cells (by letting *s* = 0 and *ϕ* = *ρ* = *ξ* = 0).

Most importantly, for these three models, the terms of antiviral action appear in different equations. The virus-resistant terms appear directly in the equation for *dT*/*dt* and thus modulates the viral load (*V*) in an indirect way (Model 1). Similarly, killing of infected cells by NK cells (*κIF*) exerts an indirect control on viral production by changing the infected cell kinetics (Model 3). In contrast, Model 2 assumes direct control of viral production by IFN (due to the term *pI*/(1+*sF*)). Thus, we capture the diversity of plausible viral control mechanisms, in particular both indirect and direct pathways.

### Models for re-exposure viral kinetics

In order to capture the kinetics of primary–challenge infection experiments, we introduce a re-exposure model in which we assume that the two different viruses share the same source of target cells and IFN, but induce distinct and non-crossreactive antibody responses:

$$\begin{array}{c} \frac{dT}{dt}=g\left(T+R\right)\left(1-\frac{T+R+I_{1}+I_{2}}{C_{t}}\right)-\beta_{1}^{\prime }V_{1}T-\beta_{2}^{\prime }V_{2}T+\rho R-\phi FT, \end{array}$$ (8)

$$\begin{array}{c} \frac{dR}{dt}=\phi FT-\rho R-\xi R, \end{array}$$ (9)

$$\begin{array}{c} \frac{dF}{dt}=q_{1}I_{1}+q_{2}I_{2}-dF, \end{array}$$ (10)

$$\begin{array}{c} \frac{dV_{1}}{dt}=\frac{p_{1}I_{1}}{1+s_{1}F}-c_{1}V_{1}-\mu_{1}V_{1}A_{1}-\beta_{1}V_{1}T, \end{array}$$ (11)

$$\begin{array}{c} \frac{dI_{1}}{dt}=\beta_{1}^{\prime }V_{1}T-\delta_{1}I_{1}-\kappa_{1}I_{1}F, \end{array}$$ (12)

$$\begin{array}{c} \frac{dB_{1}}{dt}=m_{11}V_{1}\left(1-B_{1}\right)-m_{21}B_{1}, \end{array}$$ (13)

$$\begin{array}{c} \frac{dA_{1}}{dt}=m_{31}B_{1}-r_{1}A_{1}-\mu_{1}^{\prime }V_{1}A_{1}, \end{array}$$ (14)

$$\begin{array}{c} \frac{dV_{2}}{dt}=\frac{p_{2}I_{2}}{1+s_{2}F}-c_{2}V_{2}-\mu_{2}V_{2}A_{2}-\beta_{2}V_{2}T, \end{array}$$ (15)

$$\begin{array}{c} \frac{dI_{2}}{dt}=\beta_{2}^{\prime }V_{2}T-\delta_{2}I_{2}-\kappa_{2}I_{2}F, \end{array}$$ (16)

$$\begin{array}{c} \frac{dB_{2}}{dt}=m_{12}V_{2}\left(1-B_{2}\right)-m_{22}B_{2}, \end{array}$$ (17)

$$\begin{array}{c} \frac{dA_{2}}{dt}=m_{32}B_{2}-r_{2}A_{2}-\mu_{2}^{\prime }V_{2}A_{2}. \end{array}$$ (18)

An additional subscript (1 or 2, following the existing ones if there is already a subscript like *m*_1_, *m*_2_ and *m*_3_) has been introduced for all the relevant variables and parameters to indicate the primary and challenge viruses. Due to a paucity of experimental data, all parameters for the two viruses are assumed to be equal unless otherwise specified.

As for the single-virus model, we also extend the re-exposure model to three models, each of which includes only one of the innate immune response mechanisms:

- **Model R1**: including an IFN-induced virus-resistant state of the target cells (by letting *s*_1_ = *s*_2_ = 0 and *κ*_1_ = *κ*_2_ = 0),
- **Model R2**: including an IFN-induced diminished viral production rate (by letting *ϕ* = *ρ* = *ξ* = 0 and *κ*_1_ = *κ*_2_ = 0),
- **Model R3**: including killing of infected cells by IFN-activated NK cells (by letting *ϕ* = *ρ* = *ξ* = 0 and *s*_1_ = *s*_2_ = 0).

### Methods for numerical simulations and statistical analyses

The ordinary differential equation (ODE) models were solved using MATLAB’s *ode15s* ODE solver (The MathWorks, Natick, MA). We set an absolute tolerance of 10^−12^ on all variables for accuracy. For the single-exposure models (e.g. Eqs 1—7), initial conditions were *V* = 1, *T* = *C*_*t*_ with all other variables set to zero at *t* = 0. For the re-exposure models (e.g. Eqs 8—18), initial conditions were *V*_1_ = 1, *T* = *C*_*t*_ with all other variables set to zero at *t* = 0. *V*_2_ = 1 was then introduced at the time of challenge. The resolution of the simulated time series shown in the figures was set to be one hundred points per day. When analysing the re-exposure model results, we introduced an indicator, the moving-correlation (MC) coefficient, defined to be the correlation coefficient of a subset of the time series within a moving window, to indicate the periods where the rates of change of the two viral loads were either synchronised or desynchronised. The moving-window was set to be 0.2 days (corresponding to 20 points based on the time series resolution), which we found was sufficient to correctly capture both the relationships and the turning points (smaller values do not further improve the determination of phase-transition points). Determination of these critical phase-transition times was also confirmed by observation based on the time course of solutions. The first peak of the secondary viral infection separating Phase 1 and 2 (defined in the Results) was determined by finding the points where *dV*_2_/*dt* = 0. MATLAB code is provided in the *Supporting Information*.

## Results

We first explore the dynamics of single virus infection. We study how Models 1–3 differ in their explanations of immune-mediated viral control, through both numerical simulation and consideration of the structural properties of the models. Based on these analyses, we then move on to study the re-exposure data using Models R1–R3. With our emphasis on exploring how different viruses’ abilities to stimulate the innate-immune response may induce viral hierarchies, we keep the underlying kinetic properties of the primary and challenge viruses equal.

### IFN-mediated induction of a resistive state for target cells still results in viral control via target-cell depletion

The level of IFN should significantly influence the kinetics of viral infection based on the (three) model formulations. The control of the level of IFN is achieved by using different IFN production rates (*q*). Here we examine how the behaviour of the three models changes for different rates of IFN production and how the models compare to one another.

For Model 1, a higher rate of IFN production (and thus a higher attained IFN level) is able to maintain a considerable level of healthy cells in the virus-resistant state, which in turn facilitates a relatively rapid replenishment of target cells immediately following the control of viral infection. However Model 1 fails to prevent the occurrence of a temporary depletion of target cells (see S1 Fig in the *Supporting Information*). Indeed, target-cell depletion remains the underlying mechanism for control. This is not a surprising result, as in Model 1 increasing IFN leads to a decrease of the term −*ϕFT* in Eq 2, which facilitates the consumption of target cells.

**Fig 5:**
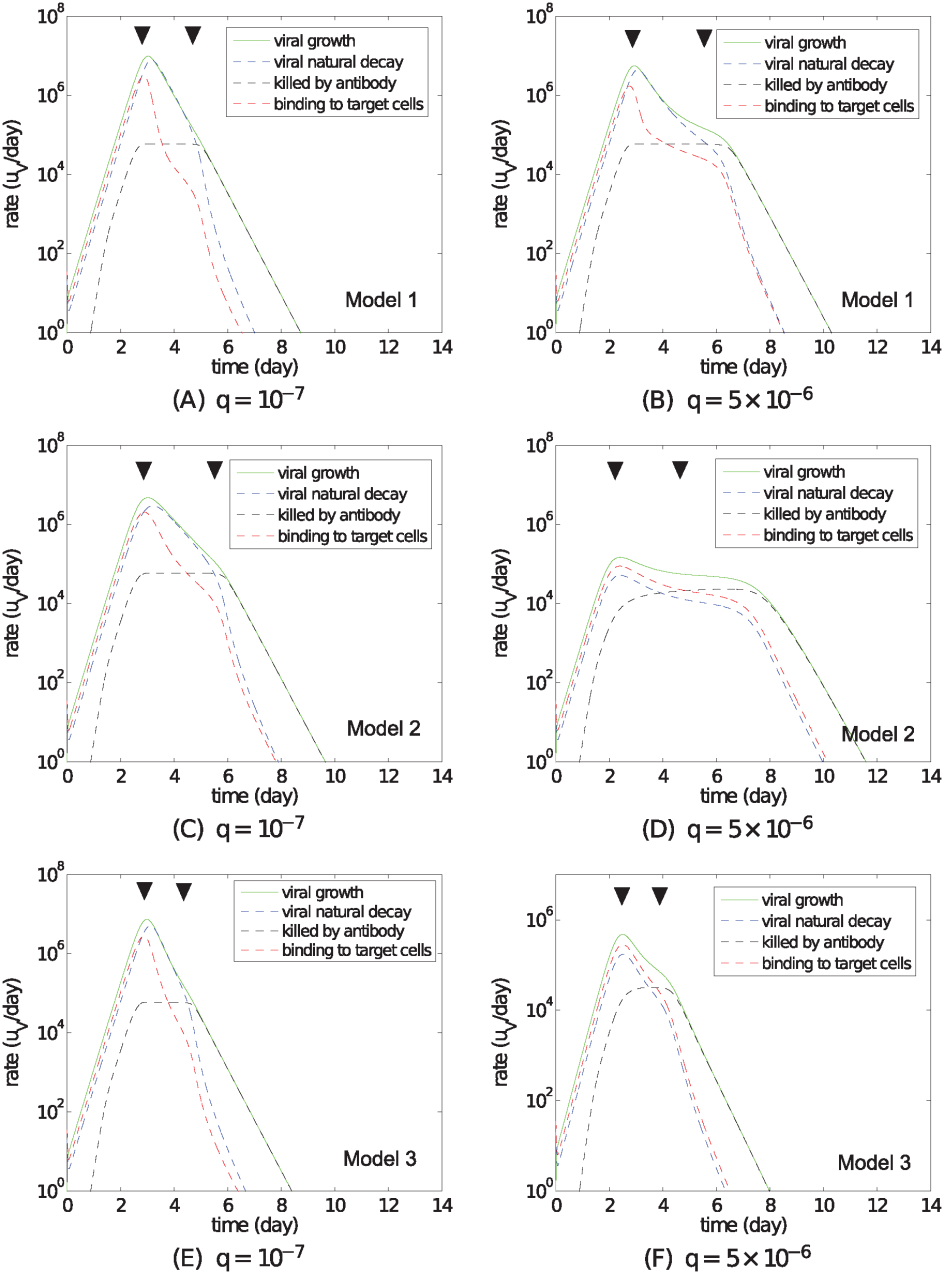
Stage analysis of the three models with different antiviral mechanisms. For each model, we compute the solutions for two different IFN production rates, *q* = 10^−7^ and *q* = 5 × 10^−6^ (shown in S1–S3 Figs in the *Supporting Information*). Focusing on understanding the mechanisms of control of viral load, we present here the time series for the four terms on the right-hand side of Eq 1, *pI*/(1+*sF*) (viral growth), *cV* (viral natural decay), *μAV* (killed by antibody), and *βVT* (binding to target cells), which are calculated based on the model solutions and represent the contribution of each term to the change of viral load (*dV*/*dt*). Model 1: panels (A) and (B); Model 2: panels (C) and (D); Model 3: panels (E) and (F). For each of the cases shown, we use black trangles to roughly indicate three consecutive phases, which are characterised by the dominant factors involved in controlling the change of viral load.

A detailed study of the change in viral load for both low and higher levels of IFN production (*q*) is shown in Fig 5A and 5B, where the change of viral load (*dV*/*dt* in Eq 1) is decomposed into its four components (appearing on the right-hand side of Eq 1), whose relative contributions to the change in viral load vary by the stage of infection. Both figures show that the single virus infection may be deconstructed into three distinct stages. In the first (“early”) stage of infection (0–2 days) virions are primarily consumed by binding to the target cells (due to an almost full target cell pool) and natural decay, whereas the contribution from antibody is negligible. The second stage (3–5 days) features a significant drop in the term for binding to target cells, which confirms that high IFN levels do not prevent a temporary depletion of the target cells for Model 1. In the last stage, starting around day 5, antibodies begin to dominate the removal of virions.

In contrast to Model 1 in which target-cell depletion is the primary mechanism of viral control, both Model 2 and Model 3 are able to maintain a relatively high level of target cells when a sufficiently high IFN production rate is assumed (S2B and S3B Figs). The conservation of a high level of target cells is also clearly reflected by Fig 5D and 5F wherein the curve representing binding to target cells (*βVT*) does not show the quick drop evident in Model 1’s dynamics during the second stage post infection. This implies that the decrease in viral load in the second stage for Models 2 and 3 with a larger IFN production rate is driven by mechanisms other than effective limitation in the number of target cells.

When the IFN production rate is small (*q* = 10^−7^), as shown in Fig 5A, 5C and 5E, all three models converge (as expected) to generate qualitatively the same dynamical behaviours as from the simplest TIV model lacking an explicit, time-dependent innate immune response (see S4 Fig).

**Fig 6:**
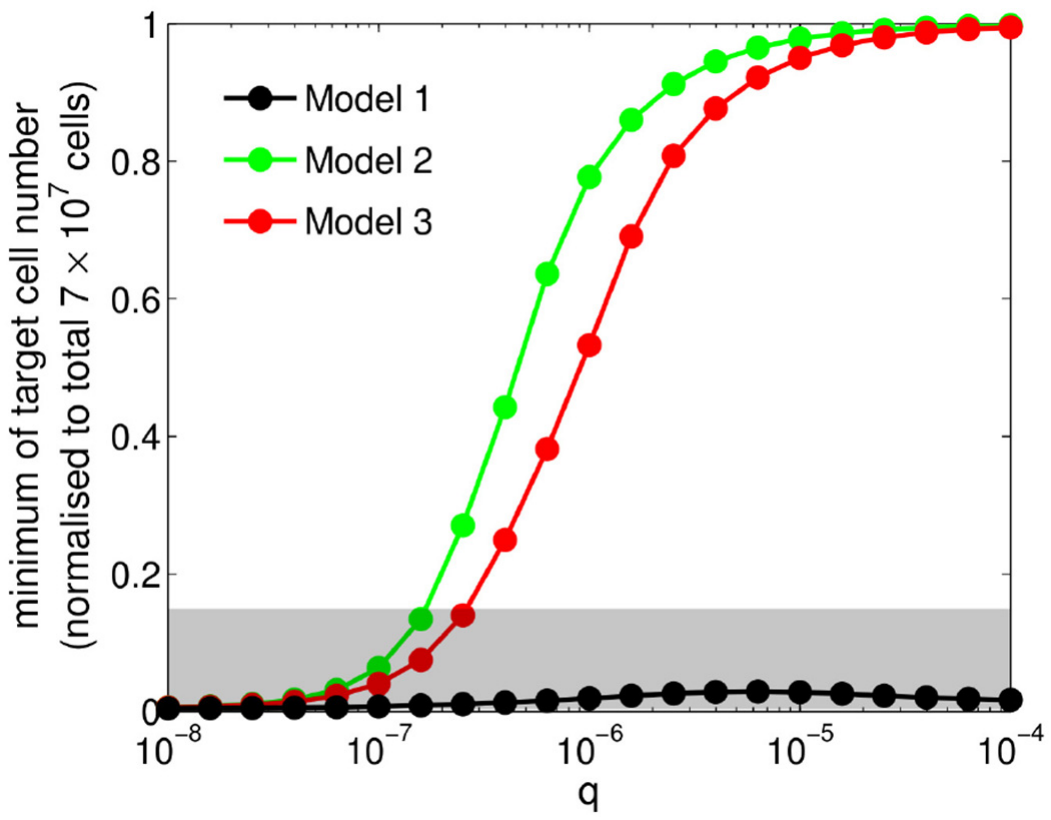
Dependence of target cell depletion on IFN production rate *q* for Models 1–3. For each single virus-strain model, we vary the value of *q* and record the minimum level of target cells (value of *T*) within 7 days post-infection. Models 2 and 3 show a sigmoidal increase as *q* increases from 10^−8^ to 10^−4^, as opposed to Model 1 which always gives a nearly depleted target cell pool. For Model 1, if we allow *q*, *ϕ* and *ρ* to vary (e.g. using a mesh grid of (*q*, *ϕ*, *ρ*) = [0,10^−8:0.2:−4^] × [0,10^−3:0.4:1^] × [0,10^−3:0.5:2^] in this simulation), we find all of the cases give minimum levels of target cells within the grey area. Note that *s* = 1 for Model 2 and *κ* = 3 for Model 3.

To study how target-cell depletion varies with the IFN production rate (*q*) in greater detail, we now explore model behaviour as *q* increases from 10^−8^ to 5 × 10^−5^. With increasing *q*, both Model 2 and Model 3 gradually prevent a temporary depletion of target cells (measured by the minimum of target cell number within the first 7 days post-infection) whereas Model 1 fails to do so (Fig 6; S5–S7 Figs show examples of full time courses for relevant model compartments). Even when allowing the transition rates for the production (*ϕ*) and decay (*ρ*) of IFN to be sampled from the space {(*ϕ*, *ρ*) ∈ [0, 10] × [0, 100]}, we find the minimum target cell number for Model 1 is restricted to lie within the grey region in Fig 6. Note that for some intermediate values of *q* the models may lose their ability to completely clear virus (see S6 and S7 Figs), likely due to a lack of immune components or incorporation of only one innate immune mechanism for each case (see Discussion for further comments). These results confirm that Model 1 primarily utilises target cell depletion for viral control and demonstrate that Models 2 and 3 may also have different dynamical properties depending on the IFN production rate.

### Challenge virus kinetics are strongly influenced by the inter-exposure interval

Having established the mechanisms Models 1–3 use to control viral infection, we now move onto an examination of the behaviours of the re-exposure models, in which two viruses (the primary and challenge viruses) are introduced consecutively with an inter-exposure interval (IEI). We first study Model R1 in detail, focusing on how the model recaptures the clear dependence upon the IEI shown in the experimental data (Figs 1 and 2). We then present the results of the other two re-exposure models based on that analysis, and through a comparison evaluate the differences between the three models.

**Fig 7:**
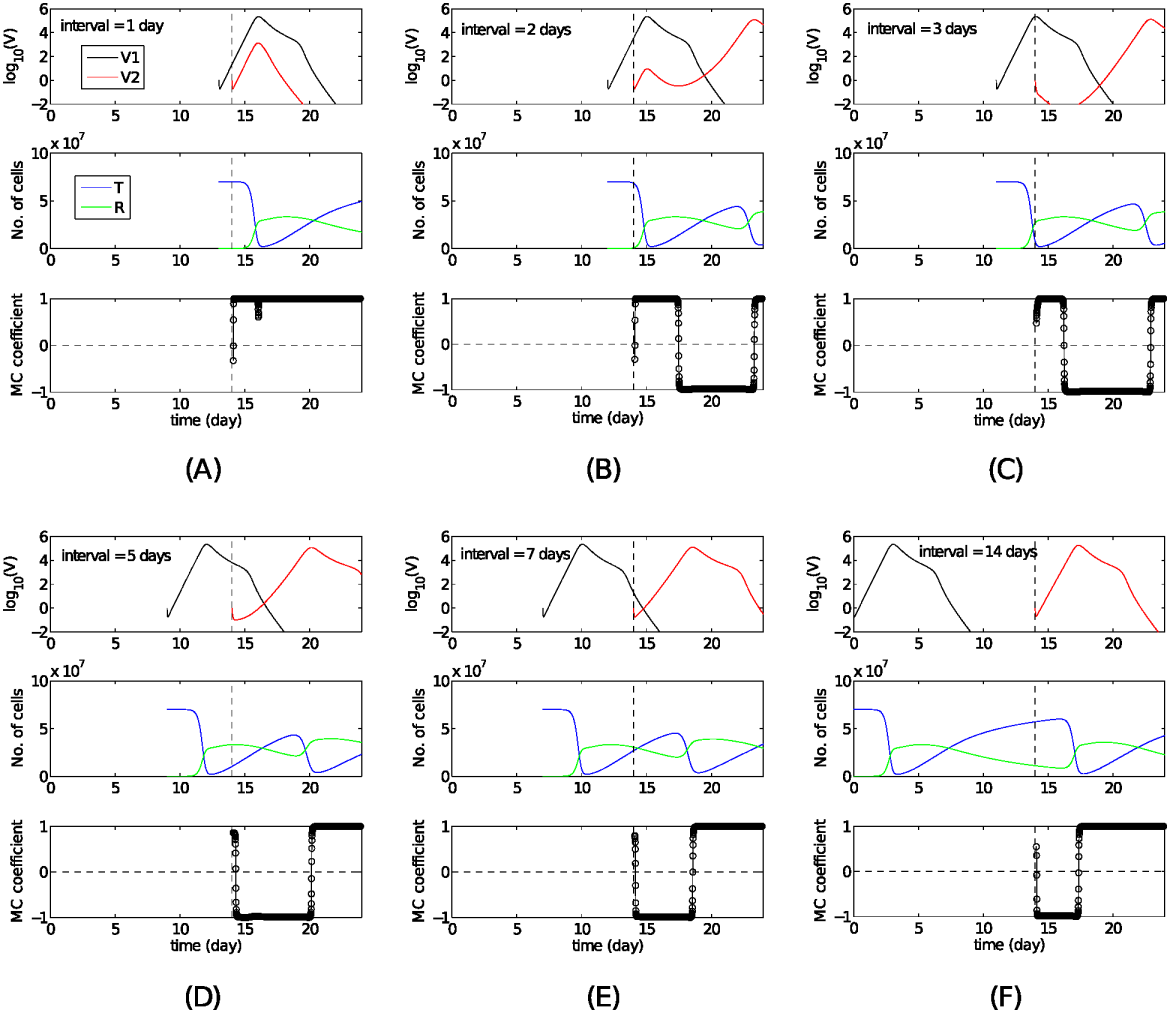
Solutions of Model R1 for different IEI. Primary and challenge viral kinetics, target (and resistant) cell kinetics and the moving-correlation (MC) coefficient for Model R1 as a function of the exposure interval varied from 1 day (A) to 14 days (F). Initial conditions are *V*_1_ = 1, *T* = *C*_*t*_ with all other variables set to zero at *t* = 0. *V*_2_ = 1 is then introduced at *t* = 14 days indicated by the vertical dashed lines. We assume *q*_1_ = *q*_2_ = 5 × 10^−6^. The MC coefficient, facilitating an overall understanding of the vial load time series, is used to indicate synchronisation (+1)/desynchronisation (−1) of the two simulated viral loads and more importantly to identify where the synchronous trend changes (see the “Methods for numerical simulations and statistical analyses” section for details).

Fig 7 shows that the solutions of Model R1, in particular the viral kinetics of the second virus (red curves), change dramatically as the IEI increases from 1 day (A) to 14 days (F). These changes are summarised and can be explained as follows:

- For a 1 day interval (Fig 7A), the two viruses undergo an initially synchronised increase followed by a synchronised decrease (i.e. co-infection). The synchronised increase occurs in the very early stage of infection when target cell numbers remain sufficiently high, corresponding to the first stage of single-virus infection as examined in the previous section. Following target cell depletion, both viruses decrease and are eventually cleared by strain-specific antibody. The dynamics are akin to those for a single virus infection.
- For a 2 day interval (Fig 7B), a short period of synchronised increase is followed by a synchronised decrease (indicated by the MC coefficient of 1). However, this is then followed by a desynchronised period (MC coefficient of -1) wherein the primary virus is cleared while the challenge virus grows once more. During the initial period of synchronised growth the challenge virus’ load is two orders of magnitude smaller than that of the primary virus and the challenge virus in Fig 7A. This may be understood by the strong depletion of target cells by this time. Such a low viral load does not effectively activate antibody production for that virus so that following the brief drop (synchronised with the first virus due to temporary depletion of target cells) the challenge virus increases again with the replenishment of the target cell population.
- For a 3 day interval (Fig 7C) there is no period of synchronised growth. Rather, a synchronised decrease appears immediately following challenge with the second virus due to the temporary depletion of target cells, which is the key feature of the second stage for the single-virus infection (recall Fig 5). Challenge with the second virus during this second stage of the primary virus infection (around 3–5 days) leads to qualitatively the same behaviours (shown later). Similar to Fig 7B, after the initial drop, the second virus experiences a full cycle of infection.
- For an IEI over 6 days (Fig 7E and 7F) the primary virus has essentially been cleared at the time of challenge (stage three of the primary virus infection). During this stage target cell numbers are increasing, enabling immediate infection by the second virus.

**Fig 8:**
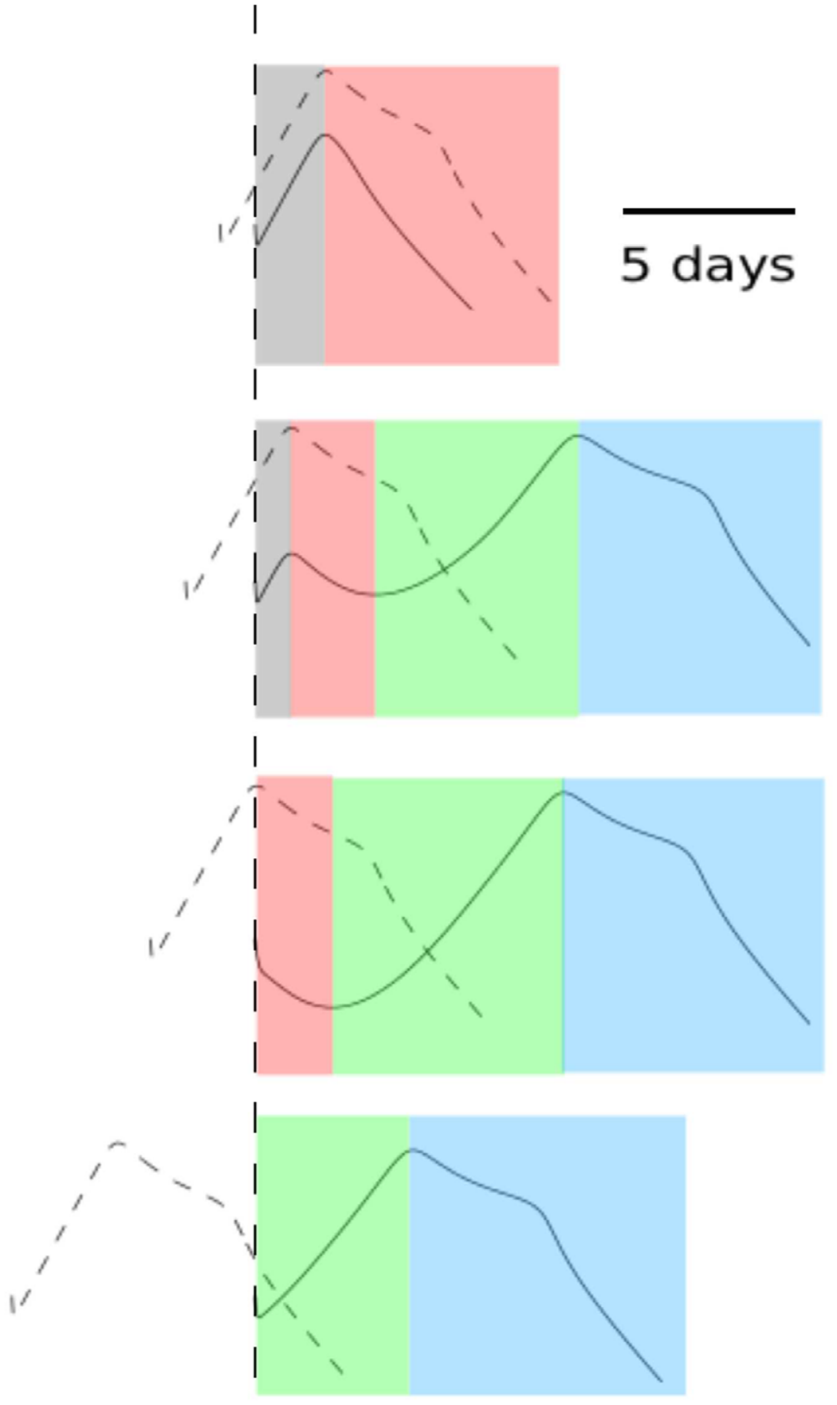
Phase decomposition of the re-exposure behaviours. The representative cases selected from Fig 7 correspond to (from top to bottom) the IEI of 1 day, 2 days, 3 days and 7 days respectively. Dashed curves indicate the viral load of the primary virus infection and solid curves indicate the viral load of the second challenge. Four phases are observed in order. Phase 1 (grey) is the co-infection phase. Phase 2 (red) is a phase where a synchronised drop is observed (the similar case also spotted in the data, see Fig 1). Phase 3 (green) is a desynchronised phase where the first virus experiences a decrease due to antibody neutralisation whereas the second virus increases due to the replenishment of the target cell pool. Phase 4 (blue) is the final period where both viruses are neutralised by their specific antibodies. A scale bar of 5 days indicates the length of time.

Fig 8 summarises all the observed behaviours of Model R1 and indicates the different phases including productive co-infection (Phase 1, grey), an early synchronised decrease (Phase 2, red), a desynchronised phase (Phase 3, green) and final removal of the challenge virus (Phase 4, blue). Importantly, all four phases can be easily mapped to experimental data (Figs 1–3), and always appear in the described order. We will see later that the other two re-exposure models, although exhibiting qualitative differences from Model R1, do not alter the order established here.

**Fig 9:**
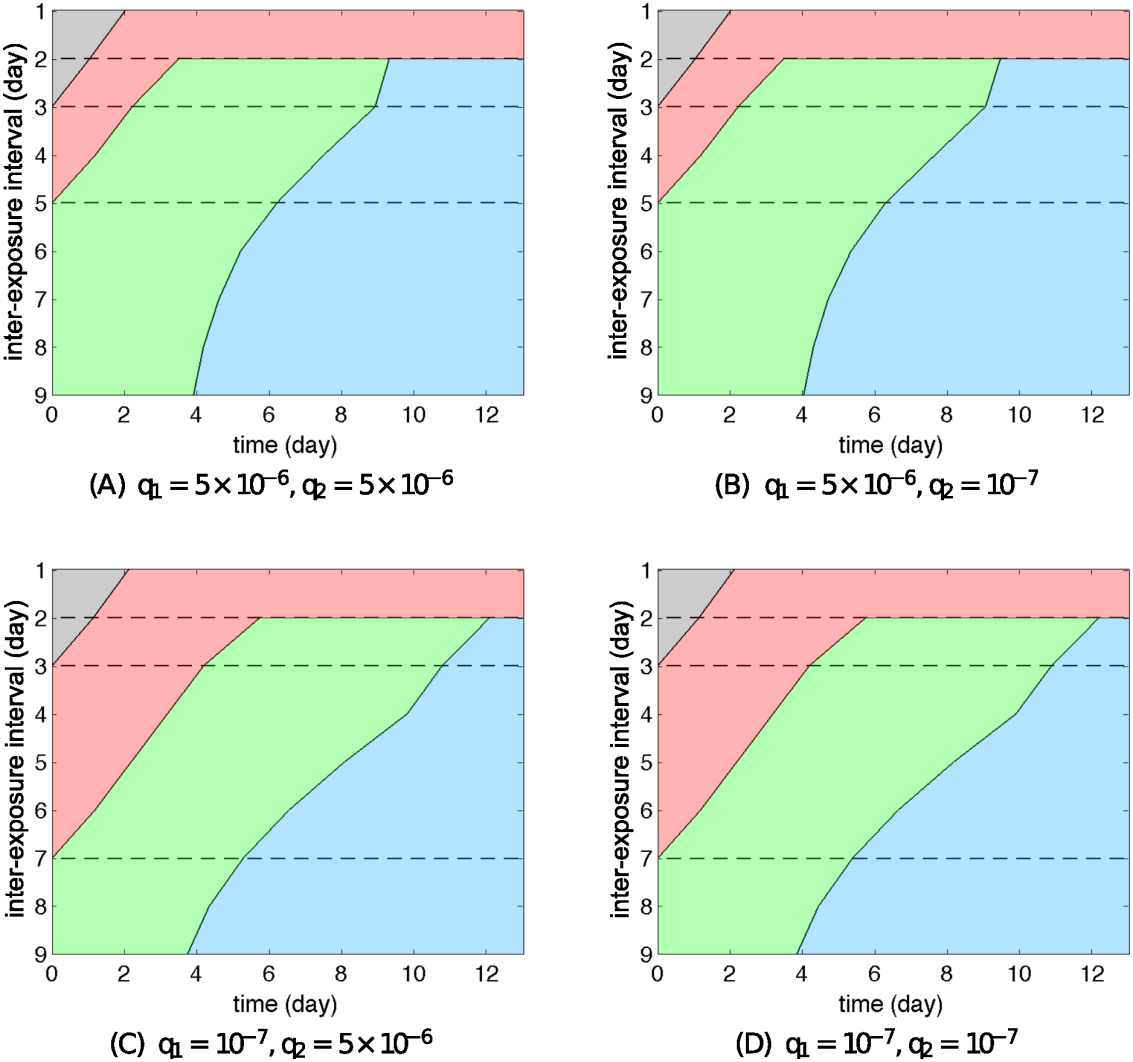
Re-exposure behaviour of Model R1 for different IFN production rates. A smaller IFN production for the primary virus for Model R1 does not lead to any qualitative difference, in terms of the dependence of model behaviours for the challenge virus on the IEI, from the case of very large IFN production rate of the first virus. The pattern is also independent of the choice of *q*_2_. The meaning of each colour is explained in Fig 8. They all exhibit four types of behaviours (seen vertically, separated by dashed lines) and within each type the phase decomposition and their order are preserved.

As we have analysed, the infection dynamics are closely related to the stage of the primary virus infection at the time of challenge with the second virus, explaining the strong influence of the exposure interval in both the experimental observations and model outputs. All of our observations can be summarised in a single figure (Fig 9A). Reading horizontally, we see that the four phases (separated by colours) appear in order through time. Viewed vertically, the figure shows that the choice of the IEI strongly affects the qualitative behaviours of the re-exposure model (distinguished by dashed lines). Importantly, all regions of this plane may be classified as one of the four phases, suggesting a complete picture has been obtained through this classification procedure. Because of the concise nature of this method of showing re-exposure results, this type of figure will be used to show further results for the alternative re-exposure models (Models R2 and R3).

### The hierarchy of viral infection is reproduced by the re-exposure models and provides new insight into model selection

Continuing with the study of Model R1, we now examine whether it can generate qualitatively different re-exposure behaviours (i.e. generate patterns different from that in Fig 9A) by only changing the IFN production rates of the two viruses, *q*_1_ and *q*_2_. As shown in the previous section on single infection, different IFN production rates drive different model behaviours. A very small IFN production (e.g. 10^−7^) resembles a model that lacks any IFN-induced protective effect whereas a model with a relatively large IFN production rate (e.g. 5 × 10^−6^) shows a significant conversion of target cells to the virus-resistant state. Results are given in Fig 9 where four different combinations of values for *q*_1_ and *q*_2_ are examined. S10 Fig presents further combinations of values for *q*_1_ and *q*_2_ drawn from a wider range. All sub-figures (Fig 9; S10 Fig) show a qualitatively similar pattern. In particular, regardless of the level of IFN production (and thus regardless of whether the resistant state is introduced or not), all show the existence of Phase 2 (red) which characterises the inhibitory effect of the primary virus infection on the challenge virus. Based on our previous analyses of a single viral infection, this is a result of target cell depletion which cannot be avoided by solely introducing the virus-resistant state. Thus, Model R1 (with all other parameters fixed and equal for the two viruses) fails to reproduce the hierarchy of viral infection shown in the data; e.g. primary infection with A(H1N1)pdm09 strongly inhibited influenza B virus challenge dynamics (Fig 1), whereas the latter showed a very limited ability to inhibit the former (Fig 2). The subtle quantitative differences visible in Fig 9 and S10 Fig are understood by considering that a larger *q*_1_ (regardless of *q*_2_) induces a larger virus-resistant cell population (*R*) and so more rapid replenishment of the target cell pool. Consequently, an increased *q*_1_ leads to a shorter duration of Phase 2 (IEI of day 3–5) as also shown in S1 Fig.

**Fig 10:**
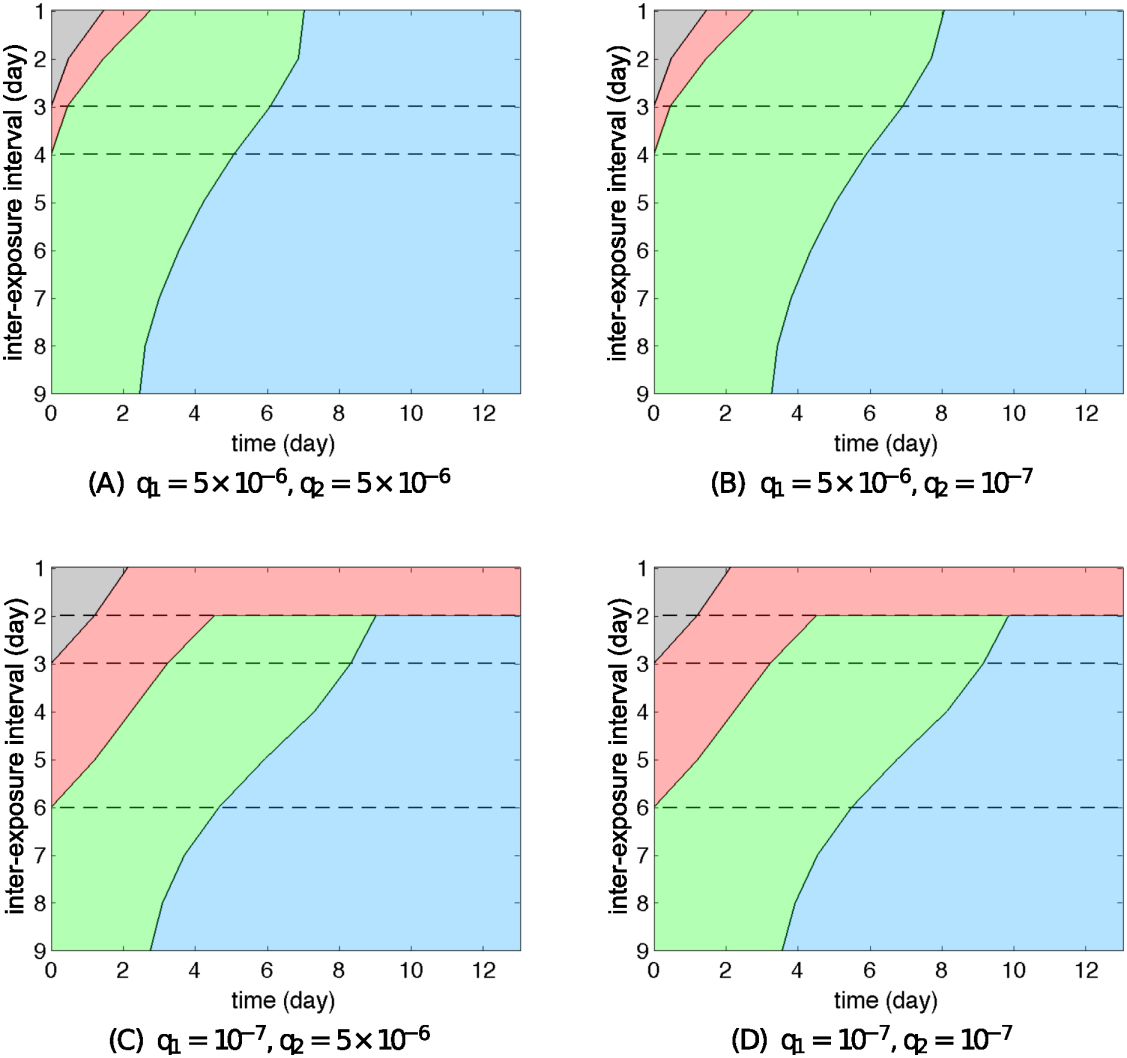
Re-exposure behaviour of Model R2 for different IFN production rates. Different IFN productions of the first virus for Model R2 lead to qualitatively different patterns of re-exposure simulation. The pattern is independent of the choice of *q*_2_. The meaning of each colour is explained in Fig 8. In spite of different patterns, the order of phase is preserved for any cases. We assume here that *s*_1_ = *s*_2_ = 1.

**Fig 11:**
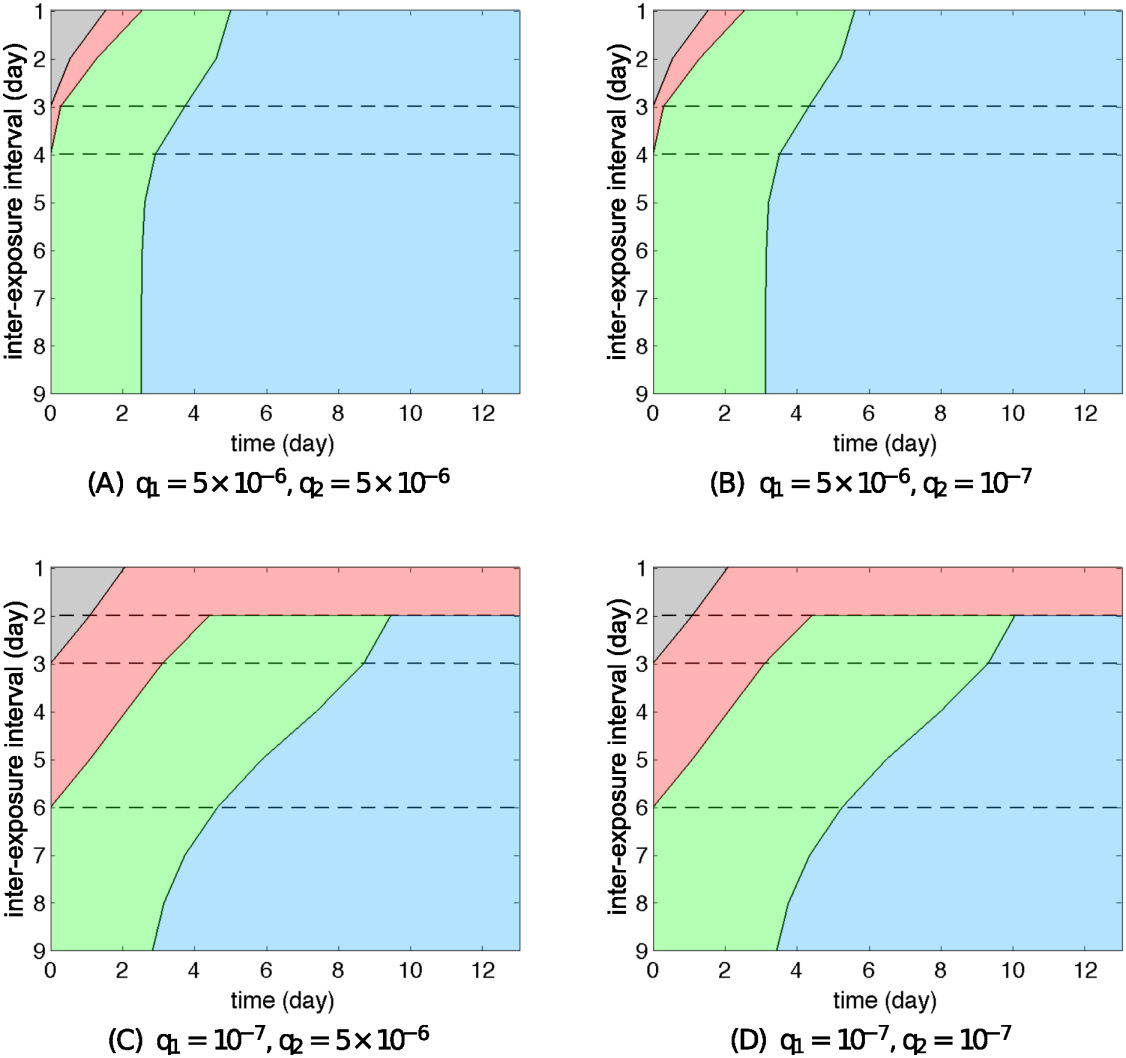
Re-exposure behaviour of Model R3 for different IFN production rates. Different IFN productions of the first virus for Model R3 lead to qualitatively different patterns of re-exposure simulation. The pattern is independent of the choice of *q*_2_. The meaning of each colour is explained in Fig 8. In spite of different patterns, the order of phase is preserved for any cases. We assume here that *κ*_1_ = *κ*_2_ = 3.

Moving on to consider Models R2 and R3, two different patterns emerge when varying the IFN production rate, as demonstrated by comparing the top row to the bottom row in Figs 10 and 11 for Models R2 and R3 respectively (also see S8 and S9 Figs for examples of time courses of the solutions). These patterns successfully reproduce the hierarchy of viral infection observed from the experimental data (Figs 1 and 2), as we now illustrate. Consider the case that A(H1N1)pdm09 strongly stimulates the immune response (high *q*) and influenza B provides weaker stimulation (low *q*). Then if we take the primary virus to be A(H1N1)pdm09 (*q*_1_ = 5 × 10^−6^) and the challenge virus to be influenza B (*q*_2_ = 10^−7^) we observe that A(H1N1)pdm09 delays infection with influenza B for short IEIs (Fig 10B and S8 Fig). Conversely, if influenza B is administered first (*q*_1_ = 10^−7^), then challenge with A(H1N1)pdm09 (*q*_2_ = 5 × 10^−6^) results in co-infection for short IEIs (Fig 10C). Results for more combinations of values for *q*_1_ and *q*_2_ drawn from a wider range are provided in S11 Fig (for Model R2) and S12 Fig (for Model R3). In all scenarios for Models R2 and R3, high *q*_1_ prevents depletion of the target cell pool during the primary virus infection (see Fig 6). While exerting a weak delay on the challenge virus for an IEI of 1–3 days (seen in both data and simulation results), the continued availability of target cells allows for productive replication, preventing the system from displaying Phase 2 dynamics. Similar to Model R1, we observe that the patterns are primarily dominated by the IFN production rate of the first virus but nearly independent of that of the second virus (S10–S12 Figs).

In summary, our three models—with their alternative hypothesised mechanisms for the action of the innate response leading to viral control—are each capable of capturing the dynamics of a single virus infection and the main features of primary–challenge experiments. However, Model R1, with its reliance upon the virus-resistant state fails to reproduce the hierarchy of viral infection (i.e. it always produces Phase 2 dynamics). For the other two mechanisms—a decreasing viral production rate (Model R2) or an induced killing of infected cells by NK cells (Model R3)—we have shown that both are able to reproduce all the behaviours including the hierarchy of viral infection observed in the experimental data. We have made these observations based on the assumption that the viruses’ underlying kinetic properties are the same and that their differing ability to induce IFN production is the mediator of observed difference. In the Supporting Information (S13 and S14 Figs), we extend our study by exploring some alternative models in which other virus-immunity parameters are allowed to vary (in addition to the IFN production rate), and demonstrate that Models R2 and R3 can still reproduce the observed hierarchies, while Model R1 remains reliant upon target-cell depletion and so is less capable of capturing the observations.

### Stochasticity may explain the complete blocking of the challenge virus at short inter-exposure intervals

**Fig 12:**
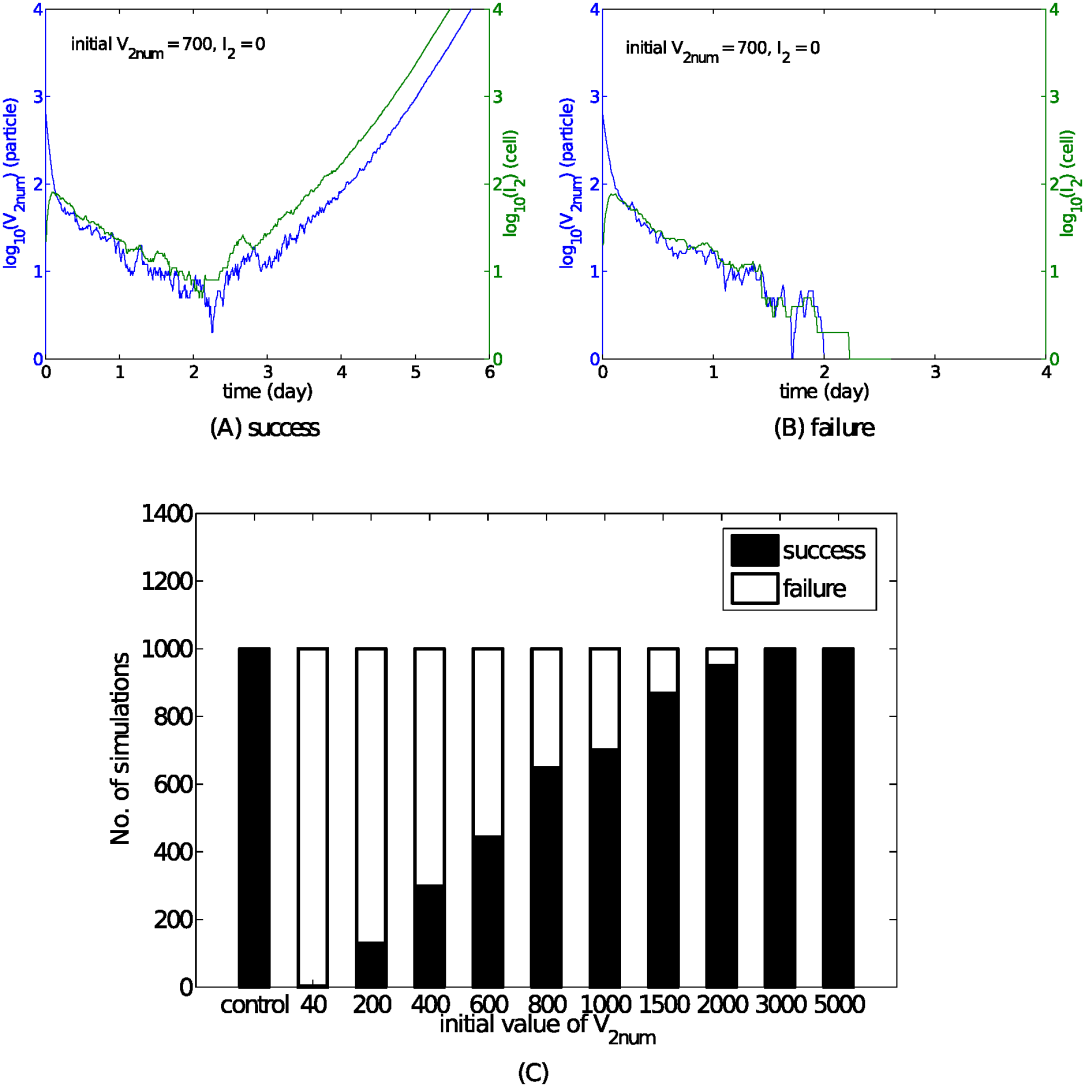
Stochastic simulations show an initial decrease in viral load down to a relatively low level could lead to stochastic extinction, which cannot be explained by deterministic models. With an initial 700 virions (which are far larger than an initial number of 40 virions used in the deterministic model, because *V*_2_ = 1 is equivalent to *V*_2*num*_ = 40 virions) in the second viral inoculation, the virus could survive (A) or go extinct (B). *V*_2*num*_ denotes the number of virions for the second inoculation and *I*_2_ is the number of cells infected by the second type of virus. The stochastic model used here is derived from the model used to simulate Fig 6C with an IEI of 3 days (see the *Supporting Information* for details). The horizontal axes indicate the days after the infection with the second virus (i.e. 0 here corresponds to day 14 in Fig 7C). Panel (C) shows dependence of failure rate of the second viral infection on the initial number of re-challenged virions. A success event is defined to be an event where the viral number can exceed 10000 within 10 days following the re-challenge. The control case indicates the single-virus infection with an initial number of 40 virions (i.e. a stochastic simulation of Model 1). The IEI is still fixed to be 3 days.

We have shown that the re-exposure model can successfully reproduce the phenomena of co-infection and delay (Fig 3). However, we also observe complete blocking of the challenge virus following primary infection in some circumstances (Fig 1, IEIs of 3 and 7 days, and Fig 3). Although the reason for complete inhibition remains unclear, that it only occurs in some of the experimental replicates [10] suggests that stochastic effects in terms of viral dynamics may be important. We hypothesise that failure of the challenge virus may occur when the number of virions (*V*_2*num*_) drops to a sufficiently low value such that stochastic effects become dominant [37]. For example, the case of an initially synchronised decrease (see Fig 3 and Fig 7C) makes our hypothesis possible. Here we examine this by using a stochastic model derived from the deterministic model used to generate Fig 6C (see the *Supporting Information* for details on the stochastic model, its implementation and parameterisation). Fig 12 shows that, although the solution of the deterministic model shows a rebound in viral load, the stochastic model results in two possible classes of solution: delayed infection with the second virus (“success”) or blocked infection with the second virus (“failure”).

To quantify this stochastic phenomenon, we investigate the dependence of the success/failure rate on the initial number of virions (see Fig 12C). Results show that the failure rate increases as the initial number of virions decreases in the presence of an innate immune response. Although the control case shows a 0% chance (0 out of 1000 simulations) of failure for the primary virus infection with an initial number of 40 virions, the same number of virions in the challenge inoculum is insufficient to generate any re-infection events. However, as the initial number of virions (for both the primary and challenge viruses) increases from 40 to 5000, the chance of generating successful re-infection events increases to 100%, with a half chance of success when approximately 700 virions are present in the inoculum (as used to generate Fig 12A and 12B). This implies that failure could be due to an insufficient number of successfully infecting virions in the challenge virus inoculum, even if this number is sufficient to reliably induce infection with the primary virus.

## Discussion

In this paper, we have investigated the role of innate immunity and its possible mechanisms of action based on both experimental data and mathematical models. Experimental data show that infection with one virus prior to challenge with a second strain can delay/block the second viral infection (Figs 1 and 2 and [10]). We interpret these findings as evidence for a hierarchy in different viruses to induce an innate immune response, and in the role of innate immunity in controlling viral infection [10]. To better understand the possible mechanisms accounting for the hierarchy and some interesting (a)synchronised infections observed experimentally (Fig 3), we constructed and analysed several mathematical models with different IFN-induced immune response mechanisms. Our results show that 1) without other (virus specific) mechanisms at play, a model solely with a virus-resistant state is not able to reproduce the hierarchy of viral infection; and 2) the occurrence of synchronised and desynchronised phenomena is highly dependent upon both the hierarchy of viral infectious ability and the time interval between the consecutive viral inoculations.

In more detail, we have shown that the model solely with a virus-resistant state (Model 1) primarily utilises target cell depletion to control viral growth, independent of the IFN production rate (Fig 6, black curve). The temporary depletion of target cells will strongly limit the growth of any other virus, resulting in the failure to observe a viral hierarchy (Fig 9). In contrast, for the other two mechanisms (Models 2 and 3), sufficient production of IFN efficiently prevents target cells from depletion (Fig 6, green and red curves), providing a foundation for another virus to rapidly replicate (Figs 10 and 11).

Alternative mechanisms for the action of the innate immune response have been adopted by many models in the literature [16–19, 21–24, 26, 28, 38]. Although each has achieved success in unveiling important kinetic parameters of viruses and immunity, their focus on infection with a single virus has not allowed for the investigation of the relative importance of the different immune mechanisms and the hierarchical interaction between virus strains. Based on re-exposure experiments [10], here we have re-examined the immune mechanisms proposed by previous studies and showed that different proposed mechanisms have differing capabilities and limitations in reproducing the re-exposure data.

Our mathematical analysis, in conjunction with re-exposure experiments, not only demonstrates the importance of studying experiments involving multi-strain viral infections but also opens a new paradigm in which to study the immune response to influenza and its role in viral control. We can now expand the ways in which to systematically evaluate the feasibility and relative importance of different hypothesised mechanisms of innate immunity, a knowledge gap highlighted in recent reviews [34, 35]. In this context, our work complements and extends the current literature on the use of co-infection and re-exposure studies, in combination with mathematical modelling, to investigate a range of biological phenomena such as assessment of fitness (e.g. for drug-resistant strains of influenza [32], virulent strains of malaria [39], and escape mutant virus for SIV [40]).

In the main text we have focused on the role of IFN production in generating the viral hierarchy, assuming other parameters are strain independent. Our results suggested that the level of target cells was critical in determining the formation of re-infection patterns as shown in Figs 10 and 11. However, this is not always true when we increase the dimension of model variability. For example, if we assume that the primary and challenge viruses have different sensitivities in their replication rate to IFN (*s*), we may see again the exhibition of Phase 2 dynamics for Model R2 (S13 Fig in the *Supporting Information*). Similar results are also applicable to Model R3 and are shown in S14 Fig, where using different killing rates of infected cells by IFN-activated NK cells (*κ*) yields different re-infection patterns without depleting target cells. We also note that the behaviours of Models 2 and 3 for the simulations of single-virus infection shown in Fig 5 are not continuously dependent upon the IFN production rate. As *q* increases from *q* = 10^−7^ to *q* = 5 × 10^−6^, both models lose their ability to control infection, resulting in a sustained and high viral load for some intermediate values of *q* (see S6 and S7 Figs). On the one hand, this may be due to omission of some key mechanisms (e.g. CD8 T cells dynamics and other specific cytokine functions). On the other hand, since the question of whether the dependence should be continuous or otherwise has not yet been evaluated experimentally, no reliable criterion can be used to judge this mathematical observation. Of course, since each of the models incorporates only one of the innate immune response mechanisms, as per the focus and requirements of our theoretical study, a detailed examination of overall model stability, in which several of the hypothesised mechanisms may be active, was not performed. As such we do not over-elaborate on this issue but leave it to be determined by future work.

Due to a paucity of experimental data, we have assumed throughout this paper that the primary and challenge viruses had the same parameter values except for those parameters directly related to the activity of the innate immune response (i.e. the IFN production rate (*q*); the sensitivity of the viral production rate to IFN (*s*); and the killing rate of infected cells by NK cells (*κ*)). While this simplifying assumption has allowed us to explore the possible drivers of behaviour, and demonstrate the fundamental differences in dynamical properties of the alternative hypotheses, it emphasises again the need for both further experimental data to be collected (e.g. time courses of markers of the immune response) and even tighter coupling of theoretical analyses to those based on more detailed data [10]. In ongoing work, we are applying hierarchical statistical techniques to fit our full models to the available re-exposure data. Through this process, we aim to explore the relative importance of different elements of the innate immune response and how they differ between strains, and furthermore, the contribution, if any, of differences in viral kinetic parameters by strain. Additionally, the observation of delays to peak for the challenge virus in some ferrets [10] may be in principle due to stochastic variation in the effective size of the initial inoculum. While the statistical analyses conducted in [10] suggest that this effect is unlikely to be dominant, variation in the initial conditions of each inoculum will be considered in the statistical study of the re-exposure data.

The models in this paper have been constructed, as have previous models [16, 18, 19, 21, 25, 28, 33], by incorporating both innate and adaptive immune responses into the classic Target cell–Infected cell-Virus model structure. We have made only the minimum extensions necessary to enable re-capture of the observed re-infection kinetic behaviour [10]. Although the models are demonstrably successful in reproducing those data, they by no means capture all of the host-immune interactions observed experimentally for influenza, and for those interactions that they do capture, alternative structural forms may be more appropriate. For example, many studies have shown that CD8 T cells play an important role in the removal of infected cells [41–43]. While the cellular adaptive response is not explicitly captured in our models, we have slightly increased the value of *δ* from the estimated natural death rate of 0.5 − 2 day^−1^ [16, 18, 19] to 3 day^−1^ to (partially) correct for this effect. In addition, part of the NK cell killing effect (*κIF*) could also be considered as a CD8 T cell mediated process. In terms of structure, we have considered only one particular functional description of the innate immune response (e.g. a first-order stimulatory response for IFN) and one form for each of the hypothesised actions of the IFN-mediated response. Alternative structural forms for these, and other, immunological processes may in principle result in different dynamics. In general, any missing components (e.g. CD8 T cells) or mis-specification of the detailed form of how processes act (e.g. IFN-mediated processes) may influence the reliability of our models and their interpretation, and thus further work to collect sufficient data to increase the biological fidelity of models is warranted.

## Supporting Information

S1 Text Equations and MATLAB code for stochastic simulations. (PDF)

S2 Text MATLAB code for deterministic model simulation and generating figures shown in the paper. (PDF)

S1 Fig Solutions of Model 1. Solution of Model 1 for two different IFN production rates, *q* = 10^−7^ and *q* = 5 × 10^−6^, under the initial condition of *V*(0) = 1, *T*(0) = *C*_*t*_ and zeros for all other variables.
(PDF)

S2 Fig Solutions of Model 2. Solutions of Model 2 for two different IFN production rates, *q* = 10^−7^ and *q* = 5 × 10^−6^, under the initial condition of *V*(0) = 1, *T*(0) = *C*_*t*_ and zeros for all other variables. We set *s* = 1 here, which will be treated as a benchmark value for later comparisons.
(PDF)

S3 Fig Solutions of Model 3. Solutions of Model 3 for two different IFN production rates, *q* = 10^−7^ and *q* = 5 × 10^−6^, under the initial condition of *V*(0) = 1, *T*(0) = *C*_*t*_ and zeros for all other variables. We use a benchmark value of *κ* = 3, which lies around the middle of the range estimated from the paper by Pawelek et al. (see reference 28 in the main text)
(PDF)

S4 Fig Solution of a model without innate immunity. A solution of Eqs 1–7 in the main text for zero IFN production, *q* = 0 mimicking no time-dependent innate immunity, under the initial condition of *V*(0) = 1, *T*(0) = *C*_*t*_ and zeros for all other variables. (A) shows time courses of important variables. (B) shows the time series of the four terms on the right-hand side of Eq 1, *pI*/(1+*sF*) (viral growth), *cV* (viral natural decay), *μAV* (killed by antibody), and *βVT* (binding to target cells), are calculated based on the solution and plotted in each panel. They represent the contribution of each term to the change of viral load (*dV*/*dt*).
(PDF)

S5 Fig Dependence of Model 1 behaviour on IFN production rate. Time series show that a temporary depletion of the target cell pool occurs for four different IFN production rates (*q*).
(PDF)

S6 Fig Dependence of Model 2 behaviour on IFN production rate. Time series show that a temporary depletion of the target cell pool occurs for a small IFN production rate (*q* = 10^−7^) but not for a large one (*q* = 5 × 10^−6^). However, for some intermediate values of *q*, the model loses the ability to clear virus, resulting in a sustained elevation of viral load. Possible reasons are explored in the Discussion. We use *s* = 1.
(PDF)

S7 Fig Dependence of Model 3 behaviour on IFN production rate. Time series show that a temporary depletion of the target cell pool occurs for a small IFN production rate (*q* = 10^−7^) but not for a large one (*q* = 5 × 10^−6^). However, for some intermediate values of *q*, the model loses the ability to clear virus, resulting in a sustained elevation of viral load. Possible reasons are explored in Discussion. We use *κ* = 3.
(PDF)

S8 Fig Dependence of Model R2 behaviour on the IEI. Simulations are done by using Model R2. Initial conditions are *V*_1_ = 1, *T* = *C*_*t*_ and zeros for all other variables at *t* = 0 day and *V*_2_ = 1 is then introduced at *t* = 14 days indicated by dashed lines. We use *s*_1_ = *s*_2_ = 1, *q*_1_ = 5 × 10^−6^ and *q*_2_ = 10^−7^. The moving-correlation (MC) coefficient is used to indicate synchronisation/desynchronisation of the two viral loads.
(PDF)

S9 Fig Dependence of Model R3 behaviour on the IEI. Simulations are done by using Model R3. Initial conditions are *V*_1_ = 1, *T* = *C*_*t*_ and zeros for all other variables at *t* = 0 day and *V*_2_ = 1 is then introduced at *t* = 14 days indicated by dashed lines. We use *κ*_1_ = *κ*_2_ = 3, *q*_1_ = 5 × 10^−6^ and *q*_2_ = 10^−7^. The moving-correlation (MC) coefficient is used to indicate synchronisation/desynchronisation of the two viral loads.
(PDF)

S10 Fig Re-exposure behaviour of Model R1 for four different IFN production rates. The value of the IFN production for the primary virus for Model R1 does not lead to any qualitative different patters of infection upon re-exposure. Both small and large values of *q*_1_ result in target-cell depletion. The pattern is also independent of the choice of *q*_2_. The model does not support the observed viral hierachy. The meaning of each colour is explained in Fig 8 in the main text. This figure is an extension of Fig 9 where only two intermediate *q* values are presented.
(PDF)

S11 Fig Re-exposure behaviour of Model R2 for four different IFN production rates. Different IFN production rates for the primary virus for Model R2 lead to qualitatively different patterns of infection upon re-exposure. The pattern is driven by *q*_1_ and independent of the choice of *q*_2_. The meaning of each colour is explained in Fig 8 in the main text. This figure is an extension of Fig 10 where only two intermediate *q* values are presented. We assume here that *s*_1_ = *s*_2_ = 1.
(PDF)

S12 Fig Re-exposure behaviour of Model R3 for four different IFN production rates. Different IFN production rates for the primary virus for Model R3 lead to qualitatively different patterns of infection upon re-exposure. The pattern is driven by *q*_1_ and independent of the choice of *q*_2_. The meaning of each colour is explained in Fig 8 in the main text. This figure is an extension of Fig 11 where only two intermediate *q* values are presented. We assume here that *κ*_1_ = *κ*_2_ = 3.
(PDF)

S13 Fig Dependence of re-infection pattern on IFN-sensitivity parameter *s*. Varying the IFN-sensitivity parameter *s* in Model R2 leads to different patterns from that with the same IFN-sensitivity shown in Fig 9 in the main text. Note that *q* needs also to change accordingly for large *s* to maintain the change of viral load qualitatively similar to experimental observations. Thus, we choose *q* = 5 × 10^−6^ when *s* = 1 and *q*_2_ = 5 × 10^−7^ when *s* = 10. Dashed lines separate different model behaviours in terms of the timing of the second virus challenge.
(PDF)

S14 Fig Dependence of re-infection pattern on killing rate of virus by IFN-activated NK cells *κ*. Varying the killing rate of virus by IFN-activated NK cells *κ* in Model R3 leads to different patterns from that with the same IFN-sensitivity shown in Fig 10 in the main text. Note again that *q* needs also to change accordingly for large *κ* to maintain the change of viral load qualitatively similar to experimental observations. Thus, we choose *q* = 5 × 10^−6^ when *κ* = 3 and *q*_2_ = 1 × 10^−6^ when *κ* = 15. Dashed lines separate different model behaviours in terms of the timing of the second virus challenge.
(PDF)
