# Supplementary material for: Innate Immunity and the Inter-exposure Interval Determine the Dynamics of Secondary Influenza Virus Infection and Explain Observed Viral Hierarchies
Source: PLoS Comput Biol. 2015 Aug 18;11(8):e1004334. doi: 10.1371/journal.pcbi.1004334 (PMC4540579; doi:10.1371/journal.pcbi.1004334)
Supplement: S1 Text — (PDF) [file pcbi.1004334.s001.pdf]

Supporting information for 'Innate immunity and the inter-exposure interval determine the dynamics of secondary influenza virus infection and explain observed viral hierarchies'

Pengxing Cao[1], Ada W. C. Yan[1], Jane M. Heffernan[1,2,3], Stephen Petrie[1], Robert G. Moss[1], Louise A. Carolan[4], Teagan A. Guarnaccia[4], Anne Kelso[4], Ian G. Barr[4], Jodie McVernon[1,5], Karen L. Laurie[4], and James M. McCaw*[1,5,6]

[1]Centre for Epidemiology and Biostatistics, Melbourne School of Population and Global Health, The University of Melbourne, Melbourne, Australia.
[2]Modelling Infection and Immunity Lab, Centre for Disease Modelling, York Institute for Health Research, York University, Toronto, Ontario, Canada
[3]Mathematics and Statistics, York University, Toronto, Ontario, Canada
[4]WHO Collaborating Centre for Reference and Research on Influenza at the Peter Doherty Institute for Infection and Immunity, Parkville, Victoria, Australia.
[5]Modelling and Simulation, Infection and Immunity Theme, Murdoch Childrens Research Institute, The Royal Children's Hospital, Parkville, Victoria, Australia.
[6]School of Mathematics and Statistics, The University of Melbourne, Melbourne, Australia

*Correspondence: jamesm@unimelb.edu.au

Stochastic simulation of the re-exposure models

The conversion of the deterministic models to stochastic models starts from changing the unit of V_2 to number of particles. Here we present the stochastic model derived from Model R1, and the other two models can be constructed in the same way. The steps we take are:

- The number of the second virions (V_{2num}); we use $V_{2num} = V_2\beta_2'/\beta_2$ to convert the unit of V_2 to the unit of T.
- Since antibodies specific to the second virus have almost not been produced in the early stage of the second infection, we ignore the equations for B_2 and A_2.
- We introduce the numbers of the first virus (V_1) and the cells (T, R and I_1) remain very large in the early stage of the second infection, so they are all treated as continuous variables and solved using the Matlab ODE solver, *ode15s*.
- We also assume the immune variables (F, B_1 and A_1) are all continuous and thus are also solved by *ode15s*.

Finally, the model derived from Model R1 for stochastic simulation takes the form:

$$\frac{dT}{dt} = g(T+R)(1-\frac{T+R+I_1+I_2}{C_t}) - \beta_1' V_1 T - \beta_2 V_{2num} T + \rho R - \phi F T, \tag{S1}$$

$$\frac{dR}{dt} = \phi F T - \rho R - \xi R, \tag{S2}$$

$$\frac{dF}{dt} = q_1 I_1 + q_2 I_2 - dF, \tag{S3}$$

$$\frac{dV_1}{dt} = p_1 I_1 - c_1 V_1 - \mu_1 V_1 A_1 - \beta_1 V_1 T, \tag{S4}$$

$$\frac{dI_1}{dt} = \beta_1' V_1 T - \delta_1 I_1, \tag{S5}$$

$$\frac{dB_1}{dt} = m_{11} V_1 (1-B_1) - m_{21} B_1, \tag{S6}$$

$$\frac{dA_1}{dt} = m_{31} B_1 - r_1 A_1 - \mu_1' V_1 A_1, \tag{S6}$$

$$\frac{dV_{2num}}{dt} = \frac{p_2 I_2 \beta_2'}{\beta_2} - c_2 V_{2num} - \beta_2 V_{2num} T, \tag{S7}$$

$$\frac{dI_2}{dt} = \beta_2 V_{2num} T - \delta_2 I_2, \tag{S8}$$

where the first seven equations form the deterministic portion of the model and the last two give stochastic portion. To solve the model, we choose a time step size of $dt = 0.01$ day, which is significantly smaller than the time constants of any state transitions (e.g. viral decay or binding to target cells). We start the stochastic simulation from the time of the second inoculation, and the initial conditions are the values of the first seven variables 3 days after the first inoculation, $I_2 = 0$ and a given value of V_{2num}. For each iteration from t_0 to $t_0 + dt$, the first seven variables are solved deterministically using *ode15s* and the last two are solved stochastically using the Gillespie algorithm [1]. Updates are continued in a way that the values of V_{2num} and I_2 at time t_0 will be used as parameters in the first equations to solve the first seven variables at time $t_0 + dt$, and the value of T at time t_0 is also used to calculate V_{2num} and I_2 at time $t_0 + dt$. Matlab code

is provided at the end of this *Supporting Information*. Results are given and discussed in the main text.

Notably, we assumed that viral production from infected cells was continuous, i.e. an infected cell continuously produces virions at a rate of $p_2 I_2 \beta_2'/\beta_2$ (called the continuous model). However, a different mechanism, in which virions are only released once an infected cell dies (called the burst model), has also been proposed [2]. Although it was found that the burst model gave a shorter average extinction time than the continuous model [2], neither model violates the rule that small numbers of virions are more likely to induce stochastic extinction. Thus, in terms of our focus, we will not further elaborate on this issue in this paper but leave a detailed quantitative study for future work.

References

[1] Gillespie DT (1977) Exact stochastic simulation of coupled chemical reactions. J Phys Chem 81(25): 2340–2361.

[2] Pearson JE, Krapivsky P, Perelson AS (2011) Stochastic theory of early viral infection: continuous versus burst production of virions. PLoS Comput Biol 7(2): e1001058. doi:10.1371/journal.pcbi.1001058

Matlab code for stochastic simulations

```
% For stochastic simulations using Model R1 (see the SI for details)
clear all;clc

% initial conditions
V1_init=2.18e+5; % initial value of V1
V2_init=700; % initial value of V2
T_init=7.78e+6; % initial value of T
R_init=2.68e+7; % initial value of R
I1_init=1.55e+7; % initial value of I1
I2_init=0; % initial value of I2
F_init=22.29; % initial value of F
B1_init=0.998; % initial value of B1
A1_init=1.37; % initial value of A1

% initial conditions for the control case in Fig.12
% V1_init=0;
% V2_init=40;
% T_init=7e+7;
% R_init=0;
% I1_init=0;
% I2_init=0;
% F_init=0;
% B1_init=0;
% A1_init=0;

% the following variables record the values of corresponding
% variables at previous time point for the next iteration.
V1=V1_init;
T=T_init;
R=R_init;
I1=I1_init;
F=F_init;
B1=B1_init;
A1=A1_init;

dt=0.01; % time step size for each iteration

% parameter values (see Table 1 in the main text)
% note that s1=s2=0 and kappa1=kappa2=0
phi=0.14;rho=0.05;d=2;Ct=7e+7;g=0.8;xi=0.1;

q1=5e-6;p1=0.35;beta11=5e-7;beta21=2e-5; % beta11 is beta1 and beta21 is beta'1
s1=0;kappa1=0;c1=20.0;delta1=3;
mu11=0.2;mu21=0.04; % mu11 is mu1 and mu21 is mu'1
r1=0.2;m11=1e-4;m21=0.01;m31=12000;

q2=5e-6;p2=0.35;beta12=5e-7;beta22=2e-5; % beta12 is beta2 and beta22 is beta'2
s2=0;kappa2=0;c2=20.0;delta2=3;

V2_state=2*ones(1,V2_init); % 2 indicates live, 1 means binding to target cells, 0 is
natrual death
gv=zeros(1,V2_init); % tracking variable for V2
rv1=rand(1,V2_init); % random variable for V2 to determine the transition probability

V2num=V2_init; % V2num records the time series of free V2 numbers

I2_state=ones(1,I2_init); % state of I2; 1 indicates live, 0 is natural death
gi=zeros(1,I2_init); % tracking variable for I2
ri1=rand(1,I2_init); % random variable for I2 to determine the transition probability

I2num=I2_init; % I2num records the time series of I2 numbers

time=0;

max=1000;

wb=waitbar(0,'please wait');

for iter=1:max
    % when V2 reaches 10000, we assume it is a success event and stop it
```

```
    if V2num(end)>10000
        break
    end
    if ~isempty(V2_state)
        for i=1:length(V2_state)
            tran_sum=c2+beta12*T;
            gv(i)=gv(i)+tran_sum*dt;
            if gv(i)>=log(1/rv1(i)) % transition occurs for V2
                rv2=rand;
                if c2/tran_sum<rv2
                    V2_state(i)=1; % binding to target cells
                else
                    V2_state(i)=0; % natural death
                end
            end
        end
    end

    NewV2=round(I2num(end)*p2/(1+s2*F)*beta22/beta12*dt); % number of newly produced
V2
    NewI2=length(V2_state(V2_state == 1)); % number of newly infected cells

    gv=[gv(V2_state>1.5),zeros(1,NewV2)]; % update tracking variable for V2
    rv1=[rv1(V2_state>1.5),rand(1,NewV2)]; % update random variable for V2
    V2_state=[V2_state(V2_state > 1.5),2*ones(1,NewV2)]; % update states of all the
virions for V2

    V2num=[V2num,length(V2_state)]; % update the number of V2

    if ~isempty(I2_state)
        for i=1:length(I2_state)
            tran_sum=delta2+kappa2*F;
            gi(i)=gi(i)+tran_sum*dt;
            if gi(i)>=log(1/ri1(i)) % transition occurs for I2
                I2_state(i)=0; % death
            end
        end
    end

    gi=[gi(I2_state>0.5),zeros(1,NewI2)]; % update tracking variable for I2
    ri1=[ri1(I2_state>0.5),rand(1,NewI2)]; % update random variable for I2
    I2_state=[I2_state(I2_state > 0.5),ones(1,NewI2)]; % update states of I2

    I2num=[I2num,length(I2_state)]; % update the number of I2

    % here use ode15s to update other variables
    Y0=[V1,T,I1,R,F,B1,A1]';

    options = odeset('RelTol',1e-12,'AbsTol',1e-12);
    [Tnew,Ynew] = ode15s(@ODEmodel_part,[0 dt],Y0,options,V2num(end-1),I2num(end-1));


V1=Ynew(end,1);T=Ynew(end,2);I1=Ynew(end,3);R=Ynew(end,4);F=Ynew(end,5);B1=Ynew(end,6)
;A1=Ynew(end,7);

    time=[time,iter*dt]; % update time vector
    waitbar(iter/max)

end

close(wb)
```

The function ‘ODEmodel_part’ appearing in the above code is provided below

---

```
function ynew=ODEmodel_part(~,y,V2num,I2num)

% y=[V1,T,I1,R,F,B1,A1]

% parameter values
```

```
phi=0.14;rho=0.05;d=2;
D=(7e+7-y(2)-y(3)-y(4)-I2num)/7e+7;
g=0.8;
xi=0.1;

q1=5e-6;p1=0.35;beta11=5e-7;beta21=2e-5;s1=0;kappa1=0;c1=20.0;delta1=3;
mu11=0.2;mu21=0.04;r1=0.2;m11=1e-4;m21=0.01;m31=12000;

q2=5e-6;beta12=5e-7;

ynew=zeros(7,1);
ynew(1)=p1/(1+s1*y(5))*y(3)-c1*y(1)-r1*y(1)*y(7)-beta11*y(1)*y(2);
ynew(2)=g*D*(y(2)+y(4))-beta21*y(1)*y(2)-beta12*V2num*y(2)+rho*y(4)-phi*y(2)*y(5);
ynew(3)=beta21*y(1)*y(2)-delta1*y(3)-kappa1*y(3)*y(5);
ynew(4)=phi*y(2)*y(5)-rho*y(4)-xi*y(4);
ynew(5)=(q1*y(3)+q2*I2num)-d*y(5);
ynew(6)=m11*y(1)*(1-y(6))-m21*y(6);
ynew(7)=mu11*m31/r1*y(6)-mu21*y(1)*y(7)-r1*y(7);
```
