# Supplementary material for: Innate Immunity and the Inter-exposure Interval Determine the Dynamics of Secondary Influenza Virus Infection and Explain Observed Viral Hierarchies
Source: PLoS Comput Biol. 2015 Aug 18;11(8):e1004334. doi: 10.1371/journal.pcbi.1004334 (PMC4540579; doi:10.1371/journal.pcbi.1004334)
Supplement: S2 Text — (PDF) [file pcbi.1004334.s002.pdf]

Matlab code for deterministic simulations and generating figures shown in the main text

1. Code for generating solutions to Model 1 – 3 (e.g. Fig. 5, S1-S4)

```
clear all
clc
format long

S0=[1,7e+7,0,0,0,0,0];

options = odeset('RelTol',1e-12,'AbsTol',1e-12);
[T,Y] = ode15s(@ODEmodels,[0 14],S0,options);

%% plot solution

figure(1)
set(gcf,'Position',[10 200 500 800])
subplot(4,1,1);plot(T,log10(Y(:,1)),'-k');hold on
ylabel('log_{10}(V)','fontsize',16)
set(gca,'FontSize',15)
axis([0 14 -1 7])

subplot(4,1,2);h21=plot(T,(Y(:,2)),'-b');hold on;h22=plot(T,(Y(:,4)),'-
g');h23=plot(T,(Y(:,3)),'-r');
ylabel('No. of cells','fontsize',16)
set(gca,'FontSize',15)
hl=legend([h21,h23],'T','I');
set(hl,'FontSize',13)
axis([0 14 0 8e+7])

subplot(4,1,3);plot(T,Y(:,5),'-k');
ylabel('F','fontsize',16)
set(gca,'FontSize',15)
axis([0 14 0 40])

subplot(4,1,4);plot(T,Y(:,7),'-k');
ylabel('A','fontsize',16)
set(gca,'FontSize',15)
xlabel('time (day)','fontsize',16)
axis([0 14 0 5e+4])
```

The function "ODEmodels" is given as follows,

```
function ynew=ODEmodels(~,y)

% V: viral load
% T: target cell
% I: infected cell
% R: Resistant cell
% F: IFN
% B: activated B cells
% A: Antibody
% y=[V,T,I,R,F,B,A]

% % PARAMETERS FOR RESISTANCE CONTROL (Model 1)
% q=1e-7;
% p=0.35;
% beta1=5e-7;
% beta2=2e-5;
% kappa=0;c=20.0;d=2;delta=3;
% phi=0.14;rho=0.05;gamma=0.8;xi=0.1;
% mu1=0.2;mu2=0.04;r=0.2;
% m1=1e-4;m2=0.01;m3=12000;

% PARAMETERS FOR VIRAL PRODUCTION CONTROL (Model 2)
q=1e-7;
p=0.35/(1+1*y(5));
beta1=5e-7;beta2=2e-5;
```

```
kappa=0;c=20.0;d=2;delta=3;
phi=0;gamma=0.8;rho=0;xi=0;
mu1=0.2;mu2=0.04;r=0.2;
m1=1e-4;m2=0.01;m3=12000;

% % PARAMETERS FOR NK CONTROL (Model 3)
% q=1e-7;
% p=0.35;
% beta1=5e-7;beta2=2e-5;
% kappa=3;c=20.0;d=2;delta=3;
% phi=0;gamma=0.8;rho=0;xi=0;
% mu1=0.2;mu2=0.04;r=0.2;
% m1=1e-4;m2=0.01;m3=12000;

D=(7e+7-y(2)-y(3)-y(4))/7e+7;

ynew=zeros(7,1);
ynew(1)=p*y(3)-c*y(1)-mu1*y(1)*y(7)-beta1*y(1)*y(2);
ynew(2)=gamma*(y(2)+y(4))*D-beta2*y(1)*y(2)+rho*y(4)-phi*y(2)*y(5);
ynew(3)=beta2*y(1)*y(2)-delta*y(3)-kappa*y(3)*y(5);
ynew(4)=phi*y(2)*y(5)-rho*y(4)-xi*y(4);
ynew(5)=q*y(3)-d*y(5);
ynew(6)=m1*y(1)*(1-y(6))-m2*y(6);
ynew(7)=m3*y(6)-mu2*y(1)*y(7)-r*y(7);
```

2. Code for generating Fig. 6

```
%% Model 1
clear all
clc
format long

q=[0,10.^[-8:0.2:-4]];
phi=[0,10.^[-3:0.4:1]];
rho=[0,10.^[-3:0.5:2]];

Tagmin=zeros(length(q),length(phi),length(rho));

wb=waitbar(0,'please wait...');

for i=1:length(q)
    for j=1:length(phi)
        for k=1:length(rho)

            S0=[1,7e+7,0,0,0,0,0];

            options = odeset('RelTol',1e-12,'AbsTol',1e-12);
            [T,Y] = ode15s(@ODEmodels_sen_analysis_model1,[0 7],...
S0,options,q(i),phi(j),rho(k));

            Tagmin(i,j,k)=min(Y(:,2));

        end
    end
    waitbar(i/length(q))
end

close(wb)

save('varying_q_phi_rho_for_depletion_test_model1')

%% model 1 (fixing phi=0.14, rho=0.05)
clear all
clc
format long

q=[0,10.^[-8:0.2:-4]];

Tagmin1=zeros(1,length(q));

wb=waitbar(0,'please wait...');

for i=1:length(q)

            S0=[1,7e+7,0,0,0,0,0];

            options = odeset('RelTol',1e-12,'AbsTol',1e-12);
            [T,Y] = ode15s(@ODEmodels_sen_analysis_model1,[0 7],...
S0,options,q(i),0.14,0.05);

            Tagmin1(i)=min(Y(:,2));

    waitbar(i/length(q))
end

close(wb)

save('varying_q_for_depletion_test_model1')

%% Model 2
clear all
clc
format long

q=[0,10.^[-8:0.2:-4]];

Tagmin2=zeros(1,length(q));

wb=waitbar(0,'please wait...');
```

```
for i=1:length(q)

            S0=[1,7e+7,0,0,0,0,0];

            options = odeset('RelTol',1e-12,'AbsTol',1e-12);
            [T,Y] = ode15s(@ODEmodels_sen_analysis_model2,[0 7],...
S0,options,q(i));

            Tagmin2(i)=min(Y(:,2));

    waitbar(i/length(q))
end

close(wb)

save('varying_q_for_depletion_test_model2')

%% Model 3
clear all
clc
format long

q=[0,10.^[-8:0.2:-4]];

Tagmin3=zeros(1,length(q));

wb=waitbar(0,'please wait...');

for i=1:length(q)

            S0=[1,7e+7,0,0,0,0,0];

            options = odeset('RelTol',1e-12,'AbsTol',1e-12);
            [T,Y] = ode15s(@ODEmodels_sen_analysis_model3,[0 7],...
S0,options,q(i));

            Tagmin3(i)=min(Y(:,2));

    waitbar(i/length(q))
end

close(wb)

save('varying_q_for_depletion_test_model3')

%%
load varying_q_phi_rho_for_depletion_test_model1.mat
load varying_q_for_depletion_test_model1.mat
load varying_q_for_depletion_test_model2.mat
load varying_q_for_depletion_test_model3.mat

Target_model1=[max(max(max(Tagmin))), mean(mean(mean(Tagmin))),
min(min(min(Tagmin)))]/7e+7; % high,mean,low fraction of total 7e+7 cells

h2=semilogx(q,Tagmin2/7e+7,'.-g','linewidth',2,'markersize',30);
hold on
h3=semilogx(q,Tagmin3/7e+7,'.-r','linewidth',2,'markersize',30);
h1=semilogx(q,Tagmin1/7e+7,'.-k','linewidth',2,'markersize',30);
hold on
plot([1e-8 1e-4],[Target_model1(1),Target_model1(1)],'-k')
plot([1e-8 1e-4],[Target_model1(3),Target_model1(3)],'-k')

set(gca,'FontSize',15)
ylabel('minimum of target cell number (normalised to total 7 \times 10^7
cells)','fontsize',16)
xlabel('q','fontsize',16)
legend([h1,h2,h3],'Model 1','Model 2','Model 3')
```

The function "ODEmodels_sen_analysis_model1" is given as follows,

```
function ynew=ODEmodels_sen_analysis_model1(~,y,q,phi,rho)

% V: viral load
```

```
% T: target cell
% I: infected cell
% R: Resistant cell
% F: IFN
% B: activated B cells
% A: Antibody
% y=[V,T,I,R,F,B,A]

p=0.35;
beta1=5e-7;
beta2=2e-5;
kappa=0;c=20.0;d=2;delta=3;
gamma=0.8;xi=0.1;
mu1=0.2;mu2=0.04;r=0.2;
m1=1e-4;m2=0.01;m3=12000;

D=(7e+7-y(2)-y(3)-y(4))/7e+7;

ynew=zeros(7,1);
ynew(1)=p*y(3)-c*y(1)-mu1*y(1)*y(7)-beta1*y(1)*y(2);
ynew(2)=gamma*(y(2)+y(4))*D-beta2*y(1)*y(2)+rho*y(4)-phi*y(2)*y(5);
ynew(3)=beta2*y(1)*y(2)-delta*y(3)-kappa*y(3)*y(5);
ynew(4)=phi*y(2)*y(5)-rho*y(4)-xi*y(4);
ynew(5)=q*y(3)-d*y(5);
ynew(6)=m1*y(1)*(1-y(6))-m2*y(6);
ynew(7)=m3*y(6)-mu2*y(1)*y(7)-r*y(7);
```

The function "ODEmodels_sen_analysis_model2" is given as follows,

```
function ynew=ODEmodels_sen_analysis_model2(~,y,q)

% V: viral load
% T: target cell
% I: infected cell
% R: Resistant cell
% F: IFN
% B: activated B cells
% A: Antibody
% y=[V,T,I,R,F,B,A]


% PARAMETERS FOR VIRAL PRODUCTION CONTROL
p=0.35/(1+1*y(5));
beta1=5e-7;beta2=2e-5;
kappa=0;c1=20.0;d=2;delta=3;
phi=0;gamma=0.8;rho=0;xi=0.1;mu1=0.2;mu2=0.04;
r=0.2;m1=1e-4;m2=0.01;m3=12000;

D=(7e+7-y(2)-y(3)-y(4))/7e+7;

ynew=zeros(7,1);
ynew(1)=p*y(3)-c*y(1)-mu1*y(1)*y(7)-beta1*y(1)*y(2);
ynew(2)=gamma*(y(2)+y(4))*D-beta2*y(1)*y(2)+rho*y(4)-phi*y(2)*y(5);
ynew(3)=beta2*y(1)*y(2)-delta*y(3)-kappa*y(3)*y(5);
ynew(4)=phi*y(2)*y(5)-rho*y(4)-xi*y(4);
ynew(5)=q*y(3)-d*y(5);
ynew(6)=m1*y(1)*(1-y(6))-m2*y(6);
ynew(7)=m3*y(6)-mu2*y(1)*y(7)-r*y(7);
```

The function "ODEmodels_sen_analysis_model3" is given as follows,

```
function ynew=ODEmodels_sen_analysis_model3(~,y,q)

% V: viral load
% T: target cell
% I: infected cell
% R: Resistant cell
% F: IFN
% B: activated B cells
% A: Antibody
% y=[V,T,I,R,F,B,A]

p=0.35;
```

```
beta1=5e-7;beta2=2e-5;
kappa=3;c=20.0;d=2;delta=3;
phi=0;gamma=0.8;rho=0;xi=0;
mu1=0.2;mu2=0.04;r=0.2;
m1=1e-4;m2=0.01;m3=12000;

D=(7e+7-y(2)-y(3)-y(4))/7e+7;

ynew=zeros(7,1);
ynew(1)=p*y(3)-c*y(1)-mu1*y(1)*y(7)-beta1*y(1)*y(2);
ynew(2)=gamma*(y(2)+y(4))*D-beta2*y(1)*y(2)+rho*y(4)-phi*y(2)*y(5);
ynew(3)=beta2*y(1)*y(2)-delta*y(3)-kappa*y(3)*y(5);
ynew(4)=phi*y(2)*y(5)-rho*y(4)-xi*y(4);
ynew(5)=q*y(3)-d*y(5);
ynew(6)=m1*y(1)*(1-y(6))-m2*y(6);
ynew(7)=m3*y(6)-mu2*y(1)*y(7)-r*y(7);
```

3. Code for generating solutions to Model R1 – R3 (e.g. Fig. 7, S8-S9)

```
clear all
clc
format long

reinf_time=2; % IEI

S0=[1,7e+7,0,0,0,0,0,0,0,0,0];

options = odeset('RelTol',1e-12,'AbsTol',1e-12);
[T1,Y1] = ode15s(@ODEmodel_reinfection,[0 reinf_time],S0,options);

S1=Y1(end,:)+[0,0,0,0,0,0,0,1,0,0,0];

options = odeset('RelTol',1e-12,'AbsTol',1e-12);
[T2,Y2] = ode15s(@ODEmodel_reinfection,[reinf_time:0.01:reinf_time+40],S1,options);

T=[T1;T2];
Y=[Y1;Y2];

%% MC coefficient
figure
window=20;
cf=zeros(1,length(T2)-window+1);
for i=1:length(T2)-window+1
    cm=corrcoef(Y2(i:i+window-1,1),Y2(i:i+window-1,8));
    cf(i)=cm(1,2);
end
plot(12+T2(1:length(T2)-window+1),cf,'-ok')

%% plot solution
Ts=14-reinf_time;

figure(2)
set(gcf,'Position',[10 200 600 800])
subplot(4,1,1);plot(T+Ts,log10(Y(:,1)),'-k');hold on;plot(T+Ts,log10(Y(:,8)),'-r');
ylabel('log_{10}(V)','fontsize',16)
set(gca,'FontSize',15)
axis([0 24 -2 6])
hold on;
line([14,14],[-5,16],'LineStyle','--','Color','k')
line([0,300],[2,2],'LineStyle','--','Color','r')

subplot(4,1,2);h21=plot(T+Ts,Y(:,2),'-b');hold on;h22=plot(T+Ts,Y(:,4),'-g');
ylabel('No. of cells','fontsize',16)
set(gca,'FontSize',15)
% legend([h21,h22],'T','R')
axis([0 24 0 1e+8])
hold on;line([14,14],[0,1e+8],'LineStyle','--','Color','k')

subplot(4,1,3);plot(T+Ts,Y(:,5),'-k');
ylabel('F','fontsize',16)
set(gca,'FontSize',15)
axis([0 24 0 10])
hold on;line([14,14],[0,50],'LineStyle','--','Color','k')

subplot(4,1,4);plot(T+Ts,(Y(:,7)),'-k');hold on;plot(T+Ts,(Y(:,11)),'-r');
ylabel('A','fontsize',16)
set(gca,'FontSize',15)
axis([0 24 -2 4e+4])
hold on;
line([14,14],[-2,4e+4],'LineStyle','--','Color','k')

xlabel('time (day)','fontsize',16)
```

The function "ODEmodel_reinfection" is given as follows,

```
function ynew=ODEmodel_reinfection(~,y)

% V: viral load
% T: target cell
% I: infected cell
```

```matlab
% R: Resistant cell
% F: IFN
% B: activated B cells
% A: Antibody
% y=[V1,T,I1,R,F,B1,A1,V2,I2,B2,A2]

% parameters for Model R1

% phi=0.14;rho=0.05;d=2;
% D=(7e+7-y(2)-y(3)-y(4)-y(9))/7e+7;g=0.8;xi=0.1;
%
% q1=5e-6;
% p1=0.35;beta11=5e-7;beta21=2e-5;kappa1=0;c1=20.0;
% delta1=3;
% mu11=0.2;
% mu21=0.04;
% r1=0.2;
% m11=1e-4;
% m21=0.01;
% m31=12000;
%
% q2=1e-7;
% p2=0.35;beta12=5e-7;beta22=2e-5;kappa2=0;c2=20.0;
% delta2=3;mu12=0.2;mu22=0.04;
% r2=0.2;m12=1e-4;m22=0.01;m32=12000;

% parameters for Model R2

% q1=1e-8;
% phi=0;rho=0;d=2;
% D=(7e+7-y(2)-y(3)-y(4)-y(9))/7e+7;g=0.8;xi=0.1;
% p1=0.35/(1+1*y(5));beta11=5e-7;beta21=2e-5;
% kappa1=0;c1=20.0;delta1=3;mu11=0.2;mu21=0.04;
% r1=0.2;m11=1e-4;m21=0.01;m31=12000;

% q2=1e-8;
% p2=0.35/(1+1*y(5));beta12=5e-7;beta22=2e-5;
% kappa2=0;c2=20.0;delta2=3;mu12=0.2;mu22=0.04;
% r2=0.2;m12=1e-4;m22=0.01;m32=12000;

% parameters for Model R3

phi=0;rho=0;d=2;
D=(7e+7-y(2)-y(3)-y(4)-y(9))/7e+7;
g=0.8;xi=0.1;

q1=5e-6;
p1=0.35;beta11=5e-7;beta21=2e-5;kappa1=3;c1=20.0;
delta1=3;mu11=0.2;mu21=0.04;r1=0.2;
m11=1e-4;m21=0.01;m31=12000;

q2=1e-7;
p2=0.35;beta12=5e-7;beta22=2e-5;
kappa2=3;c2=20.0;delta2=3;
mu12=0.2;mu22=0.04;r2=0.2;
m12=1e-4;m22=0.01;m32=12000;

ynew=zeros(11,1);
ynew(1)=p1*y(3)-c1*y(1)-mu11*y(1)*y(7)-beta11*y(1)*y(2);
ynew(2)=g*D*(y(2)+y(4))-beta21*y(1)*y(2)-beta22*y(8)*y(2)+rho*y(4)-phi*y(2)*y(5);
ynew(3)=beta21*y(1)*y(2)-delta1*y(3)-kappa1*y(3)*y(5);
ynew(4)=phi*y(2)*y(5)-rho*y(4)-xi*y(4);
ynew(5)=(q1*y(3)+q2*y(9))-d*y(5);
ynew(6)=m11*y(1)*(1-y(6))-m21*y(6);
ynew(7)=m31*y(6)-mu21*y(1)*y(7)-r1*y(7);
ynew(8)=p2*y(9)-c2*y(8)-mu12*y(8)*y(11)-beta12*y(8)*y(2);
ynew(9)=beta22*y(8)*y(2)-delta2*y(9)-kappa2*y(9)*y(5);
ynew(10)=m12*y(8)*(1-y(10))-m22*y(10);
ynew(11)=m32*y(10)-mu22*y(8)*y(11)-r2*y(11);
```

4. Code for generating Fig. S10 (or Fig. 9)

(Note that: there is a function called "crossing.m" used for finding zero point of a vector. A link to download it is http://au.mathworks.com/matlabcentral/fileexchange/2432-crossing. **You can also easily find it using Google search.)**

```
% Using this code to generate the data for color figures
clear all;clc
% format long
%
% reinf_time=3;
%
% S0=[1,7e+7,0,0,0,0,0,0,0,0,0];
% options = odeset('RelTol',1e-12,'AbsTol',1e-12);
% [T1,Y1] = ode15s(@ODEmodel_reinfection,[0:0.01:reinf_time],S0,options);
% S1=Y1(end,:)+[0,0,0,0,0,0,0,1,0,0,0];
% options = odeset('RelTol',1e-12,'AbsTol',1e-12);
% [T2,Y2] = ode15s(@ODEmodel_reinfection,[reinf_time:0.01:reinf_time+50],S1,options);
%
% T=[T1;T2];
% Y=[Y1;Y2];
%
% Ts=14-reinf_time;
%
% figure
% set(gcf,'Position',[10 200 500 600])
% subplot(3,1,1);h11=plot(T+Ts,log10(Y(:,1)),'-k');hold
on;h12=plot(T+Ts,log10(Y(:,8)),'-r');
% ylabel('log_{10}(V)','fontsize',16)
% set(gca,'FontSize',15)
% % legend([h11,h12],'V1','V2')
% axis([0 24 -2 6])
% hold on;
% line([14,14],[-5,16],'LineStyle','--','Color','k')
% % line([0,300],[2,2],'LineStyle','--','Color','r')
% text(5,3,['interval = ',num2str(reinf_time),' days'],'fontsize',15)
%
% subplot(3,1,2);h21=plot(T+Ts,Y(:,2),'-b');hold on;h22=plot(T+Ts,Y(:,3),'-r');
% ylabel('No. of cells','fontsize',16)
% set(gca,'FontSize',15)
% % legend([h21,h22],'T','I')
% axis([0 24 0 1e+8])
% hold on;line([14,14],[0,1e+8],'LineStyle','--','Color','k')
%
% subplot(3,1,3);
% window=20;
% cf=zeros(1,length(T2)-window+1);
% for i=1:length(T2)-window+1
%     cm=corrcoef(Y2(i:i+window-1,1),Y2(i:i+window-1,8));
%     cf(i)=cm(1,2);
% end
% plot(Ts+0.01*window/2+T2(1:(length(T2)-window+1)),cf,'-ok')
% axis([0 24 -1 1])
% set(gca,'FontSize',15)
% ylabel('MC coefficient','fontsize',16)
% hold on;line([14,14],[-10,1e+8],'LineStyle','--','Color','k')
% line([0,300],[0,0],'LineStyle','--','Color','k')
%
% xlabel('time (day)','fontsize',16)
%
% % find phase-transition point using MC coefficient
% timeuse=Ts+0.01*window/2+T2(1:(length(T2)-window+1));
% ind=crossing(cf);
% indt=timeuse(ind);
% indt(1:3)
%
% % find the first peak of V2
% der=Y2(2:end,8)-Y2(1:end-1,8);
% indp=crossing(der);
% indt_peak=T2(indp)+Ts;
```

```
% indt_peak(1:2)

%% color figures (Model R1) q1=1e-8,q2=1e-8

Itv=[1:1:14];

% the data is based on both model solution and MC coefficient
% FstPeak separates Phase 1 and 2; L1 separates Phase 2 and 3; L2 separates Phase 3
and 4;
L1=[inf,21.53,19.86,18.86,17.86,16.86,15.86,14.85,14,14,14,14,14,14]-14;
L2=[NaN,29.31,27.42,26.57,24.87,23.20,21.62,20.21,19.06,18.23,17.71,17.42,17.28,17.21]
-14;
FstPeak=[16.12,15.14,14,14,14,14,14,14,14,14,14,14,14,14]-14;

figure(3)
subplot(4,4,16);
h1=plot(L1,Itv,'-k','linewidth',1);
hold on
h2=plot(L2,Itv,'-k','linewidth',1);
h3=plot(FstPeak,Itv,'-k','linewidth',1);
set(gcf,'Position',[10 200 500 400])
set(gca,'FontSize',12,'xtick',0:3:14,'ytick',1:3:10)
set(gca,'YDir','reverse')
axis([0 13 1 10])
% ylabel('inter-exposure interval (day)','fontsize',16)
xlabel('time (day)','fontsize',16)

line([-1,100],[2,2],'LineStyle','--','Color','k','linewidth',1)
line([-1,100],[3,3],'LineStyle','--','Color','k','linewidth',1)
line([-1,100],[9,9],'LineStyle','--','Color','k','linewidth',1)


indfst=sum(heaviside(FstPeak-0.01))+1;

P1x=[0,FstPeak(1:indfst),0];
P1y=[1,1:indfst,1];

indl1=sum(heaviside(L1(2:end)-0.01))+2;

P2x=[fliplr(FstPeak(1:indfst)),13,13,L1(2:indl1),0];
P2y=[indfst:-1:1,1,2,2:indl1,indfst];

P3x=[fliplr(L1(2:indl1)),L2(2:10),0,0];
P3y=[indl1:-1:2,2:10,10,indl1];

P4x=[13,13,L2(2:10),13];
P4y=[10,2,2:10,10];

hold on
patch(P1x,P1y,[0 0 0],'facealpha',0.2)
patch(P2x,P2y,[1 0 0],'facealpha',0.3)
patch(P3x,P3y,[0 1 0],'facealpha',0.3)
patch(P4x,P4y,[0 162/255 1],'facealpha',0.3)

text(8,7.75,'q_1 = 1\times 10^{-8}')
text(8,8.75,'q_2 = 1\times 10^{-8}')

%% color figures (Model R1) q1=1e-8,q2=1e-7

Itv=[1:1:14];

L1=[inf,21.53,19.86,18.86,17.86,16.86,15.86,14.85,14,14,14,14,14,14]-14;
L2=[NaN,27.87,27.41,26.57,24.87,23.20,21.62,20.21,19.06,18.23,17.71,17.42,17.28,17.21]
-14;
FstPeak=[16.12,15.14,14,14,14,14,14,14,14,14,14,14,14,14]-14;

figure(3)
subplot(4,4,15);
h1=plot(L1,Itv,'-k','linewidth',1);
hold on
h2=plot(L2,Itv,'-k','linewidth',1);
h3=plot(FstPeak,Itv,'-k','linewidth',1);
set(gcf,'Position',[10 200 500 400])
set(gca,'FontSize',12,'xtick',0:2:14,'ytick',1:3:10)
```

```
set(gca,'YDir','reverse')
axis([0 13 1 10])
% ylabel('inter-exposure interval (day)','fontsize',16)
% xlabel('time (day)','fontsize',16)

line([-1,100],[2,2],'LineStyle','--','Color','k','linewidth',1)
line([-1,100],[3,3],'LineStyle','--','Color','k','linewidth',1)
line([-1,100],[9,9],'LineStyle','--','Color','k','linewidth',1)


indfst=sum(heaviside(FstPeak-0.01))+1;

P1x=[0,FstPeak(1:indfst),0];
P1y=[1,1:indfst,1];

indl1=sum(heaviside(L1(2:end)-0.01))+2;

P2x=[fliplr(FstPeak(1:indfst)),13,13,L1(2:indl1),0];
P2y=[indfst:-1:1,1,2,2:indl1,indfst];

P3x=[fliplr(L1(2:indl1)),L2(2:10),0,0];
P3y=[indl1:-1:2,2:10,10,indl1];

P4x=[13,13,L2(2:10),13];
P4y=[10,2,2:10,10];

hold on
patch(P1x,P1y,[0 0 0],'facealpha',0.2)
patch(P2x,P2y,[1 0 0],'facealpha',0.3)
patch(P3x,P3y,[0 1 0],'facealpha',0.3)
patch(P4x,P4y,[0 162/255 1],'facealpha',0.3)

text(8,7.75,'q_1 = 1\times 10^{-8}')
text(8,8.75,'q_2 = 1\times 10^{-7}')

%% color figures (Model R1) q1=1e-8,q2=5e-6

Itv=[1:1:14];

L1=[inf,21.52,19.86,18.86,17.86,16.86,15.86,14.85,14,14,14,14,14,14]-14;
L2=[NaN,29.20,27.32,26.47,24.78,23.10,21.52,20.11,18.96,18.13,17.61,17.32,17.18,17.12]
-14;
FstPeak=[16.12,15.14,14,14,14,14,14,14,14,14,14,14,14,14]-14;

figure(3)
subplot(4,4,14);
h1=plot(L1,Itv,'-k','linewidth',1);
hold on
h2=plot(L2,Itv,'-k','linewidth',1);
h3=plot(FstPeak,Itv,'-k','linewidth',1);
set(gcf,'Position',[10 200 500 400])
set(gca,'FontSize',12,'xtick',0:2:14,'ytick',1:3:10)
set(gca,'YDir','reverse')
axis([0 13 1 10])
% ylabel('inter-exposure interval (day)','fontsize',16)
% xlabel('time (day)','fontsize',16)

line([-1,100],[2,2],'LineStyle','--','Color','k','linewidth',1)
line([-1,100],[3,3],'LineStyle','--','Color','k','linewidth',1)
line([-1,100],[9,9],'LineStyle','--','Color','k','linewidth',1)


indfst=sum(heaviside(FstPeak-0.01))+1;

P1x=[0,FstPeak(1:indfst),0];
P1y=[1,1:indfst,1];

indl1=sum(heaviside(L1(2:end)-0.01))+2;

P2x=[fliplr(FstPeak(1:indfst)),13,13,L1(2:indl1),0];
P2y=[indfst:-1:1,1,2,2:indl1,indfst];

P3x=[fliplr(L1(2:indl1)),L2(2:10),0,0];
P3y=[indl1:-1:2,2:10,10,indl1];
```

```
P4x=[13,13,L2(2:10),13];
P4y=[10,2,2:10,10];

hold on
patch(P1x,P1y,[0 0 0],'facealpha',0.2)
patch(P2x,P2y,[1 0 0],'facealpha',0.3)
patch(P3x,P3y,[0 1 0],'facealpha',0.3)
patch(P4x,P4y,[0 162/255 1],'facealpha',0.3)

text(8,7.75,'q_1 = 1\times 10^{-8}')
text(8,8.75,'q_2 = 5\times 10^{-6}')

%% color figures (Model R1) q1=1e-8,q2=5e-5

Itv=[1:1:14];

L1=[inf,21.45,19.86,18.86,17.86,16.86,15.86,14.86,14,14,14,14,14,14]-14;
L2=[NaN,28.74,27.00,26.15,24.45,22.77,21.18,19.77,18.61,17.78,17.27,16.99,16.86,16.79]
-14;
FstPeak=[16.11,15.14,14,14,14,14,14,14,14,14,14,14,14,14]-14;

figure(3)
subplot(4,4,13);
h1=plot(L1,Itv,'-k','linewidth',1);
hold on
h2=plot(L2,Itv,'-k','linewidth',1);
h3=plot(FstPeak,Itv,'-k','linewidth',1);
set(gcf,'Position',[10 200 500 400])
set(gca,'FontSize',12,'xtick',0:2:14,'ytick',1:3:10)
set(gca,'YDir','reverse')
axis([0 13 1 10])
% ylabel('inter-exposure interval (day)','fontsize',16)
% xlabel('time (day)','fontsize',16)

line([-1,100],[2,2],'LineStyle','--','Color','k','linewidth',1)
line([-1,100],[3,3],'LineStyle','--','Color','k','linewidth',1)
line([-1,100],[9,9],'LineStyle','--','Color','k','linewidth',1)


indfst=sum(heaviside(FstPeak-0.01))+1;

P1x=[0,FstPeak(1:indfst),0];
P1y=[1,1:indfst,1];

indl1=sum(heaviside(L1(2:end)-0.01))+2;

P2x=[fliplr(FstPeak(1:indfst)),13,13,L1(2:indl1),0];
P2y=[indfst:-1:1,1,2,2:indl1,indfst];

P3x=[fliplr(L1(2:indl1)),L2(2:10),0,0];
P3y=[indl1:-1:2,2:10,10,indl1];

P4x=[13,13,L2(2:10),13];
P4y=[10,2,2:10,10];

hold on
patch(P1x,P1y,[0 0 0],'facealpha',0.2)
patch(P2x,P2y,[1 0 0],'facealpha',0.3)
patch(P3x,P3y,[0 1 0],'facealpha',0.3)
patch(P4x,P4y,[0 162/255 1],'facealpha',0.3)

text(8,7.75,'q_1 = 1\times 10^{-8}')
text(8,8.75,'q_2 = 5\times 10^{-5}')


%% color figures (Model R1) q1=1e-7,q2=1e-8

Itv=[1:1:14];

L1=[inf,19.74,18.18,17.18,16.18,15.18,14,14,14,14,14,14,14,14]-14;
L2=[NaN,26.17,24.86,23.89,22.16,20.63,19.37,18.44,17.83,17.49,17.32,17.23,17.19,17.17]
-14;
FstPeak=[16.11,15.14,14,14,14,14,14,14,14,14,14,14,14,14]-14;
```

```
figure(3)
subplot(4,4,12);
h1=plot(L1,Itv,'-k','linewidth',1);
hold on
h2=plot(L2,Itv,'-k','linewidth',1);
h3=plot(FstPeak,Itv,'-k','linewidth',1);
set(gcf,'Position',[10 200 500 400])
set(gca,'FontSize',12,'xtick',0:2:14,'ytick',1:3:10)
set(gca,'YDir','reverse')
axis([0 13 1 10])
% ylabel('inter-exposure interval (day)','fontsize',16)
% xlabel('time (day)','fontsize',16)

line([-1,100],[2,2],'LineStyle','--','Color','k','linewidth',1)
line([-1,100],[3,3],'LineStyle','--','Color','k','linewidth',1)
line([-1,100],[7,7],'LineStyle','--','Color','k','linewidth',1)


indfst=sum(heaviside(FstPeak-0.01))+1;

P1x=[0,FstPeak(1:indfst),0];
P1y=[1,1:indfst,1];

indl1=sum(heaviside(L1(2:end)-0.01))+2;

P2x=[fliplr(FstPeak(1:indfst)),13,13,L1(2:indl1),0];
P2y=[indfst:-1:1,1,2,2:indl1,indfst];

P3x=[fliplr(L1(2:indl1)),L2(2:10),0,0];
P3y=[indl1:-1:2,2:10,10,indl1];

P4x=[13,13,L2(2:10),13];
P4y=[10,2,2:10,10];

hold on
patch(P1x,P1y,[0 0 0],'facealpha',0.2)
patch(P2x,P2y,[1 0 0],'facealpha',0.3)
patch(P3x,P3y,[0 1 0],'facealpha',0.3)
patch(P4x,P4y,[0 162/255 1],'facealpha',0.3)

text(8,8.5,'q_1 = 1\times 10^{-7}')
text(8,9.5,'q_2 = 1\times 10^{-8}')

%% color figures (Model R1) q1=1e-7,q2=1e-7

Itv=[1:1:14];

L1=[inf,19.74,18.18,17.18,16.18,15.18,14,14,14,14,14,14,14,14]-14;
L2=[NaN,26.17,24.86,23.88,22.16,20.63,19.37,18.43,17.83,17.49,17.32,17.23,17.20,17.18]
-14;
FstPeak=[16.11,15.14,14,14,14,14,14,14,14,14,14,14,14,14]-14;

figure(3)
subplot(4,4,11);
h1=plot(L1,Itv,'-k','linewidth',1);
hold on
h2=plot(L2,Itv,'-k','linewidth',1);
h3=plot(FstPeak,Itv,'-k','linewidth',1);
set(gcf,'Position',[10 200 500 400])
set(gca,'FontSize',12,'xtick',0:2:14,'ytick',1:3:10)
set(gca,'YDir','reverse')
axis([0 13 1 10])
% ylabel('inter-exposure interval (day)','fontsize',16)
% xlabel('time (day)','fontsize',16)

line([-1,100],[2,2],'LineStyle','--','Color','k','linewidth',1)
line([-1,100],[3,3],'LineStyle','--','Color','k','linewidth',1)
line([-1,100],[7,7],'LineStyle','--','Color','k','linewidth',1)


indfst=sum(heaviside(FstPeak-0.01))+1;

P1x=[0,FstPeak(1:indfst),0];
```

```
P1y=[1,1:indfst,1];

indl1=sum(heaviside(L1(2:end)-0.01))+2;

P2x=[fliplr(FstPeak(1:indfst)),13,13,L1(2:indl1),0];
P2y=[indfst:-1:1,1,2,2:indl1,indfst];

P3x=[fliplr(L1(2:indl1)),L2(2:10),0,0];
P3y=[indl1:-1:2,2:10,10,indl1];

P4x=[13,13,L2(2:10),13];
P4y=[10,2,2:10,10];

hold on
patch(P1x,P1y,[0 0 0],'facealpha',0.2)
patch(P2x,P2y,[1 0 0],'facealpha',0.3)
patch(P3x,P3y,[0 1 0],'facealpha',0.3)
patch(P4x,P4y,[0 162/255 1],'facealpha',0.3)

text(8,8.5,'q_1 = 1\times 10^{-7}')
text(8,9.5,'q_2 = 1\times 10^{-7}')

%% color figures (Model R1) q1=1e-7,q2=5e-6

Itv=[1:1:14];

L1=[inf,19.73,18.18,17.18,16.18,15.18,14,14,14,14,14,14,14,14]-14;
L2=[NaN,26.07,24.76,23.79,22.06,20.53,19.27,18.33,17.73,17.39,17.22,17.13,17.10,17.08]
-14;
FstPeak=[16.11,15.14,14,14,14,14,14,14,14,14,14,14,14,14]-14;

figure(3)
subplot(4,4,10);
h1=plot(L1,Itv,'-k','linewidth',1);
hold on
h2=plot(L2,Itv,'-k','linewidth',1);
h3=plot(FstPeak,Itv,'-k','linewidth',1);
set(gcf,'Position',[10 200 500 400])
set(gca,'FontSize',12,'xtick',0:2:14,'ytick',1:3:10)
set(gca,'YDir','reverse')
axis([0 13 1 10])
% ylabel('inter-exposure interval (day)','fontsize',16)
% xlabel('time (day)','fontsize',16)

line([-1,100],[2,2],'LineStyle','--','Color','k','linewidth',1)
line([-1,100],[3,3],'LineStyle','--','Color','k','linewidth',1)
line([-1,100],[7,7],'LineStyle','--','Color','k','linewidth',1)


indfst=sum(heaviside(FstPeak-0.01))+1;

P1x=[0,FstPeak(1:indfst),0];
P1y=[1,1:indfst,1];

indl1=sum(heaviside(L1(2:end)-0.01))+2;

P2x=[fliplr(FstPeak(1:indfst)),13,13,L1(2:indl1),0];
P2y=[indfst:-1:1,1,2,2:indl1,indfst];

P3x=[fliplr(L1(2:indl1)),L2(2:10),0,0];
P3y=[indl1:-1:2,2:10,10,indl1];

P4x=[13,13,L2(2:10),13];
P4y=[10,2,2:10,10];

hold on
patch(P1x,P1y,[0 0 0],'facealpha',0.2)
patch(P2x,P2y,[1 0 0],'facealpha',0.3)
patch(P3x,P3y,[0 1 0],'facealpha',0.3)
patch(P4x,P4y,[0 162/255 1],'facealpha',0.3)

text(8,8.5,'q_1 = 1\times 10^{-7}')
text(8,9.5,'q_2 = 5\times 10^{-6}')
```

```
%% color figures (Model R1) q1=1e-7,q2=5e-5

Itv=[1:1:14];

L1=[inf,19.72,18.18,17.18,16.18,15.18,14,14,14,14,14,14,14,14]-14;
L2=[NaN,25.72,24.43,23.46,21.73,20.19,18.92,17.99,17.39,17.06,16.89,16.81,16.77,16.76]
-14;
FstPeak=[16.11,15.14,14,14,14,14,14,14,14,14,14,14,14,14]-14;

figure(3)
subplot(4,4,9);
h1=plot(L1,Itv,'-k','linewidth',1);
hold on
h2=plot(L2,Itv,'-k','linewidth',1);
h3=plot(FstPeak,Itv,'-k','linewidth',1);
set(gcf,'Position',[10 200 500 400])
set(gca,'FontSize',12,'xtick',0:2:14,'ytick',1:3:10)
set(gca,'YDir','reverse')
axis([0 13 1 10])
% ylabel('inter-exposure interval (day)','fontsize',16)
% xlabel('time (day)','fontsize',16)

line([-1,100],[2,2],'LineStyle','--','Color','k','linewidth',1)
line([-1,100],[3,3],'LineStyle','--','Color','k','linewidth',1)
line([-1,100],[7,7],'LineStyle','--','Color','k','linewidth',1)


indfst=sum(heaviside(FstPeak-0.01))+1;

P1x=[0,FstPeak(1:indfst),0];
P1y=[1,1:indfst,1];

indl1=sum(heaviside(L1(2:end)-0.01))+2;

P2x=[fliplr(FstPeak(1:indfst)),13,13,L1(2:indl1),0];
P2y=[indfst:-1:1,1,2,2:indl1,indfst];

P3x=[fliplr(L1(2:indl1)),L2(2:10),0,0];
P3y=[indl1:-1:2,2:10,10,indl1];

P4x=[13,13,L2(2:10),13];
P4y=[10,2,2:10,10];

hold on
patch(P1x,P1y,[0 0 0],'facealpha',0.2)
patch(P2x,P2y,[1 0 0],'facealpha',0.3)
patch(P3x,P3y,[0 1 0],'facealpha',0.3)
patch(P4x,P4y,[0 162/255 1],'facealpha',0.3)

text(8,8.5,'q_1 = 1\times 10^{-7}')
text(8,9.5,'q_2 = 5\times 10^{-5}')

%% color figures (Model R1) q1=5e-6,q2=1e-8

Itv=[1:1:14];

L1=[inf,17.48,16.21,15.22,14,14,14,14,14,14,14,14,14,14]-14;
L2=[NaN,23.45,23.04,21.66,20.28,19.33,18.70,18.29,18.02,17.84,17.71,17.60,17.53,17.46]
-14;
FstPeak=[16.02,15.04,14,14,14,14,14,14,14,14,14,14,14,14]-14;

figure(3)
subplot(4,4,8);
h1=plot(L1,Itv,'-k','linewidth',1);
hold on
h2=plot(L2,Itv,'-k','linewidth',1);
h3=plot(FstPeak,Itv,'-k','linewidth',1);
set(gcf,'Position',[10 200 500 400])
set(gca,'FontSize',12,'xtick',0:2:14,'ytick',1:3:10)
set(gca,'YDir','reverse')
axis([0 13 1 10])
% ylabel('inter-exposure interval (day)','fontsize',16)
% xlabel('time (day)','fontsize',16)
```

```
line([-1,100],[2,2],'LineStyle','--','Color','k','linewidth',1)
line([-1,100],[3,3],'LineStyle','--','Color','k','linewidth',1)
line([-1,100],[5,5],'LineStyle','--','Color','k','linewidth',1)


indfst=sum(heaviside(FstPeak-0.01))+1;

P1x=[0,FstPeak(1:indfst),0];
P1y=[1,1:indfst,1];

indl1=sum(heaviside(L1(2:end)-0.01))+2;

P2x=[fliplr(FstPeak(1:indfst)),13,13,L1(2:indl1),0];
P2y=[indfst:-1:1,1,2,2:indl1,indfst];

P3x=[fliplr(L1(2:indl1)),L2(2:10),0,0];
P3y=[indl1:-1:2,2:10,10,indl1];

P4x=[13,13,L2(2:10),13];
P4y=[10,2,2:10,10];

hold on
patch(P1x,P1y,[0 0 0],'facealpha',0.2)
patch(P2x,P2y,[1 0 0],'facealpha',0.3)
patch(P3x,P3y,[0 1 0],'facealpha',0.3)
patch(P4x,P4y,[0 162/255 1],'facealpha',0.3)

text(8,8,'q_1 = 5\times 10^{-6}')
text(8,9,'q_2 = 1\times 10^{-8}')

%% color figures (Model R1) q1=5e-6,q2=1e-7

Itv=[1:1:14];

L1=[inf,17.48,16.22,15.2,14,14,14,14,14,14,14,14,14,14]-14;
L2=[NaN,23.45,23.04,21.65,20.28,19.33,18.70,18.29,18.02,17.84,17.70,17.60,17.52,17.46]
-14;
FstPeak=[16.02,15.04,14,14,14,14,14,14,14,14,14,14,14,14]-14;

figure(3)
subplot(4,4,7);
h1=plot(L1,Itv,'-k','linewidth',1);
hold on
h2=plot(L2,Itv,'-k','linewidth',1);
h3=plot(FstPeak,Itv,'-k','linewidth',1);
set(gcf,'Position',[10 200 500 400])
set(gca,'FontSize',12,'xtick',0:2:14,'ytick',1:3:10)
set(gca,'YDir','reverse')
axis([0 13 1 10])
% ylabel('inter-exposure interval (day)','fontsize',16)
% xlabel('time (day)','fontsize',16)

line([-1,100],[2,2],'LineStyle','--','Color','k','linewidth',1)
line([-1,100],[3,3],'LineStyle','--','Color','k','linewidth',1)
line([-1,100],[5,5],'LineStyle','--','Color','k','linewidth',1)


indfst=sum(heaviside(FstPeak-0.01))+1;

P1x=[0,FstPeak(1:indfst),0];
P1y=[1,1:indfst,1];

indl1=sum(heaviside(L1(2:end)-0.01))+2;

P2x=[fliplr(FstPeak(1:indfst)),13,13,L1(2:indl1),0];
P2y=[indfst:-1:1,1,2,2:indl1,indfst];

P3x=[fliplr(L1(2:indl1)),L2(2:10),0,0];
P3y=[indl1:-1:2,2:10,10,indl1];

P4x=[13,13,L2(2:10),13];
P4y=[10,2,2:10,10];

hold on
```

```
patch(P1x,P1y,[0 0 0],'facealpha',0.2)
patch(P2x,P2y,[1 0 0],'facealpha',0.3)
patch(P3x,P3y,[0 1 0],'facealpha',0.3)
patch(P4x,P4y,[0 162/255 1],'facealpha',0.3)

text(8,8,'q_1 = 5\times 10^{-6}')
text(8,9,'q_2 = 1\times 10^{-7}')

%% color figures (Model R1) q1=5e-6,q2=5e-6

Itv=[1:1:14];

L1=[inf,17.5,16.21,15.2,14,14,14,14,14,14,14,14,14,14]-14;
L2=[NaN,23.3,22.91,21.5,20.2,19.2,18.6,18.17,17.9,17.72,17.59,17.50,17.42,17.36]-14;
FstPeak=[16.02,15.04,14,14,14,14,14,14,14,14,14,14,14,14]-14;

figure(3)
subplot(4,4,6);
h1=plot(L1,Itv,'-k','linewidth',1);
hold on
h2=plot(L2,Itv,'-k','linewidth',1);
h3=plot(FstPeak,Itv,'-k','linewidth',1);
set(gcf,'Position',[10 200 500 400])
set(gca,'FontSize',12,'xtick',0:2:14,'ytick',1:3:10)
set(gca,'YDir','reverse')
axis([0 13 1 10])
% ylabel('inter-exposure interval (day)','fontsize',16)
% xlabel('time (day)','fontsize',16)

line([-1,100],[2,2],'LineStyle','--','Color','k','linewidth',1)
line([-1,100],[3,3],'LineStyle','--','Color','k','linewidth',1)
line([-1,100],[5,5],'LineStyle','--','Color','k','linewidth',1)

indfst=sum(heaviside(FstPeak-0.01))+1;

P1x=[0,FstPeak(1:indfst),0];
P1y=[1,1:indfst,1];

indl1=sum(heaviside(L1(2:end)-0.01))+2;

P2x=[fliplr(FstPeak(1:indfst)),13,13,L1(2:indl1),0];
P2y=[indfst:-1:1,1,2,2:indl1,indfst];

P3x=[fliplr(L1(2:indl1)),L2(2:10),0,0];
P3y=[indl1:-1:2,2:10,10,indl1];

P4x=[13,13,L2(2:10),13];
P4y=[10,2,2:10,10];

hold on
patch(P1x,P1y,[0 0 0],'facealpha',0.2)
patch(P2x,P2y,[1 0 0],'facealpha',0.3)
patch(P3x,P3y,[0 1 0],'facealpha',0.3)
patch(P4x,P4y,[0 162/255 1],'facealpha',0.3)

text(8,8,'q_1 = 5\times 10^{-6}')
text(8,9,'q_2 = 5\times 10^{-6}')

%% color figures (Model R1) q1=5e-6,q2=5e-5

Itv=[1:1:14];

L1=[inf,17.48,16.22,15.21,14,14,14,14,14,14,14,14,14,14]-14;
L2=[NaN,22.86,22.45,21.07,19.68,18.73,18.12,17.73,17.49,17.32,17.21,17.12,17.05,17.00]
-14;
FstPeak=[16.01,15.03,14,14,14,14,14,14,14,14,14,14,14,14]-14;

figure(3)
subplot(4,4,5);
h1=plot(L1,Itv,'-k','linewidth',1);
hold on
h2=plot(L2,Itv,'-k','linewidth',1);
h3=plot(FstPeak,Itv,'-k','linewidth',1);
set(gcf,'Position',[10 200 500 400])
```

```
set(gca,'FontSize',12,'xtick',0:2:14,'ytick',1:3:10)
set(gca,'YDir','reverse')
axis([0 13 1 10])
% ylabel('inter-exposure interval (day)','fontsize',16)
% xlabel('time (day)','fontsize',16)

line([-1,100],[2,2],'LineStyle','--','Color','k','linewidth',1)
line([-1,100],[3,3],'LineStyle','--','Color','k','linewidth',1)
line([-1,100],[5,5],'LineStyle','--','Color','k','linewidth',1)


indfst=sum(heaviside(FstPeak-0.01))+1;

P1x=[0,FstPeak(1:indfst),0];
P1y=[1,1:indfst,1];

indl1=sum(heaviside(L1(2:end)-0.01))+2;

P2x=[fliplr(FstPeak(1:indfst)),13,13,L1(2:indl1),0];
P2y=[indfst:-1:1,1,2,2:indl1,indfst];

P3x=[fliplr(L1(2:indl1)),L2(2:10),0,0];
P3y=[indl1:-1:2,2:10,10,indl1];

P4x=[13,13,L2(2:10),13];
P4y=[10,2,2:10,10];

hold on
patch(P1x,P1y,[0 0 0],'facealpha',0.2)
patch(P2x,P2y,[1 0 0],'facealpha',0.3)
patch(P3x,P3y,[0 1 0],'facealpha',0.3)
patch(P4x,P4y,[0 162/255 1],'facealpha',0.3)

text(8,8,'q_1 = 5\times 10^{-6}')
text(8,9,'q_2 = 5\times 10^{-5}')

%% color figures (Model R1) q1=5e-5,q2=1e-8

Itv=[1:1:14];

L1=[inf,17.72,16.66,15.66,14.66,14,14,14,14,14,14,14,14,14]-14;
L2=[NaN,24.69,24.43,22.73,21.22,20.11,19.33,18.79,18.42,18.14,17.94,17.79,17.67,17.58]
-14;
FstPeak=[15.71,14.74,14,14,14,14,14,14,14,14,14,14,14,14]-14;

figure(3)
subplot(4,4,4);
h1=plot(L1,Itv,'-k','linewidth',1);
hold on
h2=plot(L2,Itv,'-k','linewidth',1);
h3=plot(FstPeak,Itv,'-k','linewidth',1);
set(gcf,'Position',[10 200 500 400])
set(gca,'FontSize',12,'xtick',0:2:14,'ytick',1:3:10)
set(gca,'YDir','reverse')
axis([0 13 1 10])
% ylabel('inter-exposure interval (day)','fontsize',16)
% xlabel('time (day)','fontsize',16)

line([-1,100],[2,2],'LineStyle','--','Color','k','linewidth',1)
line([-1,100],[3,3],'LineStyle','--','Color','k','linewidth',1)
line([-1,100],[6,6],'LineStyle','--','Color','k','linewidth',1)


indfst=sum(heaviside(FstPeak-0.01))+1;

P1x=[0,FstPeak(1:indfst),0];
P1y=[1,1:indfst,1];

indl1=sum(heaviside(L1(2:end)-0.01))+2;

P2x=[fliplr(FstPeak(1:indfst)),13,13,L1(2:indl1),0];
P2y=[indfst:-1:1,1,2,2:indl1,indfst];

P3x=[fliplr(L1(2:indl1)),L2(2:10),0,0];
```

```
P3y=[indl1:-1:2,2:10,10,indl1];

P4x=[13,13,L2(2:10),13];
P4y=[10,2,2:10,10];

hold on
patch(P1x,P1y,[0 0 0],'facealpha',0.2)
patch(P2x,P2y,[1 0 0],'facealpha',0.3)
patch(P3x,P3y,[0 1 0],'facealpha',0.3)
patch(P4x,P4y,[0 162/255 1],'facealpha',0.3)

text(8,8,'q_1 = 5\times 10^{-5}')
text(8,9,'q_2 = 1\times 10^{-8}')

%% color figures (Model R1) q1=5e-5,q2=1e-7

Itv=[1:1:14];

L1=[inf,17.72,16.66,15.66,14.66,14,14,14,14,14,14,14,14,14]-14;
L2=[NaN,24.68,24.43,22.73,21.22,20.11,19.33,18.79,18.41,18.14,17.94,17.79,17.67,17.58]
-14;
FstPeak=[15.71,14.74,14,14,14,14,14,14,14,14,14,14,14,14]-14;

figure(3)
subplot(4,4,3);
h1=plot(L1,Itv,'-k','linewidth',1);
hold on
h2=plot(L2,Itv,'-k','linewidth',1);
h3=plot(FstPeak,Itv,'-k','linewidth',1);
set(gcf,'Position',[10 200 500 400])
set(gca,'FontSize',12,'xtick',0:2:14,'ytick',1:3:10)
set(gca,'YDir','reverse')
axis([0 13 1 10])
% ylabel('inter-exposure interval (day)','fontsize',16)
% xlabel('time (day)','fontsize',16)

line([-1,100],[2,2],'LineStyle','--','Color','k','linewidth',1)
line([-1,100],[3,3],'LineStyle','--','Color','k','linewidth',1)
line([-1,100],[6,6],'LineStyle','--','Color','k','linewidth',1)


indfst=sum(heaviside(FstPeak-0.01))+1;

P1x=[0,FstPeak(1:indfst),0];
P1y=[1,1:indfst,1];

indl1=sum(heaviside(L1(2:end)-0.01))+2;

P2x=[fliplr(FstPeak(1:indfst)),13,13,L1(2:indl1),0];
P2y=[indfst:-1:1,1,2,2:indl1,indfst];

P3x=[fliplr(L1(2:indl1)),L2(2:10),0,0];
P3y=[indl1:-1:2,2:10,10,indl1];

P4x=[13,13,L2(2:10),13];
P4y=[10,2,2:10,10];

hold on
patch(P1x,P1y,[0 0 0],'facealpha',0.2)
patch(P2x,P2y,[1 0 0],'facealpha',0.3)
patch(P3x,P3y,[0 1 0],'facealpha',0.3)
patch(P4x,P4y,[0 162/255 1],'facealpha',0.3)

text(8,8,'q_1 = 5\times 10^{-5}')
text(8,9,'q_2 = 1\times 10^{-7}')

%% color figures (Model R1) q1=5e-5,q2=5e-6

Itv=[1:1:14];

L1=[inf,17.72,16.66,15.66,14.66,14,14,14,14,14,14,14,14,14]-14;
L2=[NaN,24.54,24.29,22.59,21.07,19.96,19.18,18.65,18.28,18.02,17.82,17.67,17.56,17.47]
-14;
FstPeak=[15.71,14.74,14,14,14,14,14,14,14,14,14,14,14,14]-14;
```

```
figure(3)
subplot(4,4,2);
h1=plot(L1,Itv,'-k','linewidth',1);
hold on
h2=plot(L2,Itv,'-k','linewidth',1);
h3=plot(FstPeak,Itv,'-k','linewidth',1);
set(gcf,'Position',[10 200 500 400])
set(gca,'FontSize',12,'xtick',0:2:14,'ytick',1:3:10)
set(gca,'YDir','reverse')
axis([0 13 1 10])
% ylabel('inter-exposure interval (day)','fontsize',16)
% xlabel('time (day)','fontsize',16)

line([-1,100],[2,2],'LineStyle','--','Color','k','linewidth',1)
line([-1,100],[3,3],'LineStyle','--','Color','k','linewidth',1)
line([-1,100],[6,6],'LineStyle','--','Color','k','linewidth',1)


indfst=sum(heaviside(FstPeak-0.01))+1;

P1x=[0,FstPeak(1:indfst),0];
P1y=[1,1:indfst,1];

indl1=sum(heaviside(L1(2:end)-0.01))+2;

P2x=[fliplr(FstPeak(1:indfst)),13,13,L1(2:indl1),0];
P2y=[indfst:-1:1,1,2,2:indl1,indfst];

P3x=[fliplr(L1(2:indl1)),L2(2:10),0,0];
P3y=[indl1:-1:2,2:10,10,indl1];

P4x=[13,13,L2(2:10),13];
P4y=[10,2,2:10,10];

hold on
patch(P1x,P1y,[0 0 0],'facealpha',0.2)
patch(P2x,P2y,[1 0 0],'facealpha',0.3)
patch(P3x,P3y,[0 1 0],'facealpha',0.3)
patch(P4x,P4y,[0 162/255 1],'facealpha',0.3)

text(8,8,'q_1 = 5\times 10^{-5}')
text(8,9,'q_2 = 5\times 10^{-6}')

%% color figures (Model R1) q1=5e-5,q2=5e-5

Itv=[1:1:14];

L1=[inf,17.72,16.66,15.66,14.66,14,14,14,14,14,14,14,14,14]-14;
L2=[NaN,24.07,23.85,22.12,20.58,19.46,18.68,18.18,17.84,17.59,17.41,17.28,17.18,17.10]
-14;
FstPeak=[15.71,14.74,14,14,14,14,14,14,14,14,14,14,14,14]-14;

figure(3)
subplot(4,4,1);
h1=plot(L1,Itv,'-k','linewidth',1);
hold on
h2=plot(L2,Itv,'-k','linewidth',1);
h3=plot(FstPeak,Itv,'-k','linewidth',1);
set(gcf,'Position',[10 200 500 400])
set(gca,'FontSize',12,'xtick',0:2:14,'ytick',1:3:10)
set(gca,'YDir','reverse')
axis([0 13 1 10])
ylabel('inter-exposure interval (day)','fontsize',16)
% xlabel('time (day)','fontsize',16)

line([-1,100],[2,2],'LineStyle','--','Color','k','linewidth',1)
line([-1,100],[3,3],'LineStyle','--','Color','k','linewidth',1)
line([-1,100],[6,6],'LineStyle','--','Color','k','linewidth',1)


indfst=sum(heaviside(FstPeak-0.01))+1;

P1x=[0,FstPeak(1:indfst),0];
```

```
P1y=[1,1:indfst,1];

indl1=sum(heaviside(L1(2:end)-0.01))+2;

P2x=[fliplr(FstPeak(1:indfst)),13,13,L1(2:indl1),0];
P2y=[indfst:-1:1,1,2,2:indl1,indfst];

P3x=[fliplr(L1(2:indl1)),L2(2:10),0,0];
P3y=[indl1:-1:2,2:10,10,indl1];

P4x=[13,13,L2(2:10),13];
P4y=[10,2,2:10,10];

hold on
patch(P1x,P1y,[0 0 0],'facealpha',0.2)
patch(P2x,P2y,[1 0 0],'facealpha',0.3)
patch(P3x,P3y,[0 1 0],'facealpha',0.3)
patch(P4x,P4y,[0 162/255 1],'facealpha',0.3)

text(8,8,'q_1 = 5\times 10^{-5}')
text(8,9,'q_2 = 5\times 10^{-5}')

%%
set(gcf,'Position',[10 200 800 750])
```

5. Code for generating Fig. S11 (or Fig. 10)

(Note that: there is a function called "crossing.m" used for finding zero point of a vector. A link to download it is http://au.mathworks.com/matlabcentral/fileexchange/2432-crossing.
You can also easily find it using Google search.)

```
% Using this code to generate the data for color figures
clear all;clc
% format long
%
% reinf_time=3;
%
% S0=[1,7e+7,0,0,0,0,0,0,0,0,0];
% options = odeset('RelTol',1e-12,'AbsTol',1e-12);
% [T1,Y1] = ode15s(@ODEmodel_reinfection,[0:0.01:reinf_time],S0,options);
% S1=Y1(end,:)+[0,0,0,0,0,0,0,1,0,0,0];
% options = odeset('RelTol',1e-12,'AbsTol',1e-12);
% [T2,Y2] = ode15s(@ODEmodel_reinfection,[reinf_time:0.01:reinf_time+50],S1,options);
%
% T=[T1;T2];
% Y=[Y1;Y2];
%
% Ts=14-reinf_time;
%
% figure
% set(gcf,'Position',[10 200 500 600])
% subplot(3,1,1);h11=plot(T+Ts,log10(Y(:,1)),'-k');hold
on;h12=plot(T+Ts,log10(Y(:,8)),'-r');
% ylabel('log_{10}(V)','fontsize',16)
% set(gca,'FontSize',15)
% % legend([h11,h12],'V1','V2')
% axis([0 24 -2 6])
% hold on;
% line([14,14],[-5,16],'LineStyle','--','Color','k')
% % line([0,300],[2,2],'LineStyle','--','Color','r')
% text(5,3,['interval = ',num2str(reinf_time),' days'],'fontsize',15)
%
% subplot(3,1,2);h21=plot(T+Ts,Y(:,2),'-b');hold on;h22=plot(T+Ts,Y(:,3),'-r');
% ylabel('No. of cells','fontsize',16)
% set(gca,'FontSize',15)
% % legend([h21,h22],'T','I')
% axis([0 24 0 1e+8])
% hold on;line([14,14],[0,1e+8],'LineStyle','--','Color','k')
%
% subplot(3,1,3);
% window=20;
% cf=zeros(1,length(T2)-window+1);
% for i=1:length(T2)-window+1
%     cm=corrcoef(Y2(i:i+window-1,1),Y2(i:i+window-1,8));
%     cf(i)=cm(1,2);
% end
% plot(Ts+0.01*window/2+T2(1:(length(T2)-window+1)),cf,'-ok')
% axis([0 24 -1 1])
% set(gca,'FontSize',15)
% ylabel('MC coefficient','fontsize',16)
% hold on;line([14,14],[-10,1e+8],'LineStyle','--','Color','k')
% line([0,300],[0,0],'LineStyle','--','Color','k')
%
% xlabel('time (day)','fontsize',16)
%
% % find phase-transition point using MC coefficient
% timeuse=Ts+0.01*window/2+T2(1:(length(T2)-window+1));
% ind=crossing(cf);
% indt=timeuse(ind);
% indt(1:3)
%
% % find the first peak of V2
% der=Y2(2:end,8)-Y2(1:end-1,8);
% indp=crossing(der);
% indt_peak=T2(indp)+Ts;
```

```
% indt_peak(1:2)


%% color figures (Model R2) q1=1e-8,q2=1e-8

Itv=[1:1:14];

% the data is based on both model solution and MC coefficient
% FstPeak separates Phase 1 and 2; L1 separates Phase 2 and 3; L2 separates Phase 3
and 4;

L1=[inf,21.45,19.82,18.82,17.82,16.82,15.82,14.82,14,14,14,14,14,14]-14;
L2=[NaN,29.08,27.31,26.44,24.79,23.14,21.57,20.18,19.04,18.22,17.70,17.42,17.29,17.22]
-14;
FstPeak=[16.12,15.15,14,14,14,14,14,14,14,14,14,14,14,14]-14;

figure(3)
subplot(4,4,16);
h1=plot(L1,Itv,'-k','linewidth',1);
hold on
h2=plot(L2,Itv,'-k','linewidth',1);
h3=plot(FstPeak,Itv,'-k','linewidth',1);
set(gcf,'Position',[10 200 500 400])
set(gca,'FontSize',12,'xtick',0:2:14,'ytick',1:3:10)
set(gca,'YDir','reverse')
axis([0 13 1 10])
% ylabel('inter-exposure interval (day)','fontsize',16)
xlabel('time (day)','fontsize',16)

line([-1,100],[2,2],'LineStyle','--','Color','k','linewidth',1)
line([-1,100],[3,3],'LineStyle','--','Color','k','linewidth',1)
line([-1,100],[9,9],'LineStyle','--','Color','k','linewidth',1)

indfst=sum(heaviside(FstPeak-0.01))+1;

P1x=[0,FstPeak(1:indfst),0];
P1y=[1,1:indfst,1];

indl1=sum(heaviside(L1(2:end)-0.01))+2;

P2x=[fliplr(FstPeak(1:indfst)),13,13,L1(2:indl1),0];
P2y=[indfst:-1:1,1,2,2:indl1,indfst];

P3x=[fliplr(L1(2:indl1)),L2(2:10),0,0];
P3y=[indl1:-1:2,2:10,10,indl1];

P4x=[13,13,L2(2:10),13];
P4y=[10,2,2:10,10];

hold on
patch(P1x,P1y,[0 0 0],'facealpha',0.2)
patch(P2x,P2y,[1 0 0],'facealpha',0.3)
patch(P3x,P3y,[0 1 0],'facealpha',0.3)
patch(P4x,P4y,[0 162/255 1],'facealpha',0.3)

text(8,8,'q_1 = 1\times 10^{-8}')
text(8,9,'q_2 = 1\times 10^{-8}')

%% color figures (Model R2) q1=1e-8,q2=1e-7

Itv=[1:1:14];

L1=[inf,21.45,19.82,18.82,17.82,16.82,15.82,14.82,14,14,14,14,14,14]-14;
L2=[NaN,27.83,27.36,26.49,24.84,23.18,21.62,20.22,19.08,18.26,17.75,17.47,17.33,17.27]
-14;
FstPeak=[16.12,15.15,14,14,14,14,14,14,14,14,14,14,14,14]-14;

figure(3)
subplot(4,4,15);
h1=plot(L1,Itv,'-k','linewidth',1);
hold on
h2=plot(L2,Itv,'-k','linewidth',1);
h3=plot(FstPeak,Itv,'-k','linewidth',1);
set(gcf,'Position',[10 200 500 400])
```

```
set(gca,'FontSize',12,'xtick',0:2:14,'ytick',1:3:10)
set(gca,'YDir','reverse')
axis([0 13 1 10])
% ylabel('inter-exposure interval (day)','fontsize',16)
% xlabel('time (day)','fontsize',16)

line([-1,100],[2,2],'LineStyle','--','Color','k','linewidth',1)
line([-1,100],[3,3],'LineStyle','--','Color','k','linewidth',1)
line([-1,100],[9,9],'LineStyle','--','Color','k','linewidth',1)


indfst=sum(heaviside(FstPeak-0.01))+1;

P1x=[0,FstPeak(1:indfst),0];
P1y=[1,1:indfst,1];

indl1=sum(heaviside(L1(2:end)-0.01))+2;

P2x=[fliplr(FstPeak(1:indfst)),13,13,L1(2:indl1),0];
P2y=[indfst:-1:1,1,2,2:indl1,indfst];

P3x=[fliplr(L1(2:indl1)),L2(2:10),0,0];
P3y=[indl1:-1:2,2:10,10,indl1];

P4x=[13,13,L2(2:10),13];
P4y=[10,2,2:10,10];

hold on
patch(P1x,P1y,[0 0 0],'facealpha',0.2)
patch(P2x,P2y,[1 0 0],'facealpha',0.3)
patch(P3x,P3y,[0 1 0],'facealpha',0.3)
patch(P4x,P4y,[0 162/255 1],'facealpha',0.3)

text(8,8,'q_1 = 1\times 10^{-8}')
text(8,9,'q_2 = 1\times 10^{-7}')

%% color figures (Model R2) q1=1e-8,q2=5e-6

Itv=[1:1:14];

L1=[inf,21.44,19.82,18.82,17.82,16.82,15.82,14.82,14,14,14,14,14,14]-14;
L2=[NaN,28.30,26.55,25.68,24.02,22.36,20.78,19.38,18.23,17.42,16.92,16.65,16.52,16.46]
-14;
FstPeak=[16.14,15.15,14,14,14,14,14,14,14,14,14,14,14,14]-14;

figure(3)
subplot(4,4,14);
h1=plot(L1,Itv,'-k','linewidth',1);
hold on
h2=plot(L2,Itv,'-k','linewidth',1);
h3=plot(FstPeak,Itv,'-k','linewidth',1);
set(gcf,'Position',[10 200 500 400])
set(gca,'FontSize',12,'xtick',0:2:14,'ytick',1:3:10)
set(gca,'YDir','reverse')
axis([0 13 1 10])
% ylabel('inter-exposure interval (day)','fontsize',16)
% xlabel('time (day)','fontsize',16)

line([-1,100],[2,2],'LineStyle','--','Color','k','linewidth',1)
line([-1,100],[3,3],'LineStyle','--','Color','k','linewidth',1)
line([-1,100],[9,9],'LineStyle','--','Color','k','linewidth',1)


indfst=sum(heaviside(FstPeak-0.01))+1;

P1x=[0,FstPeak(1:indfst),0];
P1y=[1,1:indfst,1];

indl1=sum(heaviside(L1(2:end)-0.01))+2;

P2x=[fliplr(FstPeak(1:indfst)),13,13,L1(2:indl1),0];
P2y=[indfst:-1:1,1,2,2:indl1,indfst];

P3x=[fliplr(L1(2:indl1)),L2(2:10),0,0];
```

```
P3y=[indl1:-1:2,2:10,10,indl1];

P4x=[13,13,L2(2:10),13];
P4y=[10,2,2:10,10];

hold on
patch(P1x,P1y,[0 0 0],'facealpha',0.2)
patch(P2x,P2y,[1 0 0],'facealpha',0.3)
patch(P3x,P3y,[0 1 0],'facealpha',0.3)
patch(P4x,P4y,[0 162/255 1],'facealpha',0.3)

text(8,8,'q_1 = 1\times 10^{-8}')
text(8,9,'q_2 = 5\times 10^{-6}')

%% color figures (Model R2) q1=1e-8,q2=5e-5

Itv=[1:1:14];

L1=[inf,21.34,19.82,18.82,17.82,16.82,15.82,14.82,14,14,14,14,14,14]-14;
L2=[NaN,27.61,26.07,25.20,23.54,21.87,20.28,18.85,17.70,16.89,16.41,16.16,16.04,15.99]
-14;
FstPeak=[15.97,15.15,14,14,14,14,14,14,14,14,14,14,14,14]-14;

figure(3)
subplot(4,4,13);
h1=plot(L1,Itv,'-k','linewidth',1);
hold on
h2=plot(L2,Itv,'-k','linewidth',1);
h3=plot(FstPeak,Itv,'-k','linewidth',1);
set(gcf,'Position',[10 200 500 400])
set(gca,'FontSize',12,'xtick',0:2:14,'ytick',1:3:10)
set(gca,'YDir','reverse')
axis([0 13 1 10])
% ylabel('inter-exposure interval (day)','fontsize',16)
% xlabel('time (day)','fontsize',16)

line([-1,100],[2,2],'LineStyle','--','Color','k','linewidth',1)
line([-1,100],[3,3],'LineStyle','--','Color','k','linewidth',1)
line([-1,100],[9,9],'LineStyle','--','Color','k','linewidth',1)


indfst=sum(heaviside(FstPeak-0.01))+1;

P1x=[0,FstPeak(1:indfst),0];
P1y=[1,1:indfst,1];

indl1=sum(heaviside(L1(2:end)-0.01))+2;

P2x=[fliplr(FstPeak(1:indfst)),13,13,L1(2:indl1),0];
P2y=[indfst:-1:1,1,2,2:indl1,indfst];

P3x=[fliplr(L1(2:indl1)),L2(2:10),0,0];
P3y=[indl1:-1:2,2:10,10,indl1];

P4x=[13,13,L2(2:10),13];
P4y=[10,2,2:10,10];

hold on
patch(P1x,P1y,[0 0 0],'facealpha',0.2)
patch(P2x,P2y,[1 0 0],'facealpha',0.3)
patch(P3x,P3y,[0 1 0],'facealpha',0.3)
patch(P4x,P4y,[0 162/255 1],'facealpha',0.3)

text(8,8,'q_1 = 1\times 10^{-8}')
text(8,9,'q_2 = 5\times 10^{-5}')

%% color figures (Model R2) q1=1e-7,q2=1e-8

Itv=[1:1:14];

L1=[inf,18.51,17.22,16.22,15.22,14,14,14,14,14,14,14,14,14]-14;
L2=[NaN,23.78,23.09,22.08,20.70,19.44,18.49,17.86,17.51,17.33,17.24,17.20,17.18,17.17]
-14;
FstPeak=[16.13,15.20,14,14,14,14,14,14,14,14,14,14,14,14]-14;
```

```
figure(3)
subplot(4,4,12);
h1=plot(L1,Itv,'-k','linewidth',1);
hold on
h2=plot(L2,Itv,'-k','linewidth',1);
h3=plot(FstPeak,Itv,'-k','linewidth',1);
set(gcf,'Position',[10 200 500 400])
set(gca,'FontSize',12,'xtick',0:2:14,'ytick',1:3:10)
set(gca,'YDir','reverse')
axis([0 13 1 10])
% ylabel('inter-exposure interval (day)','fontsize',16)
% xlabel('time (day)','fontsize',16)

line([-1,100],[2,2],'LineStyle','--','Color','k','linewidth',1)
line([-1,100],[3,3],'LineStyle','--','Color','k','linewidth',1)
line([-1,100],[6,6],'LineStyle','--','Color','k','linewidth',1)


indfst=sum(heaviside(FstPeak-0.01))+1;

P1x=[0,FstPeak(1:indfst),0];
P1y=[1,1:indfst,1];

indl1=sum(heaviside(L1(2:end)-0.01))+2;

P2x=[fliplr(FstPeak(1:indfst)),13,13,L1(2:indl1),0];
P2y=[indfst:-1:1,1,2,2:indl1,indfst];

P3x=[fliplr(L1(2:indl1)),L2(2:10),0,0];
P3y=[indl1:-1:2,2:10,10,indl1];

P4x=[13,13,L2(2:10),13];
P4y=[10,2,2:10,10];

hold on
patch(P1x,P1y,[0 0 0],'facealpha',0.2)
patch(P2x,P2y,[1 0 0],'facealpha',0.3)
patch(P3x,P3y,[0 1 0],'facealpha',0.3)
patch(P4x,P4y,[0 162/255 1],'facealpha',0.3)

text(8,8,'q_1 = 1\times 10^{-7}')
text(8,9,'q_2 = 1\times 10^{-8}')

%% color figures (Model R2) q1=1e-7,q2=1e-7

Itv=[1:1:14];

L1=[inf,18.51,17.22,16.22,15.22,14,14,14,14,14,14,14,14,14]-14;
L2=[NaN,23.83,23.13,22.12,20.74,19.48,18.53,17.91,17.55,17.37,17.29,17.25,17.23,17.22]
-14;
FstPeak=[16.12,15.20,14,14,14,14,14,14,14,14,14,14,14,14]-14;

figure(3)
subplot(4,4,11);
h1=plot(L1,Itv,'-k','linewidth',1);
hold on
h2=plot(L2,Itv,'-k','linewidth',1);
h3=plot(FstPeak,Itv,'-k','linewidth',1);
set(gcf,'Position',[10 200 500 400])
set(gca,'FontSize',12,'xtick',0:2:14,'ytick',1:3:10)
set(gca,'YDir','reverse')
axis([0 13 1 10])
% ylabel('inter-exposure interval (day)','fontsize',16)
% xlabel('time (day)','fontsize',16)

line([-1,100],[2,2],'LineStyle','--','Color','k','linewidth',1)
line([-1,100],[3,3],'LineStyle','--','Color','k','linewidth',1)
line([-1,100],[6,6],'LineStyle','--','Color','k','linewidth',1)


indfst=sum(heaviside(FstPeak-0.01))+1;

P1x=[0,FstPeak(1:indfst),0];
```

```
P1y=[1,1:indfst,1];

indl1=sum(heaviside(L1(2:end)-0.01))+2;

P2x=[fliplr(FstPeak(1:indfst)),13,13,L1(2:indl1),0];
P2y=[indfst:-1:1,1,2,2:indl1,indfst];

P3x=[fliplr(L1(2:indl1)),L2(2:10),0,0];
P3y=[indl1:-1:2,2:10,10,indl1];

P4x=[13,13,L2(2:10),13];
P4y=[10,2,2:10,10];

hold on
patch(P1x,P1y,[0 0 0],'facealpha',0.2)
patch(P2x,P2y,[1 0 0],'facealpha',0.3)
patch(P3x,P3y,[0 1 0],'facealpha',0.3)
patch(P4x,P4y,[0 162/255 1],'facealpha',0.3)

text(8,8,'q_1 = 1\times 10^{-7}')
text(8,9,'q_2 = 1\times 10^{-7}')

%% color figures (Model R2) q1=1e-7,q2=5e-6

Itv=[1:1:14];

L1=[inf,18.52,17.22,16.22,15.22,14,14,14,14,14,14,14,14,14]-14;
L2=[NaN,23.00,22.30,21.30,19.91,18.65,17.69,17.08,16.74,16.56,16.48,16.44,16.43,16.42]
-14;
FstPeak=[16.12,15.20,14,14,14,14,14,14,14,14,14,14,14,14]-14;

figure(3)
subplot(4,4,10);
h1=plot(L1,Itv,'-k','linewidth',1);
hold on
h2=plot(L2,Itv,'-k','linewidth',1);
h3=plot(FstPeak,Itv,'-k','linewidth',1);
set(gcf,'Position',[10 200 500 400])
set(gca,'FontSize',12,'xtick',0:2:14,'ytick',1:3:10)
set(gca,'YDir','reverse')
axis([0 13 1 10])
% ylabel('inter-exposure interval (day)','fontsize',16)
% xlabel('time (day)','fontsize',16)

line([-1,100],[2,2],'LineStyle','--','Color','k','linewidth',1)
line([-1,100],[3,3],'LineStyle','--','Color','k','linewidth',1)
line([-1,100],[6,6],'LineStyle','--','Color','k','linewidth',1)


indfst=sum(heaviside(FstPeak-0.01))+1;

P1x=[0,FstPeak(1:indfst),0];
P1y=[1,1:indfst,1];

indl1=sum(heaviside(L1(2:end)-0.01))+2;

P2x=[fliplr(FstPeak(1:indfst)),13,13,L1(2:indl1),0];
P2y=[indfst:-1:1,1,2,2:indl1,indfst];

P3x=[fliplr(L1(2:indl1)),L2(2:10),0,0];
P3y=[indl1:-1:2,2:10,10,indl1];

P4x=[13,13,L2(2:10),13];
P4y=[10,2,2:10,10];

hold on
patch(P1x,P1y,[0 0 0],'facealpha',0.2)
patch(P2x,P2y,[1 0 0],'facealpha',0.3)
patch(P3x,P3y,[0 1 0],'facealpha',0.3)
patch(P4x,P4y,[0 162/255 1],'facealpha',0.3)

text(8,8,'q_1 = 1\times 10^{-7}')
text(8,9,'q_2 = 5\times 10^{-6}')
```

```
%% color figures (Model R2) q1=1e-7,q2=5e-5

Itv=[1:1:14];

L1=[inf,18.48,17.22,16.22,15.22,14,14,14,14,14,14,14,14,14]-14;
L2=[NaN,22.46,21.80,20.79,19.38,18.11,17.16,16.56,16.24,16.08,16.00,15.97,15.95,15.94]
-14;
FstPeak=[15.92,15.20,14,14,14,14,14,14,14,14,14,14,14,14]-14;

figure(3)
subplot(4,4,9);
h1=plot(L1,Itv,'-k','linewidth',1);
hold on
h2=plot(L2,Itv,'-k','linewidth',1);
h3=plot(FstPeak,Itv,'-k','linewidth',1);
set(gcf,'Position',[10 200 500 400])
set(gca,'FontSize',12,'xtick',0:2:14,'ytick',1:3:10)
set(gca,'YDir','reverse')
axis([0 13 1 10])
% ylabel('inter-exposure interval (day)','fontsize',16)
% xlabel('time (day)','fontsize',16)

line([-1,100],[2,2],'LineStyle','--','Color','k','linewidth',1)
line([-1,100],[3,3],'LineStyle','--','Color','k','linewidth',1)
line([-1,100],[6,6],'LineStyle','--','Color','k','linewidth',1)


indfst=sum(heaviside(FstPeak-0.01))+1;

P1x=[0,FstPeak(1:indfst),0];
P1y=[1,1:indfst,1];

indl1=sum(heaviside(L1(2:end)-0.01))+2;

P2x=[fliplr(FstPeak(1:indfst)),13,13,L1(2:indl1),0];
P2y=[indfst:-1:1,1,2,2:indl1,indfst];

P3x=[fliplr(L1(2:indl1)),L2(2:10),0,0];
P3y=[indl1:-1:2,2:10,10,indl1];

P4x=[13,13,L2(2:10),13];
P4y=[10,2,2:10,10];

hold on
patch(P1x,P1y,[0 0 0],'facealpha',0.2)
patch(P2x,P2y,[1 0 0],'facealpha',0.3)
patch(P3x,P3y,[0 1 0],'facealpha',0.3)
patch(P4x,P4y,[0 162/255 1],'facealpha',0.3)

text(8,8,'q_1 = 1\times 10^{-7}')
text(8,9,'q_2 = 5\times 10^{-5}')

%% color figures (Model R2) q1=5e-6,q2=1e-8

Itv=[1:1:14];

L1=[16.75,15.45,14.45,14,14,14,14,14,14,14,14,14,14,14]-14;
L2=[21.97,21.61,20.82,19.81,18.97,18.29,17.75,17.36,17.21,17.17,17.17,17.17,17.16,17.1
6]-14;
FstPeak=[15.45,14.49,14,14,14,14,14,14,14,14,14,14,14,14]-14;

figure(3)
subplot(4,4,8);
h1=plot(L1,Itv,'-k','linewidth',1);
hold on
h2=plot(L2,Itv,'-k','linewidth',1);
h3=plot(FstPeak,Itv,'-k','linewidth',1);
set(gcf,'Position',[10 200 500 400])
set(gca,'FontSize',12,'xtick',0:2:14,'ytick',1:3:10)
set(gca,'YDir','reverse')
axis([0 13 1 10])
% ylabel('inter-exposure interval (day)','fontsize',16)
% xlabel('time (day)','fontsize',16)
```

```
line([-1,100],[3,3],'LineStyle','--','Color','k','linewidth',1)
line([-1,100],[4,4],'LineStyle','--','Color','k','linewidth',1)

indfst=sum(heaviside(FstPeak-0.01))+1;

P1x=[0,FstPeak(1:indfst),0];
P1y=[1,1:indfst,1];

indl1=sum(heaviside(L1-0.01))+1;

P2x=[fliplr(FstPeak(1:indfst)),L1(1:indl1),0];
P2y=[indfst:-1:1,1:indl1,indfst];

P3x=[fliplr(L1(1:indl1)),L2(1:10),0,0];
P3y=[indl1:-1:1,1:10,10,indl1];

P4x=[13,13,L2(1:10),13];
P4y=[10,1,1:10,10];

hold on
patch(P1x,P1y,[0 0 0],'facealpha',0.2)
patch(P2x,P2y,[1 0 0],'facealpha',0.3)
patch(P3x,P3y,[0 1 0],'facealpha',0.3)
patch(P4x,P4y,[0 162/255 1],'facealpha',0.3)

text(8,8,'q_1 = 5\times 10^{-6}')
text(8,9,'q_2 = 1\times 10^{-8}')

%% color figures (Model R2) q1=5e-6,q2=1e-7

Itv=[1:1:14];

L1=[16.75,15.45,14.45,14,14,14,14,14,14,14,14,14,14,14]-14;
L2=[22.04,21.69,20.89,19.88,19.03,18.35,17.80,17.42,17.26,17.23,17.22,17.22,17.22,17.2
2]-14;
FstPeak=[15.45,14.48,14,14,14,14,14,14,14,14,14,14,14,14]-14;

figure(3)
subplot(4,4,7);
h1=plot(L1,Itv,'-k','linewidth',1);
hold on
h2=plot(L2,Itv,'-k','linewidth',1);
h3=plot(FstPeak,Itv,'-k','linewidth',1);
set(gcf,'Position',[10 200 500 400])
set(gca,'FontSize',12,'xtick',0:2:14,'ytick',1:3:10)
set(gca,'YDir','reverse')
axis([0 13 1 10])
% ylabel('inter-exposure interval (day)','fontsize',16)
% xlabel('time (day)','fontsize',16)

line([-1,100],[3,3],'LineStyle','--','Color','k','linewidth',1)
line([-1,100],[4,4],'LineStyle','--','Color','k','linewidth',1)

indfst=sum(heaviside(FstPeak-0.01))+1;

P1x=[0,FstPeak(1:indfst),0];
P1y=[1,1:indfst,1];

indl1=sum(heaviside(L1-0.01))+1;

P2x=[fliplr(FstPeak(1:indfst)),L1(1:indl1),0];
P2y=[indfst:-1:1,1:indl1,indfst];

P3x=[fliplr(L1(1:indl1)),L2(1:10),0,0];
P3y=[indl1:-1:1,1:10,10,indl1];

P4x=[13,13,L2(1:10),13];
P4y=[10,1,1:10,10];

hold on
patch(P1x,P1y,[0 0 0],'facealpha',0.2)
patch(P2x,P2y,[1 0 0],'facealpha',0.3)
patch(P3x,P3y,[0 1 0],'facealpha',0.3)
patch(P4x,P4y,[0 162/255 1],'facealpha',0.3)
```

```
text(8,8,'q_1 = 5\times 10^{-6}')
text(8,9,'q_2 = 1\times 10^{-7}')

%% color figures (Model R2) q1=5e-6,q2=5e-6

Itv=[1:1:14];

L1=[16.75,15.45,14.45,14,14,14,14,14,14,14,14,14,14,14]-14;
L2=[21.01,20.85,20.07,19.06,18.21,17.54,16.99,16.60,16.45,16.41,16.41,16.41,16.41,16.4
1]-14;
FstPeak=[15.45,14.48,14,14,14,14,14,14,14,14,14,14,14,14]-14;

figure(3)
subplot(4,4,6);
h1=plot(L1,Itv,'-k','linewidth',1);
hold on
h2=plot(L2,Itv,'-k','linewidth',1);
h3=plot(FstPeak,Itv,'-k','linewidth',1);
set(gcf,'Position',[10 200 500 400])
set(gca,'FontSize',12,'xtick',0:2:14,'ytick',1:3:10)
set(gca,'YDir','reverse')
axis([0 13 1 10])
% ylabel('inter-exposure interval (day)','fontsize',16)
% xlabel('time (day)','fontsize',16)

line([-1,100],[3,3],'LineStyle','--','Color','k','linewidth',1)
line([-1,100],[4,4],'LineStyle','--','Color','k','linewidth',1)

indfst=sum(heaviside(FstPeak-0.01))+1;

P1x=[0,FstPeak(1:indfst),0];
P1y=[1,1:indfst,1];

indl1=sum(heaviside(L1-0.01))+1;

P2x=[fliplr(FstPeak(1:indfst)),L1(1:indl1),0];
P2y=[indfst:-1:1,1:indl1,indfst];

P3x=[fliplr(L1(1:indl1)),L2(1:10),0,0];
P3y=[indl1:-1:1,1:10,10,indl1];

P4x=[13,13,L2(1:10),13];
P4y=[10,1,1:10,10];

hold on
patch(P1x,P1y,[0 0 0],'facealpha',0.2)
patch(P2x,P2y,[1 0 0],'facealpha',0.3)
patch(P3x,P3y,[0 1 0],'facealpha',0.3)
patch(P4x,P4y,[0 162/255 1],'facealpha',0.3)

text(8,8,'q_1 = 5\times 10^{-6}')
text(8,9,'q_2 = 5\times 10^{-6}')

%% color figures (Model R2) q1=5e-6,q2=5e-5

Itv=[1:1:14];

L1=[16.75,15.45,14.45,14,14,14,14,14,14,14,14,14,14,14]-14;
L2=[20.15,20.28,19.54,18.53,17.71,17.06,16.52,16.14,15.98,15.95,15.94,15.94,15.94,15.9
4]-14;
FstPeak=[15.44,14.48,14,14,14,14,14,14,14,14,14,14,14,14]-14;

figure(3)
subplot(4,4,5);
h1=plot(L1,Itv,'-k','linewidth',1);
hold on
h2=plot(L2,Itv,'-k','linewidth',1);
h3=plot(FstPeak,Itv,'-k','linewidth',1);
set(gcf,'Position',[10 200 500 400])
set(gca,'FontSize',12,'xtick',0:2:14,'ytick',1:3:10)
set(gca,'YDir','reverse')
axis([0 13 1 10])
% ylabel('inter-exposure interval (day)','fontsize',16)
```

```
% xlabel('time (day)','fontsize',16)

line([-1,100],[3,3],'LineStyle','--','Color','k','linewidth',1)
line([-1,100],[4,4],'LineStyle','--','Color','k','linewidth',1)

indfst=sum(heaviside(FstPeak-0.01))+1;

P1x=[0,FstPeak(1:indfst),0];
P1y=[1,1:indfst,1];

indl1=sum(heaviside(L1-0.01))+1;

P2x=[fliplr(FstPeak(1:indfst)),L1(1:indl1),0];
P2y=[indfst:-1:1,1:indl1,indfst];

P3x=[fliplr(L1(1:indl1)),L2(1:10),0,0];
P3y=[indl1:-1:1,1:10,10,indl1];

P4x=[13,13,L2(1:10),13];
P4y=[10,1,1:10,10];

hold on
patch(P1x,P1y,[0 0 0],'facealpha',0.2)
patch(P2x,P2y,[1 0 0],'facealpha',0.3)
patch(P3x,P3y,[0 1 0],'facealpha',0.3)
patch(P4x,P4y,[0 162/255 1],'facealpha',0.3)

text(8,8,'q_1 = 5\times 10^{-6}')
text(8,9,'q_2 = 5\times 10^{-5}')

%% color figures (Model R2) q1=5e-5,q2=1e-8

Itv=[1:1:14];

L1=[16.05,15.01,14,14,14,14,14,14,14,14,14,14,14,14]-14;
L2=[22.02,21.66,20.67,19.78,18.96,18.36,17.87,17.51,17.30,17.20,17.17,17.17,17.16,17.1
6]-14;
FstPeak=[15.00,14,14,14,14,14,14,14,14,14,14,14,14,14]-14;

figure(3)
subplot(4,4,4);
h1=plot(L1,Itv,'-k','linewidth',1);
hold on
h2=plot(L2,Itv,'-k','linewidth',1);
h3=plot(FstPeak,Itv,'-k','linewidth',1);
set(gcf,'Position',[10 200 500 400])
set(gca,'FontSize',12,'xtick',0:2:14,'ytick',1:3:10)
set(gca,'YDir','reverse')
axis([0 13 1 10])
% ylabel('inter-exposure interval (day)','fontsize',16)
% xlabel('time (day)','fontsize',16)

line([-1,100],[2,2],'LineStyle','--','Color','k','linewidth',1)
line([-1,100],[3,3],'LineStyle','--','Color','k','linewidth',1)

indfst=sum(heaviside(FstPeak-0.01))+1;

P1x=[0,FstPeak(1:indfst),0];
P1y=[1,1:indfst,1];

indl1=sum(heaviside(L1-0.01))+1;

P2x=[fliplr(FstPeak(1:indfst)),L1(1:indl1),0];
P2y=[indfst:-1:1,1:indl1,indfst];

P3x=[fliplr(L1(1:indl1)),L2(1:10),0,0];
P3y=[indl1:-1:1,1:10,10,indl1];

P4x=[13,13,L2(1:10),13];
P4y=[10,1,1:10,10];

hold on
patch(P1x,P1y,[0 0 0],'facealpha',0.2)
patch(P2x,P2y,[1 0 0],'facealpha',0.3)
```

```
patch(P3x,P3y,[0 1 0],'facealpha',0.3)
patch(P4x,P4y,[0 162/255 1],'facealpha',0.3)

text(8,8,'q_1 = 5\times 10^{-5}')
text(8,9,'q_2 = 1\times 10^{-8}')

%% color figures (Model R2) q1=5e-5,q2=1e-7

Itv=[1:1:14];

L1=[16.05,15.01,14,14,14,14,14,14,14,14,14,14,14,14]-14;
L2=[22.11,21.73,20.74,19.79,19.02,18.42,17.92,17.56,17.35,17.25,17.22,17.21,17.21,17.2
1]-14;
FstPeak=[15.00,14,14,14,14,14,14,14,14,14,14,14,14,14]-14;

figure(3)
subplot(4,4,3);
h1=plot(L1,Itv,'-k','linewidth',1);
hold on
h2=plot(L2,Itv,'-k','linewidth',1);
h3=plot(FstPeak,Itv,'-k','linewidth',1);
set(gcf,'Position',[10 200 500 400])
set(gca,'FontSize',12,'xtick',0:2:14,'ytick',1:3:10)
set(gca,'YDir','reverse')
axis([0 13 1 10])
% ylabel('inter-exposure interval (day)','fontsize',16)
% xlabel('time (day)','fontsize',16)

line([-1,100],[2,2],'LineStyle','--','Color','k','linewidth',1)
line([-1,100],[3,3],'LineStyle','--','Color','k','linewidth',1)

indfst=sum(heaviside(FstPeak-0.01))+1;

P1x=[0,FstPeak(1:indfst),0];
P1y=[1,1:indfst,1];

indl1=sum(heaviside(L1-0.01))+1;

P2x=[fliplr(FstPeak(1:indfst)),L1(1:indl1),0];
P2y=[indfst:-1:1,1:indl1,indfst];

P3x=[fliplr(L1(1:indl1)),L2(1:10),0,0];
P3y=[indl1:-1:1,1:10,10,indl1];

P4x=[13,13,L2(1:10),13];
P4y=[10,1,1:10,10];

hold on
patch(P1x,P1y,[0 0 0],'facealpha',0.2)
patch(P2x,P2y,[1 0 0],'facealpha',0.3)
patch(P3x,P3y,[0 1 0],'facealpha',0.3)
patch(P4x,P4y,[0 162/255 1],'facealpha',0.3)

text(8,8,'q_1 = 5\times 10^{-5}')
text(8,9,'q_2 = 1\times 10^{-7}')

%% color figures (Model R2) q1=5e-5,q2=5e-6

Itv=[1:1:14];

L1=[16.05,15.01,14,14,14,14,14,14,14,14,14,14,14,14]-14;
L2=[21.14,20.85,19.85,18.91,18.16,17.58,17.10,16.75,16.54,16.45,16.42,16.41,16.41,16.4
1]-14;
FstPeak=[15.00,14,14,14,14,14,14,14,14,14,14,14,14,14]-14;

figure(3)
subplot(4,4,2);
h1=plot(L1,Itv,'-k','linewidth',1);
hold on
h2=plot(L2,Itv,'-k','linewidth',1);
h3=plot(FstPeak,Itv,'-k','linewidth',1);
set(gcf,'Position',[10 200 500 400])
set(gca,'FontSize',12,'xtick',0:2:14,'ytick',1:3:10)
set(gca,'YDir','reverse')
```

```
axis([0 13 1 10])
% ylabel('inter-exposure interval (day)','fontsize',16)
% xlabel('time (day)','fontsize',16)

line([-1,100],[2,2],'LineStyle','--','Color','k','linewidth',1)
line([-1,100],[3,3],'LineStyle','--','Color','k','linewidth',1)

indfst=sum(heaviside(FstPeak-0.01))+1;

P1x=[0,FstPeak(1:indfst),0];
P1y=[1,1:indfst,1];

indl1=sum(heaviside(L1-0.01))+1;

P2x=[fliplr(FstPeak(1:indfst)),L1(1:indl1),0];
P2y=[indfst:-1:1,1:indl1,indfst];

P3x=[fliplr(L1(1:indl1)),L2(1:10),0,0];
P3y=[indl1:-1:1,1:10,10,indl1];

P4x=[13,13,L2(1:10),13];
P4y=[10,1,1:10,10];

hold on
patch(P1x,P1y,[0 0 0],'facealpha',0.2)
patch(P2x,P2y,[1 0 0],'facealpha',0.3)
patch(P3x,P3y,[0 1 0],'facealpha',0.3)
patch(P4x,P4y,[0 162/255 1],'facealpha',0.3)

text(8,8,'q_1 = 5\times 10^{-5}')
text(8,9,'q_2 = 5\times 10^{-6}')

%% color figures (Model R2) q1=5e-5,q2=5e-5

Itv=[1:1:14];

L1=[16.04,15.01,14,14,14,14,14,14,14,14,14,14,14,14]-14;
L2=[20.41,20.24,19.25,18.31,17.60,17.05,16.60,16.27,16.07,15.98,15.95,15.94,15.94,15.9
4]-14;
FstPeak=[15.00,14,14,14,14,14,14,14,14,14,14,14,14,14]-14;

figure(3)
subplot(4,4,1);
h1=plot(L1,Itv,'-k','linewidth',1);
hold on
h2=plot(L2,Itv,'-k','linewidth',1);
h3=plot(FstPeak,Itv,'-k','linewidth',1);
set(gcf,'Position',[10 200 500 400])
set(gca,'FontSize',12,'xtick',0:2:14,'ytick',1:3:10)
set(gca,'YDir','reverse')
axis([0 13 1 10])
ylabel('inter-exposure interval (day)','fontsize',16)
% xlabel('time (day)','fontsize',16)

line([-1,100],[2,2],'LineStyle','--','Color','k','linewidth',1)
line([-1,100],[3,3],'LineStyle','--','Color','k','linewidth',1)

indfst=sum(heaviside(FstPeak-0.01))+1;

P1x=[0,FstPeak(1:indfst),0];
P1y=[1,1:indfst,1];

indl1=sum(heaviside(L1-0.01))+1;

P2x=[fliplr(FstPeak(1:indfst)),L1(1:indl1),0];
P2y=[indfst:-1:1,1:indl1,indfst];

P3x=[fliplr(L1(1:indl1)),L2(1:10),0,0];
P3y=[indl1:-1:1,1:10,10,indl1];

P4x=[13,13,L2(1:10),13];
P4y=[10,1,1:10,10];

hold on
```

```
patch(P1x,P1y,[0 0 0],'facealpha',0.2)
patch(P2x,P2y,[1 0 0],'facealpha',0.3)
patch(P3x,P3y,[0 1 0],'facealpha',0.3)
patch(P4x,P4y,[0 162/255 1],'facealpha',0.3)

text(8,8,'q_1 = 5\times 10^{-5}')
text(8,9,'q_2 = 5\times 10^{-5}')

%%
set(gcf,'Position',[10 200 800 750])
```

6. Code for generating Fig. S12 (or Fig. 11)

(Note that: there is a function called "crossing.m" used for finding zero point of a vector. A link to download it is http://au.mathworks.com/matlabcentral/fileexchange/2432-crossing. **You can also easily find it using Google search.)**

```
% Using this code to generate the data for color figures
clear all;clc
% format long
%
% reinf_time=3;
%
% S0=[1,7e+7,0,0,0,0,0,0,0,0,0];
% options = odeset('RelTol',1e-12,'AbsTol',1e-12);
% [T1,Y1] = ode15s(@ODEmodel_reinfection,[0:0.01:reinf_time],S0,options);
% S1=Y1(end,:)+[0,0,0,0,0,0,0,1,0,0,0];
% options = odeset('RelTol',1e-12,'AbsTol',1e-12);
% [T2,Y2] = ode15s(@ODEmodel_reinfection,[reinf_time:0.01:reinf_time+50],S1,options);
%
% T=[T1;T2];
% Y=[Y1;Y2];
%
% Ts=14-reinf_time;
%
% figure
% set(gcf,'Position',[10 200 500 600])
% subplot(3,1,1);h11=plot(T+Ts,log10(Y(:,1)),'-k');hold
on;h12=plot(T+Ts,log10(Y(:,8)),'-r');
% ylabel('log_{10}(V)','fontsize',16)
% set(gca,'FontSize',15)
% % legend([h11,h12],'V1','V2')
% axis([0 24 -2 6])
% hold on;
% line([14,14],[-5,16],'LineStyle','--','Color','k')
% % line([0,300],[2,2],'LineStyle','--','Color','r')
% text(5,3,['interval = ',num2str(reinf_time),' days'],'fontsize',15)
%
% subplot(3,1,2);h21=plot(T+Ts,Y(:,2),'-b');hold on;h22=plot(T+Ts,Y(:,3),'-r');
% ylabel('No. of cells','fontsize',16)
% set(gca,'FontSize',15)
% % legend([h21,h22],'T','I')
% axis([0 24 0 1e+8])
% hold on;line([14,14],[0,1e+8],'LineStyle','--','Color','k')
%
% subplot(3,1,3);
% window=20;
% cf=zeros(1,length(T2)-window+1);
% for i=1:length(T2)-window+1
%     cm=corrcoef(Y2(i:i+window-1,1),Y2(i:i+window-1,8));
%     cf(i)=cm(1,2);
% end
% plot(Ts+0.01*window/2+T2(1:(length(T2)-window+1)),cf,'-ok')
% axis([0 24 -1 1])
% set(gca,'FontSize',15)
% ylabel('MC coefficient','fontsize',16)
% hold on;line([14,14],[-10,1e+8],'LineStyle','--','Color','k')
% line([0,300],[0,0],'LineStyle','--','Color','k')
%
% xlabel('time (day)','fontsize',16)
%
% % find phase-transition point using MC coefficient
% timeuse=Ts+0.01*window/2+T2(1:(length(T2)-window+1));
% ind=crossing(cf);
% indt=timeuse(ind);
% indt(1:3)
%
% % find the first peak of V2
% der=Y2(2:end,8)-Y2(1:end-1,8);
% indp=crossing(der);
% indt_peak=T2(indp)+Ts;
```

```
% indt_peak(1:2)


%% color figures (Model R3) q1=1e-8,q2=1e-8

Itv=[1:1:14];

% the data is based on both model solution and MC coefficient
% FstPeak separates Phase 1 and 2; L1 separates Phase 2 and 3; L2 separates Phase 3
and 4;
L1=[inf,21.43,19.80,18.80,17.79,16.79,15.79,14.79,14,14,14,14,14,14]-14;
L2=[NaN,29.11,27.34,26.41,24.74,23.08,21.52,20.13,19.00,18.18,17.68,17.40,17.27,17.20]
-14;
FstPeak=[16.11,15.13,14,14,14,14,14,14,14,14,14,14,14,14]-14;

figure(3)
subplot(4,4,16);
h1=plot(L1,Itv,'-k','linewidth',1);
hold on
h2=plot(L2,Itv,'-k','linewidth',1);
h3=plot(FstPeak,Itv,'-k','linewidth',1);
set(gcf,'Position',[10 200 500 400])
set(gca,'FontSize',12,'xtick',0:2:14,'ytick',1:3:10)
set(gca,'YDir','reverse')
axis([0 13 1 10])
% ylabel('inter-exposure interval (day)','fontsize',16)
xlabel('time (day)','fontsize',16)

line([-1,100],[2,2],'LineStyle','--','Color','k','linewidth',1)
line([-1,100],[3,3],'LineStyle','--','Color','k','linewidth',1)
line([-1,100],[9,9],'LineStyle','--','Color','k','linewidth',1)

indfst=sum(heaviside(FstPeak-0.01))+1;

P1x=[0,FstPeak(1:indfst),0];
P1y=[1,1:indfst,1];

indl1=sum(heaviside(L1(2:end)-0.01))+2;

P2x=[fliplr(FstPeak(1:indfst)),13,13,L1(2:indl1),0];
P2y=[indfst:-1:1,1,2,2:indl1,indfst];

P3x=[fliplr(L1(2:indl1)),L2(2:10),0,0];
P3y=[indl1:-1:2,2:10,10,indl1];

P4x=[13,13,L2(2:10),13];
P4y=[10,2,2:10,10];

hold on
patch(P1x,P1y,[0 0 0],'facealpha',0.2)
patch(P2x,P2y,[1 0 0],'facealpha',0.3)
patch(P3x,P3y,[0 1 0],'facealpha',0.3)
patch(P4x,P4y,[0 162/255 1],'facealpha',0.3)

text(8,8,'q_1 = 1\times 10^{-8}')
text(8,9,'q_2 = 1\times 10^{-8}')

%% color figures (Model R3) q1=1e-8,q2=1e-7

Itv=[1:1:14];

L1=[inf,21.43,19.80,18.80,17.79,16.79,15.79,14.79,14,14,14,14,14,14]-14;
L2=[NaN,29.07,27.29,26.37,24.70,23.04,21.48,20.09,18.96,18.14,17.64,17.36,17.23,17.16]
-14;
FstPeak=[16.11,15.13,14,14,14,14,14,14,14,14,14,14,14,14]-14;

figure(3)
subplot(4,4,15);
h1=plot(L1,Itv,'-k','linewidth',1);
hold on
h2=plot(L2,Itv,'-k','linewidth',1);
h3=plot(FstPeak,Itv,'-k','linewidth',1);
set(gcf,'Position',[10 200 500 400])
set(gca,'FontSize',12,'xtick',0:2:14,'ytick',1:3:10)
```

```
set(gca,'YDir','reverse')
axis([0 13 1 10])
% ylabel('inter-exposure interval (day)','fontsize',16)
% xlabel('time (day)','fontsize',16)

line([-1,100],[2,2],'LineStyle','--','Color','k','linewidth',1)
line([-1,100],[3,3],'LineStyle','--','Color','k','linewidth',1)
line([-1,100],[9,9],'LineStyle','--','Color','k','linewidth',1)


indfst=sum(heaviside(FstPeak-0.01))+1;

P1x=[0,FstPeak(1:indfst),0];
P1y=[1,1:indfst,1];

indl1=sum(heaviside(L1(2:end)-0.01))+2;

P2x=[fliplr(FstPeak(1:indfst)),13,13,L1(2:indl1),0];
P2y=[indfst:-1:1,1,2,2:indl1,indfst];

P3x=[fliplr(L1(2:indl1)),L2(2:10),0,0];
P3y=[indl1:-1:2,2:10,10,indl1];

P4x=[13,13,L2(2:10),13];
P4y=[10,2,2:10,10];

hold on
patch(P1x,P1y,[0 0 0],'facealpha',0.2)
patch(P2x,P2y,[1 0 0],'facealpha',0.3)
patch(P3x,P3y,[0 1 0],'facealpha',0.3)
patch(P4x,P4y,[0 162/255 1],'facealpha',0.3)

text(8,8,'q_1 = 1\times 10^{-8}')
text(8,9,'q_2 = 1\times 10^{-7}')

%% color figures (Model R3) q1=1e-8,q2=5e-6

Itv=[1:1:14];

L1=[inf,21.42,19.80,18.80,17.79,16.79,15.79,14.79,14,14,14,14,14,14]-14;
L2=[NaN,27.68,26.70,25.78,24.10,22.44,20.87,19.47,18.33,17.52,17.03,16.76,16.63,16.57]
-14;
FstPeak=[16.09,15.13,14,14,14,14,14,14,14,14,14,14,14,14]-14;

figure(3)
subplot(4,4,14);
h1=plot(L1,Itv,'-k','linewidth',1);
hold on
h2=plot(L2,Itv,'-k','linewidth',1);
h3=plot(FstPeak,Itv,'-k','linewidth',1);
set(gcf,'Position',[10 200 500 400])
set(gca,'FontSize',12,'xtick',0:2:14,'ytick',1:3:10)
set(gca,'YDir','reverse')
axis([0 13 1 10])
% ylabel('inter-exposure interval (day)','fontsize',16)
% xlabel('time (day)','fontsize',16)

line([-1,100],[2,2],'LineStyle','--','Color','k','linewidth',1)
line([-1,100],[3,3],'LineStyle','--','Color','k','linewidth',1)
line([-1,100],[9,9],'LineStyle','--','Color','k','linewidth',1)


indfst=sum(heaviside(FstPeak-0.01))+1;

P1x=[0,FstPeak(1:indfst),0];
P1y=[1,1:indfst,1];

indl1=sum(heaviside(L1(2:end)-0.01))+2;

P2x=[fliplr(FstPeak(1:indfst)),13,13,L1(2:indl1),0];
P2y=[indfst:-1:1,1,2,2:indl1,indfst];

P3x=[fliplr(L1(2:indl1)),L2(2:10),0,0];
P3y=[indl1:-1:2,2:10,10,indl1];
```

```
P4x=[13,13,L2(2:10),13];
P4y=[10,2,2:10,10];

hold on
patch(P1x,P1y,[0 0 0],'facealpha',0.2)
patch(P2x,P2y,[1 0 0],'facealpha',0.3)
patch(P3x,P3y,[0 1 0],'facealpha',0.3)
patch(P4x,P4y,[0 162/255 1],'facealpha',0.3)

text(8,8,'q_1 = 1\times 10^{-8}')
text(8,9,'q_2 = 5\times 10^{-6}')

%% color figures (Model R3) q1=1e-8,q2=5e-5

Itv=[1:1:14];

L1=[inf,21.31,19.80,18.80,17.79,16.79,15.79,14.79,14,14,14,14,14,14]-14;
L2=[NaN,27.82,26.22,25.30,23.62,21.94,20.36,18.95,17.80,17.00,16.53,16.28,16.16,16.10]
-14;
FstPeak=[15.98,15.13,14,14,14,14,14,14,14,14,14,14,14,14]-14;

figure(3)
subplot(4,4,13);
h1=plot(L1,Itv,'-k','linewidth',1);
hold on
h2=plot(L2,Itv,'-k','linewidth',1);
h3=plot(FstPeak,Itv,'-k','linewidth',1);
set(gcf,'Position',[10 200 500 400])
set(gca,'FontSize',12,'xtick',0:2:14,'ytick',1:3:10)
set(gca,'YDir','reverse')
axis([0 13 1 10])
% ylabel('inter-exposure interval (day)','fontsize',16)
% xlabel('time (day)','fontsize',16)

line([-1,100],[2,2],'LineStyle','--','Color','k','linewidth',1)
line([-1,100],[3,3],'LineStyle','--','Color','k','linewidth',1)
line([-1,100],[9,9],'LineStyle','--','Color','k','linewidth',1)


indfst=sum(heaviside(FstPeak-0.01))+1;

P1x=[0,FstPeak(1:indfst),0];
P1y=[1,1:indfst,1];

indl1=sum(heaviside(L1(2:end)-0.01))+2;

P2x=[fliplr(FstPeak(1:indfst)),13,13,L1(2:indl1),0];
P2y=[indfst:-1:1,1,2,2:indl1,indfst];

P3x=[fliplr(L1(2:indl1)),L2(2:10),0,0];
P3y=[indl1:-1:2,2:10,10,indl1];

P4x=[13,13,L2(2:10),13];
P4y=[10,2,2:10,10];

hold on
patch(P1x,P1y,[0 0 0],'facealpha',0.2)
patch(P2x,P2y,[1 0 0],'facealpha',0.3)
patch(P3x,P3y,[0 1 0],'facealpha',0.3)
patch(P4x,P4y,[0 162/255 1],'facealpha',0.3)

text(8,8,'q_1 = 1\times 10^{-8}')
text(8,9,'q_2 = 5\times 10^{-5}')

%% color figures (Model R3) q1=1e-7,q2=1e-8

Itv=[1:1:14];

L1=[inf,18.41,17.09,16.09,15.09,14,14,14,14,14,14,14,14,14]-14;
L2=[NaN,24.07,23.33,22.01,20.51,19.29,18.38,17.79,17.46,17.30,17.22,17.18,17.16,17.16]
-14;
FstPeak=[16.07,15.09,14,14,14,14,14,14,14,14,14,14,14,14]-14;
```

```
figure(3)
subplot(4,4,12);
h1=plot(L1,Itv,'-k','linewidth',1);
hold on
h2=plot(L2,Itv,'-k','linewidth',1);
h3=plot(FstPeak,Itv,'-k','linewidth',1);
set(gcf,'Position',[10 200 500 400])
set(gca,'FontSize',12,'xtick',0:2:14,'ytick',1:3:10)
set(gca,'YDir','reverse')
axis([0 13 1 10])
% ylabel('inter-exposure interval (day)','fontsize',16)
% xlabel('time (day)','fontsize',16)

line([-1,100],[2,2],'LineStyle','--','Color','k','linewidth',1)
line([-1,100],[3,3],'LineStyle','--','Color','k','linewidth',1)
line([-1,100],[6,6],'LineStyle','--','Color','k','linewidth',1)


indfst=sum(heaviside(FstPeak-0.01))+1;

P1x=[0,FstPeak(1:indfst),0];
P1y=[1,1:indfst,1];

indl1=sum(heaviside(L1(2:end)-0.01))+2;

P2x=[fliplr(FstPeak(1:indfst)),13,13,L1(2:indl1),0];
P2y=[indfst:-1:1,1,2,2:indl1,indfst];

P3x=[fliplr(L1(2:indl1)),L2(2:10),0,0];
P3y=[indl1:-1:2,2:10,10,indl1];

P4x=[13,13,L2(2:10),13];
P4y=[10,2,2:10,10];

hold on
patch(P1x,P1y,[0 0 0],'facealpha',0.2)
patch(P2x,P2y,[1 0 0],'facealpha',0.3)
patch(P3x,P3y,[0 1 0],'facealpha',0.3)
patch(P4x,P4y,[0 162/255 1],'facealpha',0.3)

text(8,8,'q_1 = 1\times 10^{-7}')
text(8,9,'q_2 = 1\times 10^{-8}')

%% color figures (Model R3) q1=1e-7,q2=1e-7

Itv=[1:1:14];

L1=[Inf,18.41,17.09,16.09,15.09,14,14,14,14,14,14,14,14,14]-14;
L2=[NaN,24.03,23.29,21.97,20.47,19.24,18.34,17.75,17.42,17.26,17.18,17.14,17.12,17.11]
-14;
FstPeak=[16.07,15.09,14,14,14,14,14,14,14,14,14,14,14,14]-14;

figure(3)
subplot(4,4,11);
h1=plot(L1,Itv,'-k','linewidth',1);
hold on
h2=plot(L2,Itv,'-k','linewidth',1);
h3=plot(FstPeak,Itv,'-k','linewidth',1);
set(gcf,'Position',[10 200 500 400])
set(gca,'FontSize',12,'xtick',0:2:14,'ytick',1:3:10)
set(gca,'YDir','reverse')
axis([0 13 1 10])
% ylabel('inter-exposure interval (day)','fontsize',16)
% xlabel('time (day)','fontsize',16)

line([-1,100],[2,2],'LineStyle','--','Color','k','linewidth',1)
line([-1,100],[3,3],'LineStyle','--','Color','k','linewidth',1)
line([-1,100],[6,6],'LineStyle','--','Color','k','linewidth',1)


indfst=sum(heaviside(FstPeak-0.01))+1;

P1x=[0,FstPeak(1:indfst),0];
P1y=[1,1:indfst,1];
```

```
indl1=sum(heaviside(L1(2:end)-0.01))+2;

P2x=[fliplr(FstPeak(1:indfst)),13,13,L1(2:indl1),0];
P2y=[indfst:-1:1,1,2,2:indl1,indfst];

P3x=[fliplr(L1(2:indl1)),L2(2:10),0,0];
P3y=[indl1:-1:2,2:10,10,indl1];

P4x=[13,13,L2(2:10),13];
P4y=[10,2,2:10,10];

hold on
patch(P1x,P1y,[0 0 0],'facealpha',0.2)
patch(P2x,P2y,[1 0 0],'facealpha',0.3)
patch(P3x,P3y,[0 1 0],'facealpha',0.3)
patch(P4x,P4y,[0 162/255 1],'facealpha',0.3)

text(8,8,'q_1 = 1\times 10^{-7}')
text(8,9,'q_2 = 1\times 10^{-7}')

%% color figures (Model R3) q1=1e-7,q2=5e-6

Itv=[1:1:14];

L1=[inf,18.41,17.10,16.09,15.09,14,14,14,14,14,14,14,14,14]-14;
L2=[NaN,23.43,22.68,21.37,19.86,18.63,17.72,17.14,16.82,16.66,16.59,16.55,16.54,16.53]
-14;
FstPeak=[16.05,15.09,14,14,14,14,14,14,14,14,14,14,14,14]-14;

figure(3)
subplot(4,4,10);
h1=plot(L1,Itv,'-k','linewidth',1);
hold on
h2=plot(L2,Itv,'-k','linewidth',1);
h3=plot(FstPeak,Itv,'-k','linewidth',1);
set(gcf,'Position',[10 200 500 400])
set(gca,'FontSize',12,'xtick',0:2:14,'ytick',1:3:10)
set(gca,'YDir','reverse')
axis([0 13 1 10])
% ylabel('inter-exposure interval (day)','fontsize',16)
% xlabel('time (day)','fontsize',16)

line([-1,100],[2,2],'LineStyle','--','Color','k','linewidth',1)
line([-1,100],[3,3],'LineStyle','--','Color','k','linewidth',1)
line([-1,100],[6,6],'LineStyle','--','Color','k','linewidth',1)


indfst=sum(heaviside(FstPeak-0.01))+1;

P1x=[0,FstPeak(1:indfst),0];
P1y=[1,1:indfst,1];

indl1=sum(heaviside(L1(2:end)-0.01))+2;

P2x=[fliplr(FstPeak(1:indfst)),13,13,L1(2:indl1),0];
P2y=[indfst:-1:1,1,2,2:indl1,indfst];

P3x=[fliplr(L1(2:indl1)),L2(2:10),0,0];
P3y=[indl1:-1:2,2:10,10,indl1];

P4x=[13,13,L2(2:10),13];
P4y=[10,2,2:10,10];

hold on
patch(P1x,P1y,[0 0 0],'facealpha',0.2)
patch(P2x,P2y,[1 0 0],'facealpha',0.3)
patch(P3x,P3y,[0 1 0],'facealpha',0.3)
patch(P4x,P4y,[0 162/255 1],'facealpha',0.3)

text(8,8,'q_1 = 1\times 10^{-7}')
text(8,9,'q_2 = 5\times 10^{-6}')

%% color figures (Model R3) q1=1e-7,q2=5e-5
```

```
Itv=[1:1:14];

L1=[inf,18.37,17.09,16.09,15.09,14,14,14,14,14,14,14,14,14]-14;
L2=[NaN,22.88,22.19,20.86,19.33,18.09,17.19,16.63,16.33,16.18,16.11,16.08,16.06,16.06]
-14;
FstPeak=[15.95,15.09,14,14,14,14,14,14,14,14,14,14,14,14]-14;

figure(3)
subplot(4,4,9);
h1=plot(L1,Itv,'-k','linewidth',1);
hold on
h2=plot(L2,Itv,'-k','linewidth',1);
h3=plot(FstPeak,Itv,'-k','linewidth',1);
set(gcf,'Position',[10 200 500 400])
set(gca,'FontSize',12,'xtick',0:2:14,'ytick',1:3:10)
set(gca,'YDir','reverse')
axis([0 13 1 10])
% ylabel('inter-exposure interval (day)','fontsize',16)
% xlabel('time (day)','fontsize',16)

line([-1,100],[2,2],'LineStyle','--','Color','k','linewidth',1)
line([-1,100],[3,3],'LineStyle','--','Color','k','linewidth',1)
line([-1,100],[6,6],'LineStyle','--','Color','k','linewidth',1)


indfst=sum(heaviside(FstPeak-0.01))+1;

P1x=[0,FstPeak(1:indfst),0];
P1y=[1,1:indfst,1];

indl1=sum(heaviside(L1(2:end)-0.01))+2;

P2x=[fliplr(FstPeak(1:indfst)),13,13,L1(2:indl1),0];
P2y=[indfst:-1:1,1,2,2:indl1,indfst];

P3x=[fliplr(L1(2:indl1)),L2(2:10),0,0];
P3y=[indl1:-1:2,2:10,10,indl1];

P4x=[13,13,L2(2:10),13];
P4y=[10,2,2:10,10];

hold on
patch(P1x,P1y,[0 0 0],'facealpha',0.2)
patch(P2x,P2y,[1 0 0],'facealpha',0.3)
patch(P3x,P3y,[0 1 0],'facealpha',0.3)
patch(P4x,P4y,[0 162/255 1],'facealpha',0.3)

text(8,8,'q_1 = 1\times 10^{-7}')
text(8,9,'q_2 = 5\times 10^{-5}')

%% color figures (Model R3) q1=5e-6,q2=1e-8

Itv=[1:1:14];

L1=[16.53,15.29,14.28,14,14,14,14,14,14,14,14,14,14,14]-14;
L2=[19.62,19.22,18.36,17.54,17.24,17.17,17.16,17.15,17.15,17.15,17.15,17.15,17.15,17.1
5]-14;
FstPeak=[15.52,14.54,14,14,14,14,14,14,14,14,14,14,14,14]-14;

figure(3)
subplot(4,4,8);
h1=plot(L1,Itv,'-k','linewidth',1);
hold on
h2=plot(L2,Itv,'-k','linewidth',1);
h3=plot(FstPeak,Itv,'-k','linewidth',1);
set(gcf,'Position',[10 200 500 400])
set(gca,'FontSize',12,'xtick',0:2:14,'ytick',1:3:10)
set(gca,'YDir','reverse')
axis([0 13 1 10])
% ylabel('inter-exposure interval (day)','fontsize',16)
% xlabel('time (day)','fontsize',16)

line([-1,100],[3,3],'LineStyle','--','Color','k','linewidth',1)
```

```
line([-1,100],[4,4],'LineStyle','--','Color','k','linewidth',1)

indfst=sum(heaviside(FstPeak-0.01))+1;

P1x=[0,FstPeak(1:indfst),0];
P1y=[1,1:indfst,1];

indl1=sum(heaviside(L1-0.01))+1;

P2x=[fliplr(FstPeak(1:indfst)),L1(1:indl1),0];
P2y=[indfst:-1:1,1:indl1,indfst];

P3x=[fliplr(L1(1:indl1)),L2(1:10),0,0];
P3y=[indl1:-1:1,1:10,10,indl1];

P4x=[13,13,L2(1:10),13];
P4y=[10,1,1:10,10];

hold on
patch(P1x,P1y,[0 0 0],'facealpha',0.2)
patch(P2x,P2y,[1 0 0],'facealpha',0.3)
patch(P3x,P3y,[0 1 0],'facealpha',0.3)
patch(P4x,P4y,[0 162/255 1],'facealpha',0.3)

text(8,8,'q_1 = 5\times 10^{-6}')
text(8,9,'q_2 = 1\times 10^{-8}')

%% color figures (Model R3) q1=5e-6,q2=1e-7

Itv=[1:1:14];

L1=[16.53,15.29,14.29,14,14,14,14,14,14,14,14,14,14,14]-14;
L2=[19.59,19.19,18.32,17.50,17.20,17.14,17.12,17.12,17.11,17.11,17.11,17.11,17.11,17.1
1]-14;
FstPeak=[15.52,14.54,14,14,14,14,14,14,14,14,14,14,14,14]-14;

figure(3)
subplot(4,4,7);
h1=plot(L1,Itv,'-k','linewidth',1);
hold on
h2=plot(L2,Itv,'-k','linewidth',1);
h3=plot(FstPeak,Itv,'-k','linewidth',1);
set(gcf,'Position',[10 200 500 400])
set(gca,'FontSize',12,'xtick',0:2:14,'ytick',1:3:10)
set(gca,'YDir','reverse')
axis([0 13 1 10])
% ylabel('inter-exposure interval (day)','fontsize',16)
% xlabel('time (day)','fontsize',16)

line([-1,100],[3,3],'LineStyle','--','Color','k','linewidth',1)
line([-1,100],[4,4],'LineStyle','--','Color','k','linewidth',1)

indfst=sum(heaviside(FstPeak-0.01))+1;

P1x=[0,FstPeak(1:indfst),0];
P1y=[1,1:indfst,1];

indl1=sum(heaviside(L1-0.01))+1;

P2x=[fliplr(FstPeak(1:indfst)),L1(1:indl1),0];
P2y=[indfst:-1:1,1:indl1,indfst];

P3x=[fliplr(L1(1:indl1)),L2(1:10),0,0];
P3y=[indl1:-1:1,1:10,10,indl1];

P4x=[13,13,L2(1:10),13];
P4y=[10,1,1:10,10];

hold on
patch(P1x,P1y,[0 0 0],'facealpha',0.2)
patch(P2x,P2y,[1 0 0],'facealpha',0.3)
patch(P3x,P3y,[0 1 0],'facealpha',0.3)
patch(P4x,P4y,[0 162/255 1],'facealpha',0.3)
```

```
text(8,8,'q_1 = 5\times 10^{-6}')
text(8,9,'q_2 = 1\times 10^{-7}')

%% color figures (Model R3) q1=5e-6,q2=5e-6

Itv=[1:1:14];

L1=[16.53,15.28,14.28,14,14,14,14,14,14,14,14,14,14,14]-14;
L2=[18.99,18.59,17.72,16.90,16.61,16.54,16.52,16.52,16.52,16.52,16.52,16.52,16.52,16.5
2]-14;
FstPeak=[15.52,14.54,14,14,14,14,14,14,14,14,14,14,14,14]-14;

figure(3)
subplot(4,4,6);
h1=plot(L1,Itv,'-k','linewidth',1);
hold on
h2=plot(L2,Itv,'-k','linewidth',1);
h3=plot(FstPeak,Itv,'-k','linewidth',1);
set(gcf,'Position',[10 200 500 400])
set(gca,'FontSize',12,'xtick',0:2:14,'ytick',1:3:10)
set(gca,'YDir','reverse')
axis([0 13 1 10])
% ylabel('inter-exposure interval (day)','fontsize',16)
% xlabel('time (day)','fontsize',16)

line([-1,100],[3,3],'LineStyle','--','Color','k','linewidth',1)
line([-1,100],[4,4],'LineStyle','--','Color','k','linewidth',1)

indfst=sum(heaviside(FstPeak-0.01))+1;

P1x=[0,FstPeak(1:indfst),0];
P1y=[1,1:indfst,1];

indl1=sum(heaviside(L1-0.01))+1;

P2x=[fliplr(FstPeak(1:indfst)),L1(1:indl1),0];
P2y=[indfst:-1:1,1:indl1,indfst];

P3x=[fliplr(L1(1:indl1)),L2(1:10),0,0];
P3y=[indl1:-1:1,1:10,10,indl1];

P4x=[13,13,L2(1:10),13];
P4y=[10,1,1:10,10];

hold on
patch(P1x,P1y,[0 0 0],'facealpha',0.2)
patch(P2x,P2y,[1 0 0],'facealpha',0.3)
patch(P3x,P3y,[0 1 0],'facealpha',0.3)
patch(P4x,P4y,[0 162/255 1],'facealpha',0.3)

text(8,8,'q_1 = 5\times 10^{-6}')
text(8,9,'q_2 = 5\times 10^{-6}')

%% color figures (Model R3) q1=5e-6,q2=5e-5

Itv=[1:1:14];

L1=[16.51,15.29,14.28,14,14,14,14,14,14,14,14,14,14,14]-14;
L2=[18.50,18.11,17.25,16.43,16.14,16.07,16.06,16.05,16.05,16.05,16.05,16.05,16.05,16.0
5]-14;
FstPeak=[15.51,14.54,14,14,14,14,14,14,14,14,14,14,14,14]-14;

figure(3)
subplot(4,4,5);
h1=plot(L1,Itv,'-k','linewidth',1);
hold on
h2=plot(L2,Itv,'-k','linewidth',1);
h3=plot(FstPeak,Itv,'-k','linewidth',1);
set(gcf,'Position',[10 200 500 400])
set(gca,'FontSize',12,'xtick',0:2:14,'ytick',1:3:10)
set(gca,'YDir','reverse')
axis([0 13 1 10])
% ylabel('inter-exposure interval (day)','fontsize',16)
% xlabel('time (day)','fontsize',16)
```

```
line([-1,100],[3,3],'LineStyle','--','Color','k','linewidth',1)
line([-1,100],[4,4],'LineStyle','--','Color','k','linewidth',1)

indfst=sum(heaviside(FstPeak-0.01))+1;

P1x=[0,FstPeak(1:indfst),0];
P1y=[1,1:indfst,1];

indl1=sum(heaviside(L1-0.01))+1;

P2x=[fliplr(FstPeak(1:indfst)),L1(1:indl1),0];
P2y=[indfst:-1:1,1:indl1,indfst];

P3x=[fliplr(L1(1:indl1)),L2(1:10),0,0];
P3y=[indl1:-1:1,1:10,10,indl1];

P4x=[13,13,L2(1:10),13];
P4y=[10,1,1:10,10];

hold on
patch(P1x,P1y,[0 0 0],'facealpha',0.2)
patch(P2x,P2y,[1 0 0],'facealpha',0.3)
patch(P3x,P3y,[0 1 0],'facealpha',0.3)
patch(P4x,P4y,[0 162/255 1],'facealpha',0.3)

text(8,8,'q_1 = 5\times 10^{-6}')
text(8,9,'q_2 = 5\times 10^{-5}')

%% color figures (Model R3) q1=5e-5,q2=1e-8

Itv=[1:1:14];

L1=[15.82,14.79,14,14,14,14,14,14,14,14,14,14,14,14]-14;
L2=[19.30,18.96,17.88,17.38,17.21,17.16,17.15,17.15,17.15,17.15,17.15,17.15,17.15,17.1
5]-14;
FstPeak=[15.08,14,14,14,14,14,14,14,14,14,14,14,14,14]-14;

figure(3)
subplot(4,4,4);
h1=plot(L1,Itv,'-k','linewidth',1);
hold on
h2=plot(L2,Itv,'-k','linewidth',1);
h3=plot(FstPeak,Itv,'-k','linewidth',1);
set(gcf,'Position',[10 200 500 400])
set(gca,'FontSize',12,'xtick',0:2:14,'ytick',1:3:10)
set(gca,'YDir','reverse')
axis([0 13 1 10])
% ylabel('inter-exposure interval (day)','fontsize',16)
% xlabel('time (day)','fontsize',16)

line([-1,100],[2,2],'LineStyle','--','Color','k','linewidth',1)
line([-1,100],[3,3],'LineStyle','--','Color','k','linewidth',1)

indfst=sum(heaviside(FstPeak-0.01))+1;

P1x=[0,FstPeak(1:indfst),0];
P1y=[1,1:indfst,1];

indl1=sum(heaviside(L1-0.01))+1;

P2x=[fliplr(FstPeak(1:indfst)),L1(1:indl1),0];
P2y=[indfst:-1:1,1:indl1,indfst];

P3x=[fliplr(L1(1:indl1)),L2(1:10),0,0];
P3y=[indl1:-1:1,1:10,10,indl1];

P4x=[13,13,L2(1:10),13];
P4y=[10,1,1:10,10];

hold on
patch(P1x,P1y,[0 0 0],'facealpha',0.2)
patch(P2x,P2y,[1 0 0],'facealpha',0.3)
patch(P3x,P3y,[0 1 0],'facealpha',0.3)
```

```
patch(P4x,P4y,[0 162/255 1],'facealpha',0.3)

text(8,8,'q_1 = 5\times 10^{-5}')
text(8,9,'q_2 = 1\times 10^{-8}')

%% color figures (Model R3) q1=5e-5,q2=1e-7

Itv=[1:1:14];

L1=[15.82,14.79,14,14,14,14,14,14,14,14,14,14,14,14]-14;
L2=[19.26,18.92,17.84,17.34,17.17,17.12,17.11,17.11,17.11,17.11,17.11,17.11,17.11,17.1
1]-14;
FstPeak=[15.08,14,14,14,14,14,14,14,14,14,14,14,14,14]-14;

figure(3)
subplot(4,4,3);
h1=plot(L1,Itv,'-k','linewidth',1);
hold on
h2=plot(L2,Itv,'-k','linewidth',1);
h3=plot(FstPeak,Itv,'-k','linewidth',1);
set(gcf,'Position',[10 200 500 400])
set(gca,'FontSize',12,'xtick',0:2:14,'ytick',1:3:10)
set(gca,'YDir','reverse')
axis([0 13 1 10])
% ylabel('inter-exposure interval (day)','fontsize',16)
% xlabel('time (day)','fontsize',16)

line([-1,100],[2,2],'LineStyle','--','Color','k','linewidth',1)
line([-1,100],[3,3],'LineStyle','--','Color','k','linewidth',1)

indfst=sum(heaviside(FstPeak-0.01))+1;

P1x=[0,FstPeak(1:indfst),0];
P1y=[1,1:indfst,1];

indl1=sum(heaviside(L1-0.01))+1;

P2x=[fliplr(FstPeak(1:indfst)),L1(1:indl1),0];
P2y=[indfst:-1:1,1:indl1,indfst];

P3x=[fliplr(L1(1:indl1)),L2(1:10),0,0];
P3y=[indl1:-1:1,1:10,10,indl1];

P4x=[13,13,L2(1:10),13];
P4y=[10,1,1:10,10];

hold on
patch(P1x,P1y,[0 0 0],'facealpha',0.2)
patch(P2x,P2y,[1 0 0],'facealpha',0.3)
patch(P3x,P3y,[0 1 0],'facealpha',0.3)
patch(P4x,P4y,[0 162/255 1],'facealpha',0.3)

text(8,8,'q_1 = 5\times 10^{-5}')
text(8,9,'q_2 = 1\times 10^{-7}')

%% color figures (Model R3) q1=5e-5,q2=5e-6

Itv=[1:1:14];

L1=[15.82,14.79,14,14,14,14,14,14,14,14,14,14,14,14]-14;
L2=[18.65,18.32,17.25,16.75,16.58,16.53,16.52,16.52,16.52,16.52,16.52,16.52,16.52,16.5
2]-14;
FstPeak=[15.08,14,14,14,14,14,14,14,14,14,14,14,14,14]-14;

figure(3)
subplot(4,4,2);
h1=plot(L1,Itv,'-k','linewidth',1);
hold on
h2=plot(L2,Itv,'-k','linewidth',1);
h3=plot(FstPeak,Itv,'-k','linewidth',1);
set(gcf,'Position',[10 200 500 400])
set(gca,'FontSize',12,'xtick',0:2:14,'ytick',1:3:10)
set(gca,'YDir','reverse')
axis([0 13 1 10])
```

```
% ylabel('inter-exposure interval (day)','fontsize',16)
% xlabel('time (day)','fontsize',16)

line([-1,100],[2,2],'LineStyle','--','Color','k','linewidth',1)
line([-1,100],[3,3],'LineStyle','--','Color','k','linewidth',1)

indfst=sum(heaviside(FstPeak-0.01))+1;

P1x=[0,FstPeak(1:indfst),0];
P1y=[1,1:indfst,1];

indl1=sum(heaviside(L1-0.01))+1;

P2x=[fliplr(FstPeak(1:indfst)),L1(1:indl1),0];
P2y=[indfst:-1:1,1:indl1,indfst];

P3x=[fliplr(L1(1:indl1)),L2(1:10),0,0];
P3y=[indl1:-1:1,1:10,10,indl1];

P4x=[13,13,L2(1:10),13];
P4y=[10,1,1:10,10];

hold on
patch(P1x,P1y,[0 0 0],'facealpha',0.2)
patch(P2x,P2y,[1 0 0],'facealpha',0.3)
patch(P3x,P3y,[0 1 0],'facealpha',0.3)
patch(P4x,P4y,[0 162/255 1],'facealpha',0.3)

text(8,8,'q_1 = 5\times 10^{-5}')
text(8,9,'q_2 = 5\times 10^{-6}')

%% color figures (Model R3) q1=5e-5,q2=5e-5

Itv=[1:1:14];

L1=[15.82,14.79,14,14,14,14,14,14,14,14,14,14,14,14]-14;
L2=[18.16,17.85,16.77,16.28,16.11,16.07,16.05,16.05,16.05,16.05,16.05,16.05,16.05,16.0
5]-14;
FstPeak=[15.08,14,14,14,14,14,14,14,14,14,14,14,14,14]-14;

figure(3)
subplot(4,4,1);
h1=plot(L1,Itv,'-k','linewidth',1);
hold on
h2=plot(L2,Itv,'-k','linewidth',1);
h3=plot(FstPeak,Itv,'-k','linewidth',1);
set(gcf,'Position',[10 200 500 400])
set(gca,'FontSize',12,'xtick',0:2:14,'ytick',1:3:10)
set(gca,'YDir','reverse')
axis([0 13 1 10])
ylabel('inter-exposure interval (day)','fontsize',16)
% xlabel('time (day)','fontsize',16)

line([-1,100],[2,2],'LineStyle','--','Color','k','linewidth',1)
line([-1,100],[3,3],'LineStyle','--','Color','k','linewidth',1)

indfst=sum(heaviside(FstPeak-0.01))+1;

P1x=[0,FstPeak(1:indfst),0];
P1y=[1,1:indfst,1];

indl1=sum(heaviside(L1-0.01))+1;

P2x=[fliplr(FstPeak(1:indfst)),L1(1:indl1),0];
P2y=[indfst:-1:1,1:indl1,indfst];

P3x=[fliplr(L1(1:indl1)),L2(1:10),0,0];
P3y=[indl1:-1:1,1:10,10,indl1];

P4x=[13,13,L2(1:10),13];
P4y=[10,1,1:10,10];

hold on
patch(P1x,P1y,[0 0 0],'facealpha',0.2)
```

```
patch(P2x,P2y,[1 0 0],'facealpha',0.3)
patch(P3x,P3y,[0 1 0],'facealpha',0.3)
patch(P4x,P4y,[0 162/255 1],'facealpha',0.3)

text(8,8,'q_1 = 5\times 10^{-5}')
text(8,9,'q_2 = 5\times 10^{-5}')

%%
set(gcf,'Position',[10 200 800 750])
```

7. Code for generating Fig. S13

```
%% color figures (Model R2 varying s; s1=10,s2=1,q1=5e-7,q2=5e-6)

Itv=[1:1:10];

L1ltoh=[15.33,14,14,14,14,14,14,14,14,14]-14;
L2ltoh=[16.74,17.02,16.95,16.77,16.66,16.57,16.49,16.44,16.41,16.41]-14;
FstPeakltoh=[NaN,NaN,NaN,NaN,NaN,NaN,NaN,NaN,NaN,NaN]-14;

figure
h1=plot(L1ltoh,Itv,'-k','linewidth',1);
hold on
h2=plot(L2ltoh,Itv,'-k','linewidth',1);
h3=plot(FstPeakltoh,Itv,'-k','linewidth',1);
set(gcf,'Position',[10 200 500 400])
set(gca,'FontSize',15,'xtick',0:2:14,'ytick',1:1:9)
set(gca,'YDir','reverse')
axis([0 8 1 9])
ylabel('re-infection interval (day)','fontsize',16)
xlabel('time (day)','fontsize',16)

line([-1,100],[2,2],'LineStyle','--','Color','k','linewidth',1)

P1x=[14,15.33,14,14]-14;
P1y=[1,1,2,1];

P3x=[14,15.33,16.74,17.02,16.95,16.77,16.66,16.57,16.49,16.44,16.41,14,14]-14;
P3y=[2,1,1,2,3,4,5,6,7,8,9,9,2];

P4x=[16.74,17.02,16.95,16.77,16.66,16.57,16.49,16.44,16.41,22,22,16.74]-14;
P4y=[1,2,3,4,5,6,7,8,9,9,1,1];

hold on
patch(P1x,P1y,[0 0 0],'facealpha',0.2)
patch(P3x,P3y,[0 1 0],'facealpha',0.3)
patch(P4x,P4y,[0 162/255 1],'facealpha',0.3)

%% color figures (Model R2 varying s; s1=1,s2=10,q1=5e-6,q2=5e-7)

Itv=[1:1:10];

L1htol=[20.80,19.79,18.79,17.79,16.77,15.79,14.79,14,14,14]-14;
L2htol=[26.33,25.79,24.37,22.87,21.41,20.03,18.71,17.52,16.74,16.47]-14;
FstPeakhtol=[14.70,14,14,14,14,14,14,14,14,14]-14;

figure
h1=plot(L1htol,Itv,'-k','linewidth',1);
hold on
h2=plot(L2htol,Itv,'-k','linewidth',1);
h3=plot(FstPeakhtol,Itv,'-k','linewidth',1);
set(gcf,'Position',[10 200 500 400])
set(gca,'FontSize',15,'xtick',0:2:14,'ytick',1:1:9)
set(gca,'YDir','reverse')
axis([0 13 1 9])
ylabel('re-infection interval (day)','fontsize',16)
xlabel('time (day)','fontsize',16)

line([-1,100],[2,2],'LineStyle','--','Color','k','linewidth',1)
line([-1,100],[8,8],'LineStyle','--','Color','k','linewidth',1)


P1x=[14,14.70,14,14]-14;
P1y=[1,1,2,1];

P2x=[14,14.70,20.80,19.79,18.79,17.79,16.77,15.79,14.79,14,14]-14;
P2y=[2,1,1,2,3,4,5,6,7,8,2];

P3x=[14,14.79,15.79,16.77,17.79,18.79,19.79,20.80,26.33,25.79,24.37,22.87,21.41,20.03,
18.71,17.52,16.74,14,14]-14;
P3y=[8,7,6,5,4,3,2,1,1,2,3,4,5,6,7,8,9,9,8];

P4x=[26.33,25.79,24.37,22.87,21.41,20.03,18.71,17.52,16.74,27,27,26.33]-14;
```

```
P4y=[1,2,3,4,5,6,7,8,9,9,1,1];

hold on
patch(P1x,P1y,[0 0 0],'facealpha',0.2)
patch(P2x,P2y,[1 0 0],'facealpha',0.3)
patch(P3x,P3y,[0 1 0],'facealpha',0.3)
patch(P4x,P4y,[0 162/255 1],'facealpha',0.3)
```

8. Code for generating Fig. S14

```
%% color figures (Model 3 varying kappa; kappa1=15,kappa2=3,q1=1e-6,q2=5e-6)

Itv=[1:1:10];

L1ltoh=[15.49,14,14,14,14,14,14,14,14,14]-14;
L2ltoh=[16.90,17.03,16.85,16.65,16.57,16.54,16.53,16.53,16.53,16.53]-14;
FstPeakltoh=[NaN,NaN,NaN,NaN,NaN,NaN,NaN,NaN,NaN,NaN]-14;

figure(3)
h1=plot(L1ltoh,Itv,'-k','linewidth',1);
hold on
h2=plot(L2ltoh,Itv,'-k','linewidth',1);
h3=plot(FstPeakltoh,Itv,'-k','linewidth',1);
set(gcf,'Position',[10 200 500 400])
set(gca,'FontSize',15,'xtick',0:2:14,'ytick',1:1:9)
set(gca,'YDir','reverse')
axis([0 8 1 9])
ylabel('re-infection interval (day)','fontsize',16)
xlabel('time (day)','fontsize',16)

line([-1,100],[2,2],'LineStyle','--','Color','k','linewidth',1)

P1x=[14,15.49,14,14]-14;
P1y=[1,1,2,1];

P3x=[14,15.49,16.90,17.03,16.85,16.65,16.57,16.54,16.53,16.53,16.53,14,14]-14;
P3y=[2,1,1,2,3,4,5,6,7,8,9,9,2];

P4x=[16.90,17.03,16.85,16.65,16.57,16.54,16.53,16.53,16.53,27,27,16.90]-14;
P4y=[1,2,3,4,5,6,7,8,9,9,1,1];

hold on
patch(P1x,P1y,[0 0 0],'facealpha',0.2)
patch(P3x,P3y,[0 1 0],'facealpha',0.3)
patch(P4x,P4y,[0 162/255 1],'facealpha',0.3)

%% color figures (Model 3 varying kappa; kappa1=3,kappa2=15,q1=5e-6,q2=1e-6)

Itv=[1:1:10];

L1htol=[17.76,16.71,15.71,14.71,14,14,14,14,14,14]-14;
L2htol=[26.78,25.75,21.85,18.20,16.86,16.58,16.53,16.52,16.52,16.52]-14;
FstPeakhtol=[15.03,14,14,14,14,14,14,14,14,14]-14;

figure(3)
h1=plot(L1htol,Itv,'-k','linewidth',1);
hold on
h2=plot(L2htol,Itv,'-k','linewidth',1);
h3=plot(FstPeakhtol,Itv,'-k','linewidth',1);
set(gcf,'Position',[10 200 500 400])
set(gca,'FontSize',15,'xtick',0:2:14,'ytick',1:1:9)
set(gca,'YDir','reverse')
axis([0 13 1 9])
ylabel('re-infection interval (day)','fontsize',16)
xlabel('time (day)','fontsize',16)

line([-1,100],[2,2],'LineStyle','--','Color','k','linewidth',1)
line([-1,100],[5,5],'LineStyle','--','Color','k','linewidth',1)


P1x=[14,15.03,14,14]-14;
P1y=[1,1,2,1];

P2x=[14,15.03,17.76,16.71,15.71,14.71,14,14]-14;
P2y=[2,1,1,2,3,4,5,2];

P3x=[14,14.71,15.71,16.71,17.76,26.78,25.75,21.85,18.20,16.86,16.58,16.53,16.52,16.52,
14,14]-14;
P3y=[5,4,3,2,1,1,2,3,4,5,6,7,8,9,9,5];

P4x=[26.78,25.75,21.85,18.20,16.86,16.58,16.53,16.52,16.52,27,27,26.78]-14;
P4y=[1,2,3,4,5,6,7,8,9,9,1,1];
```

```
hold on
patch(P1x,P1y,[0 0 0],'facealpha',0.2)
patch(P2x,P2y,[1 0 0],'facealpha',0.3)
patch(P3x,P3y,[0 1 0],'facealpha',0.3)
patch(P4x,P4y,[0 162/255 1],'facealpha',0.3)
```
