# Supplementary figures and images for: Innate Immunity and the Inter-exposure Interval Determine the Dynamics of Secondary Influenza Virus Infection and Explain Observed Viral Hierarchies
Source: PLoS Comput Biol. 2015 Aug 18;11(8):e1004334. doi: 10.1371/journal.pcbi.1004334 (PMC4540579; doi:10.1371/journal.pcbi.1004334)

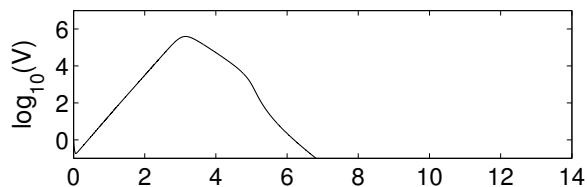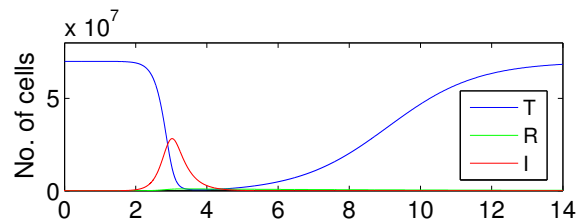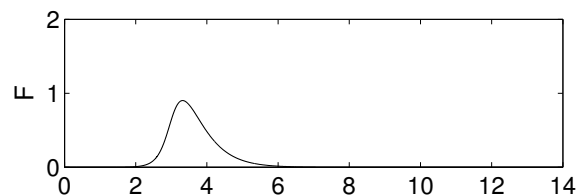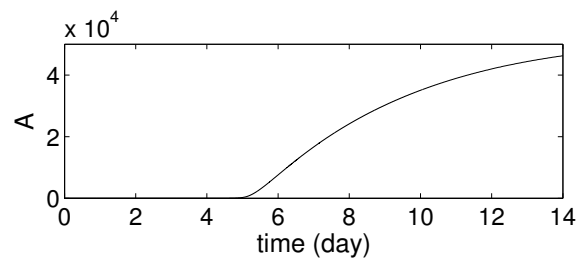

(A)  $q = 10^{-7}$

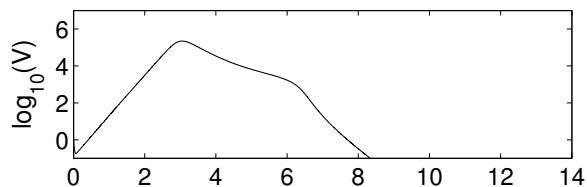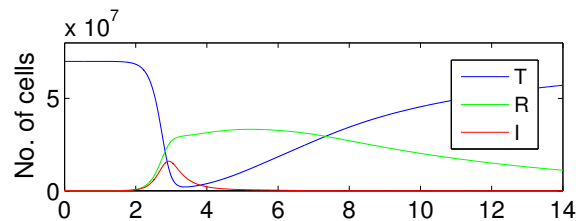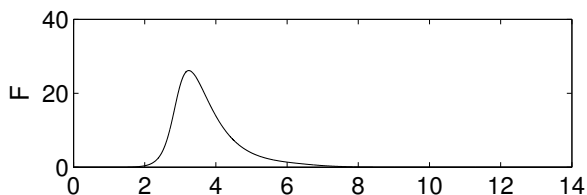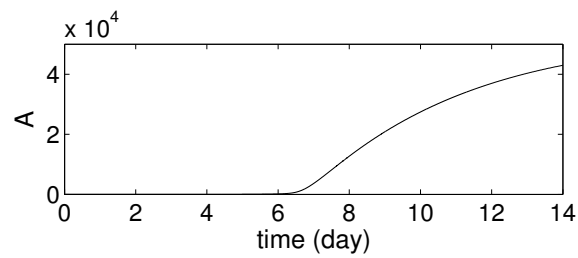

(B)  $q = 5 \times 10^{-6}$

Supplement: S1 Fig — Solution of Model 1 for two different IFN production rates, q = 10−7 and q = 5 × 10^−6^, under the initial condition of V(0) = 1, T(0) = C t and zeros for all other variables. (PDF) [file pcbi.1004334.s003.pdf]

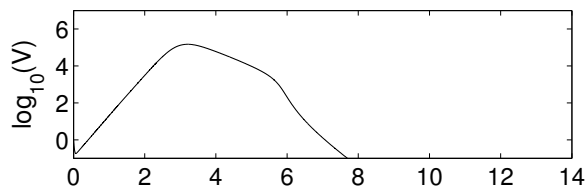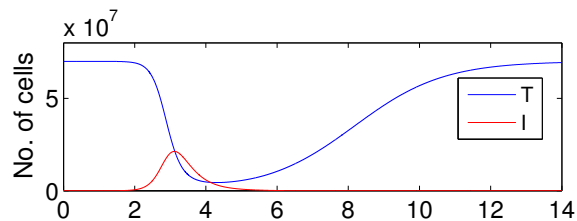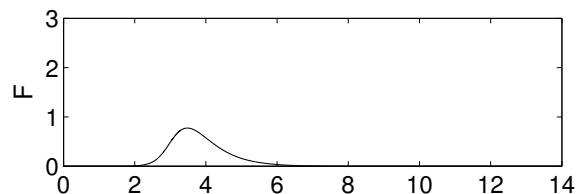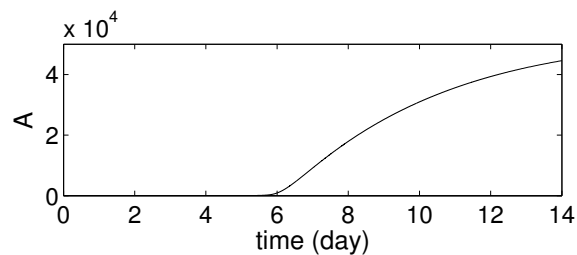

(A)  $q = 10^{-7}$

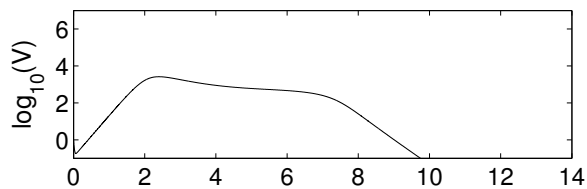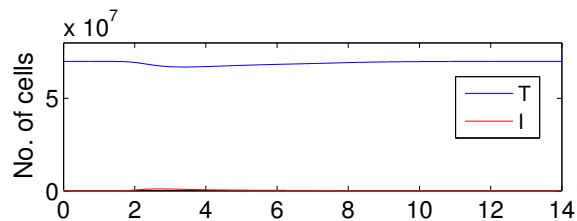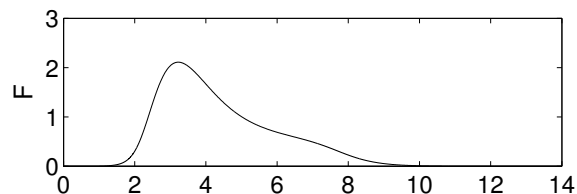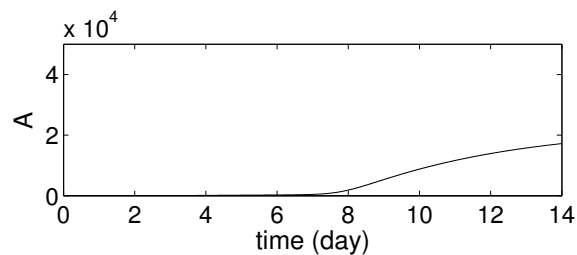

(B)  $q = 5 \times 10^{-6}$

Supplement: S2 Fig — Solutions of Model 2 for two different IFN production rates, q = 10−7 and q = 5 × 10^−6^, under the initial condition of V(0) = 1, T(0) = C t and zeros for all other variables. We set s = 1 here, which will be treated as a benchmark value for later comparisons. (PDF) [file pcbi.1004334.s004.pdf]

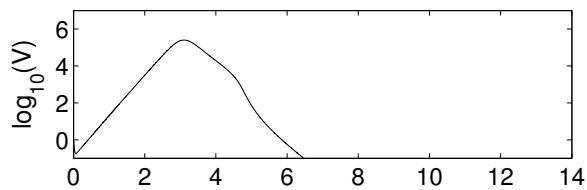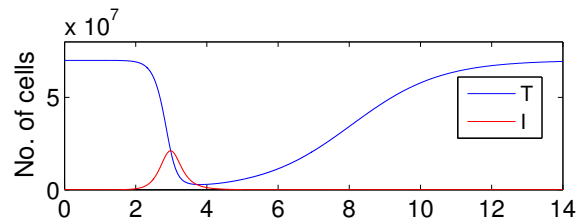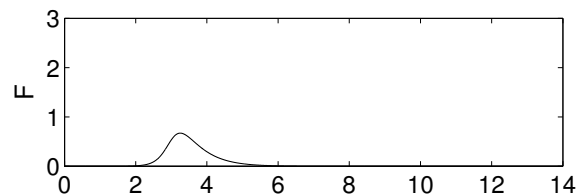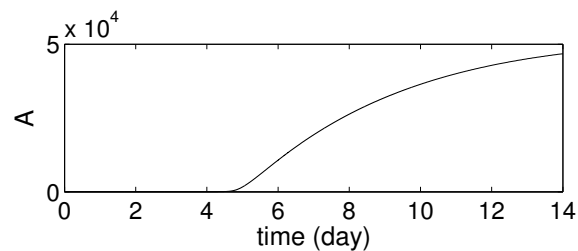

(A)  $q = 10^{-7}$

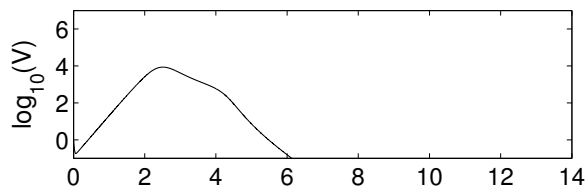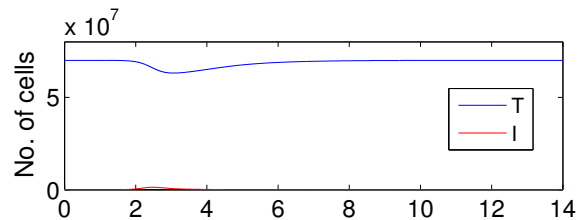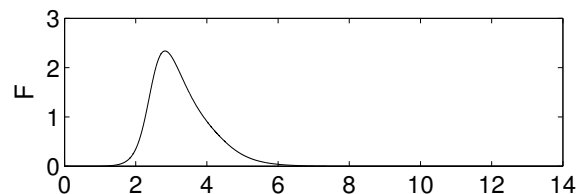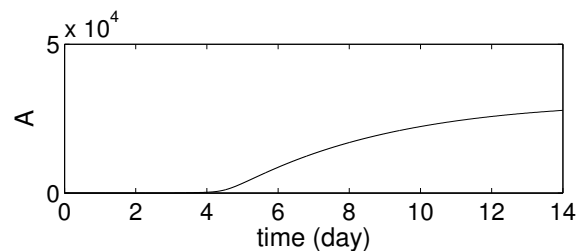

(B)  $q = 5 \times 10^{-6}$

Supplement: S3 Fig — Solutions of Model 3 for two different IFN production rates, q = 10−7 and q = 5 × 10^−6^, under the initial condition of V(0) = 1, T(0) = C t and zeros for all other variables. We use a benchmark value of κ = 3, which lies around the middle of the range estimated from the paper by Pawelek et al. (see reference 28 in the main text) (PDF) [file pcbi.1004334.s005.pdf]

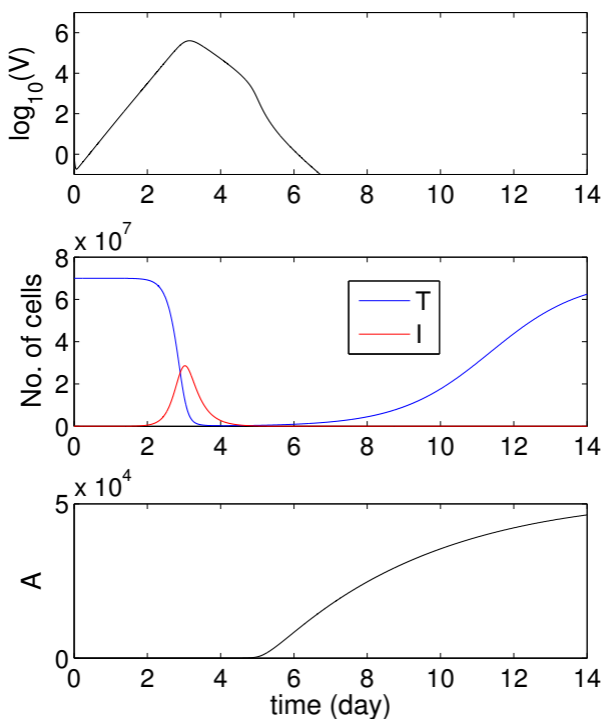

(A)

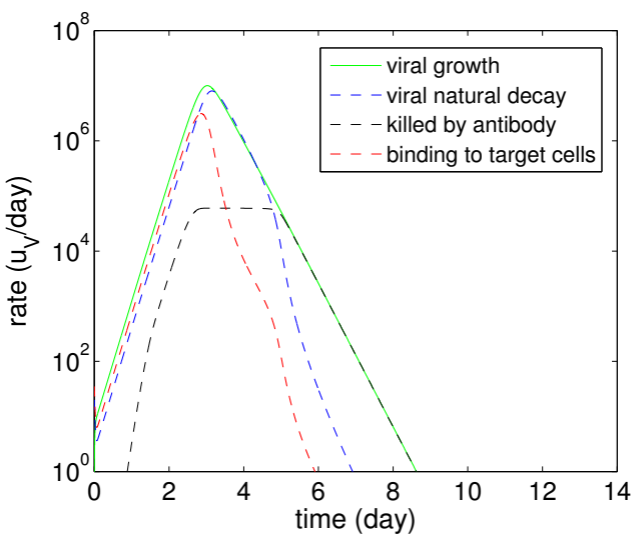

(B)

Supplement: S4 Fig — A solution of Eqs 1–7 in the main text for zero IFN production, q = 0 mimicking no time-dependent innate immunity, under the initial condition of V(0) = 1, T(0) = C t and zeros for all other variables. (A) shows time courses of important variables. (B) shows the time series of the four terms on the right-hand side of Eq 1, pI/(1+sF) (viral growth), cV (viral natural decay), μAV (killed by antibody), and βVT (binding to target cells), are calculated based on the solution and plotted in each panel. They represent the contribution of each term to the change of viral load (dV/dt). (PDF) [file pcbi.1004334.s006.pdf]

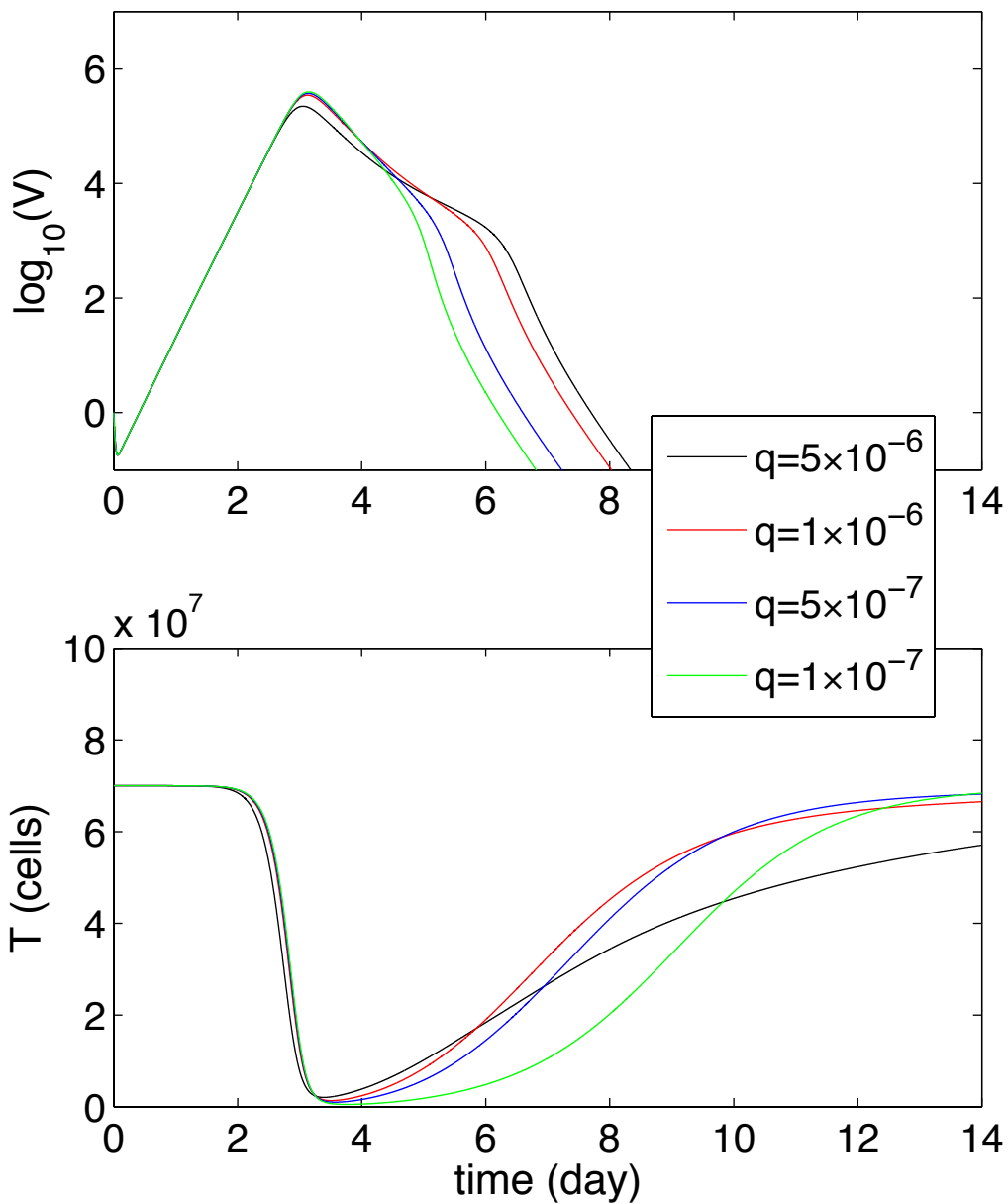

Supplement: S5 Fig — Time series show that a temporary depletion of the target cell pool occurs for four different IFN production rates (q). (PDF) [file pcbi.1004334.s007.pdf]

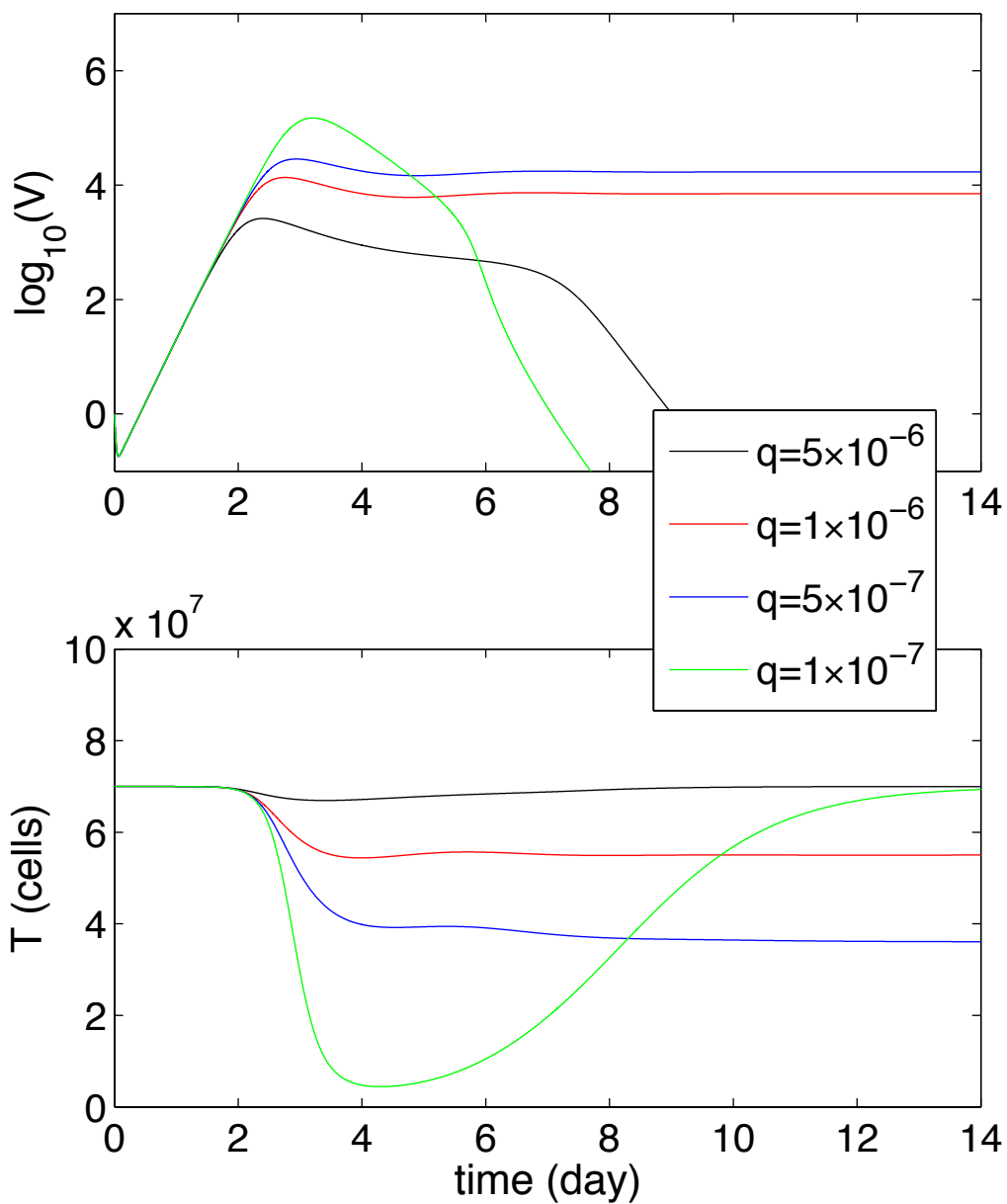

Supplement: S6 Fig — Time series show that a temporary depletion of the target cell pool occurs for a small IFN production rate (q = 10−7) but not for a large one (q = 5 × 10^−6^). However, for some intermediate values of q, the model loses the ability to clear virus, resulting in a sustained elevation of viral load. Possible reasons are explored in the Discussion. We use s = 1. (PDF) [file pcbi.1004334.s008.pdf]

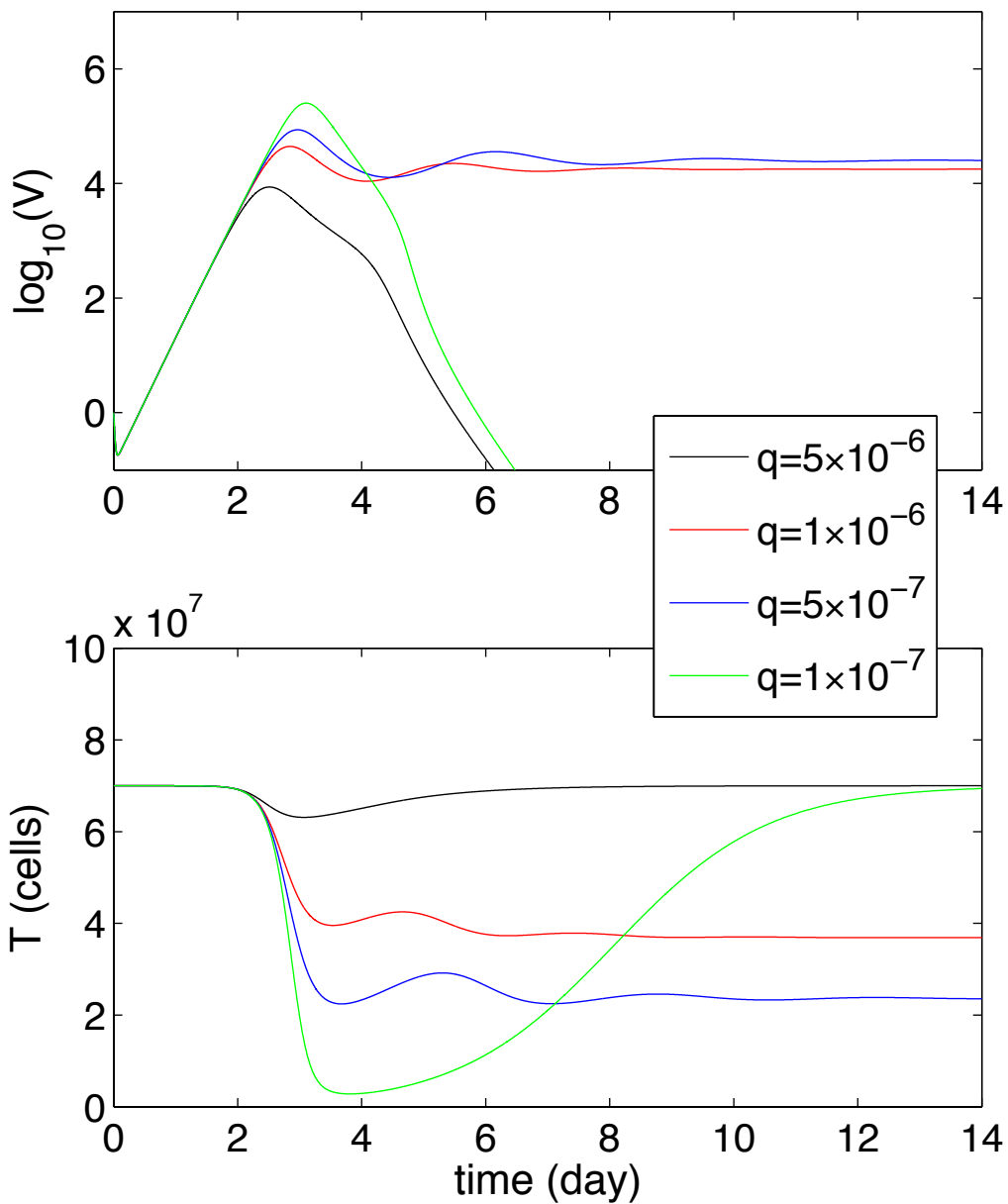

Supplement: S7 Fig — Time series show that a temporary depletion of the target cell pool occurs for a small IFN production rate (q = 10−7) but not for a large one (q = 5 × 10^−6^). However, for some intermediate values of q, the model loses the ability to clear virus, resulting in a sustained elevation of viral load. Possible reasons are explored in Discussion. We use κ = 3. (PDF) [file pcbi.1004334.s009.pdf]

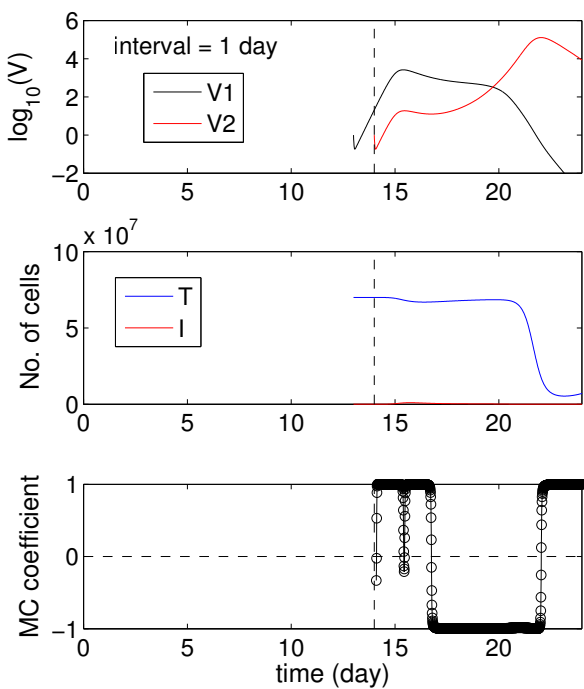

(A)

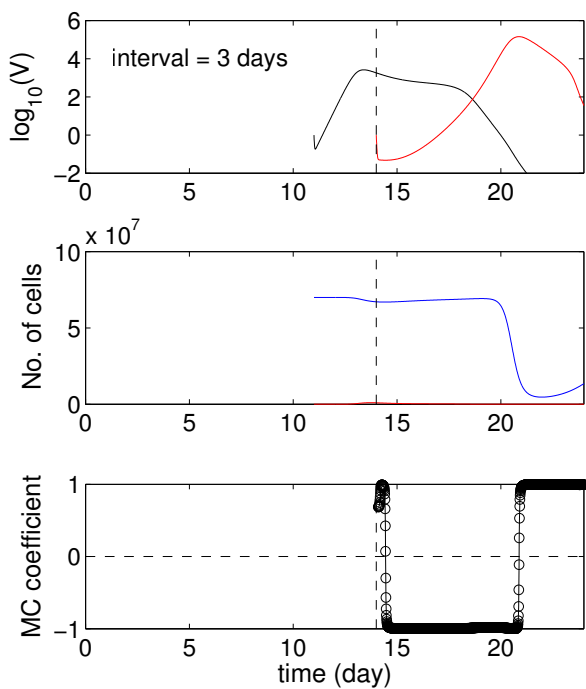

(B)

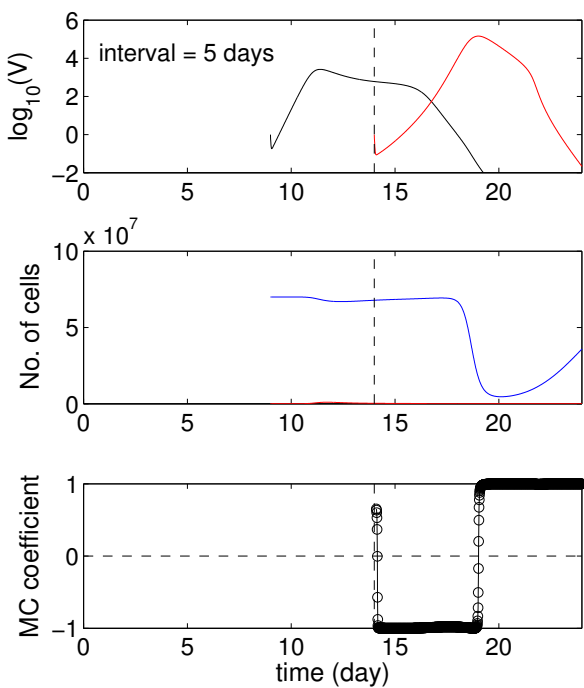

(C)

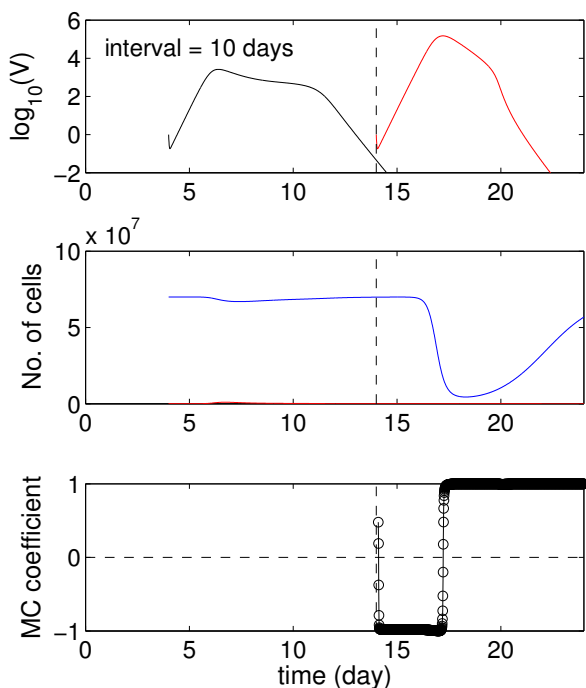

(D)

Supplement: S8 Fig — Simulations are done by using Model R2. Initial conditions are V 1 = 1, T = C t and zeros for all other variables at t = 0 day and V 2 = 1 is then introduced at t = 14 days indicated by dashed lines. We use s 1 = s 2 = 1, q 1 = 5 × 10^−6^ and q 2 = 10−7. The moving-correlation (MC) coefficient is used to indicate synchronisation/desynchronisation of the two viral loads. (PDF) [file pcbi.1004334.s010.pdf]

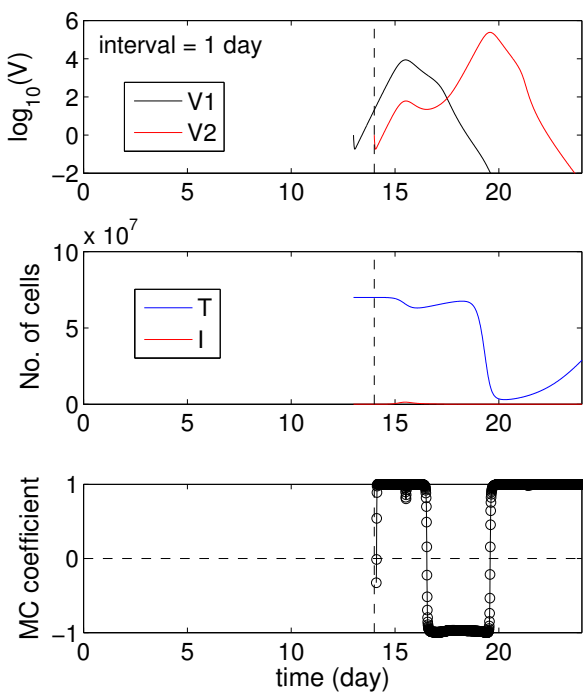

(A)

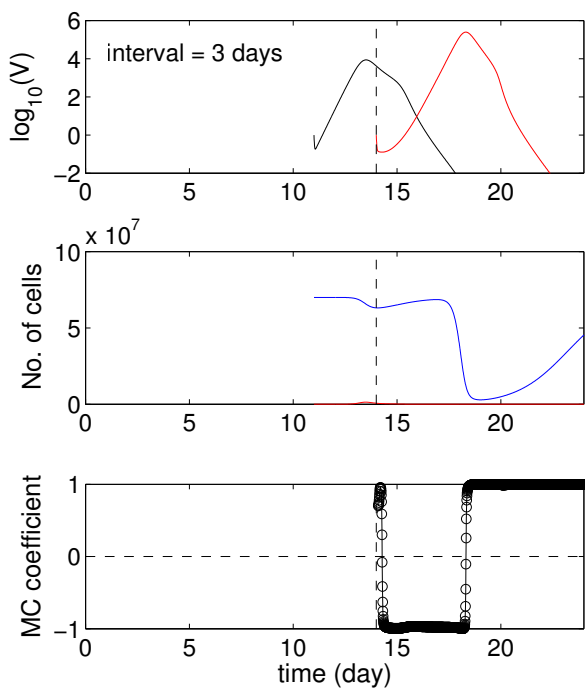

(B)

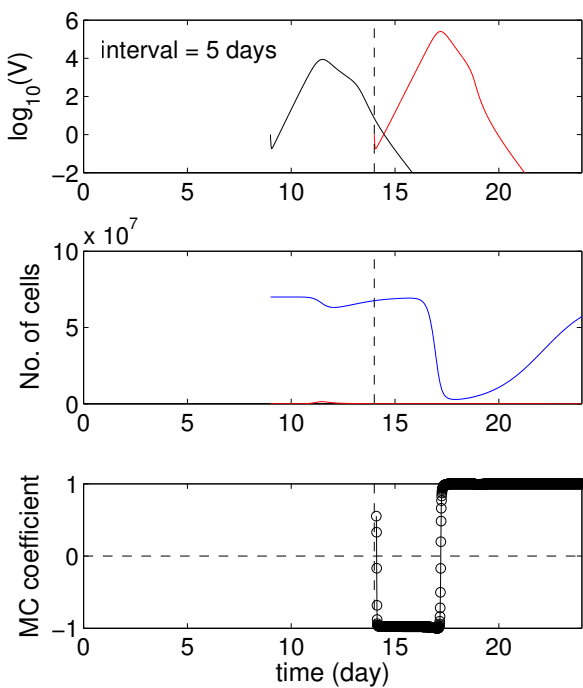

(C)

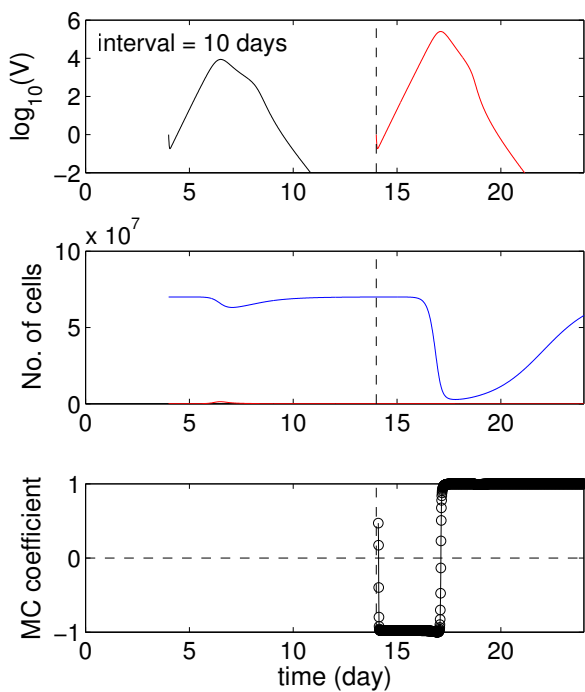

(D)

Supplement: S9 Fig — Simulations are done by using Model R3. Initial conditions are V 1 = 1, T = C t and zeros for all other variables at t = 0 day and V 2 = 1 is then introduced at t = 14 days indicated by dashed lines. We use κ 1 = κ 2 = 3, q 1 = 5 × 10^−6^ and q 2 = 10−7. The moving-correlation (MC) coefficient is used to indicate synchronisation/desynchronisation of the two viral loads. (PDF) [file pcbi.1004334.s011.pdf]

inter-exposure interval (day)

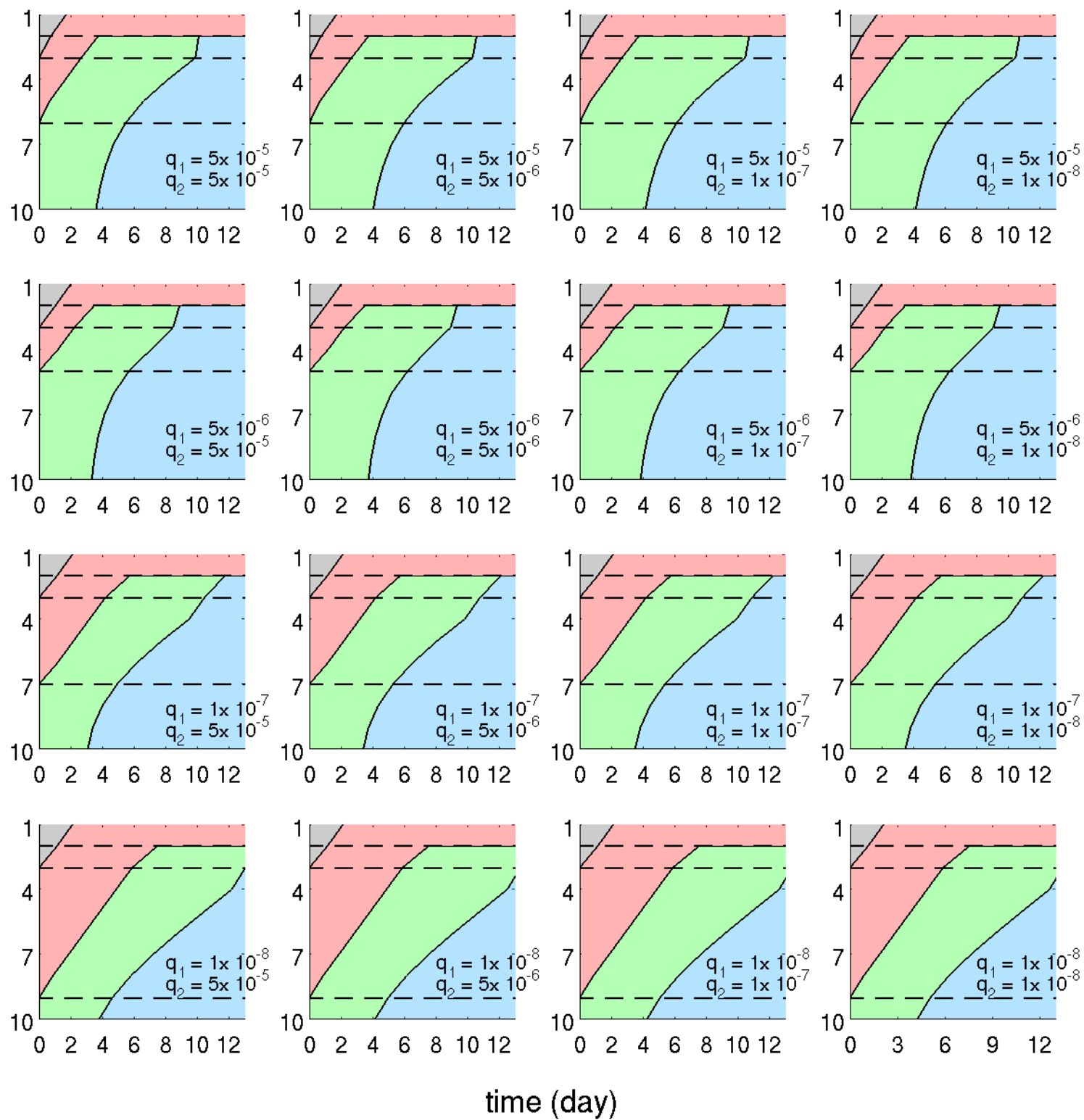

Supplement: S10 Fig — The value of the IFN production for the primary virus for Model R1 does not lead to any qualitative different patters of infection upon re-exposure. Both small and large values of q 1 result in target-cell depletion. The pattern is also independent of the choice of q 2. The model does not support the observed viral hierachy. The meaning of each colour is explained in Fig 8 in the main text. This figure is an extension of Fig 9 where only two intermediate q values are presented. (PDF) [file pcbi.1004334.s012.pdf]

inter-exposure interval (day)

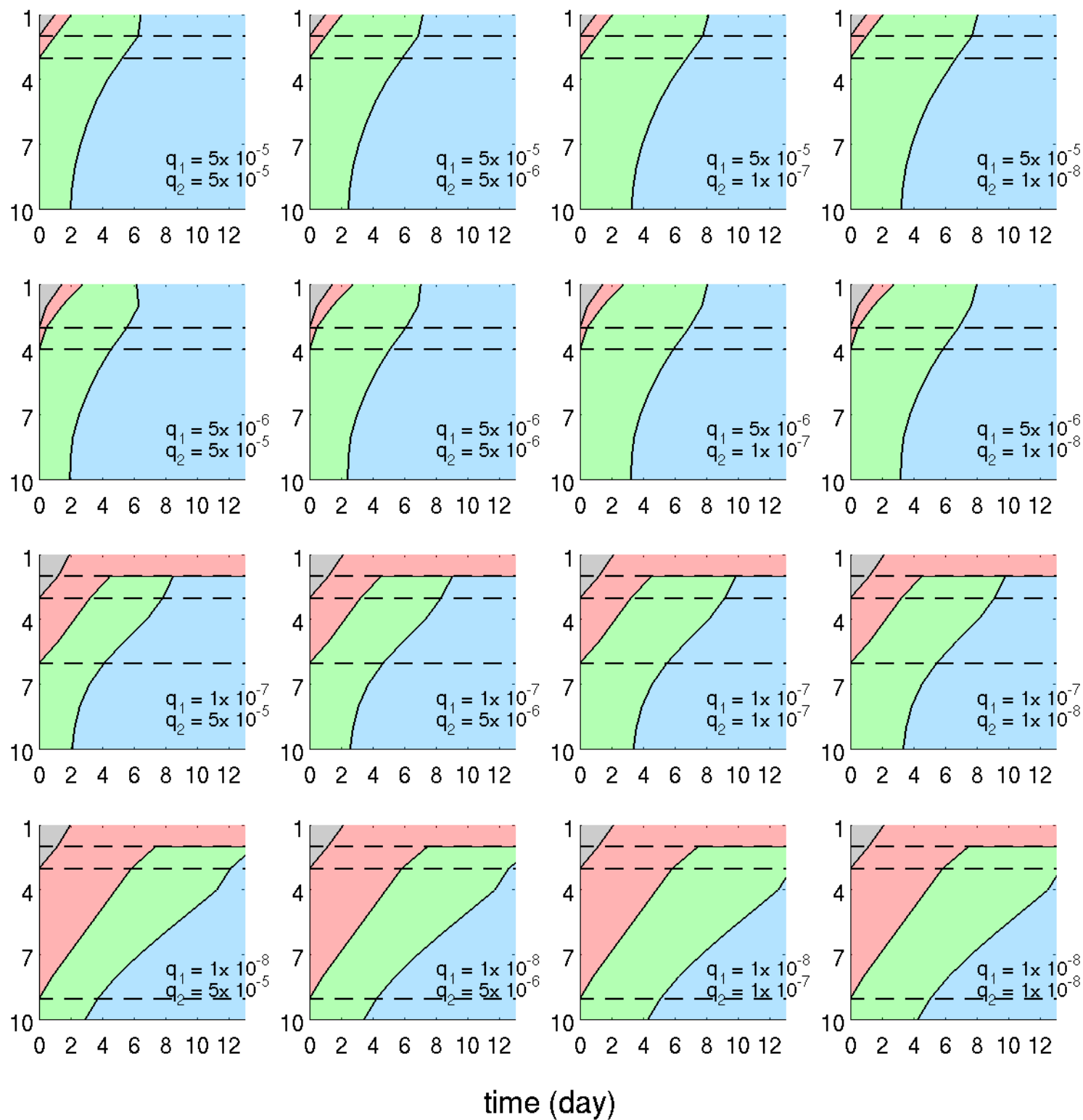

Supplement: S11 Fig — Different IFN production rates for the primary virus for Model R2 lead to qualitatively different patterns of infection upon re-exposure. The pattern is driven by q 1 and independent of the choice of q 2. The meaning of each colour is explained in Fig 8 in the main text. This figure is an extension of Fig 10 where only two intermediate q values are presented. We assume here that s 1 = s 2 = 1. (PDF) [file pcbi.1004334.s013.pdf]

inter-exposure interval (day)

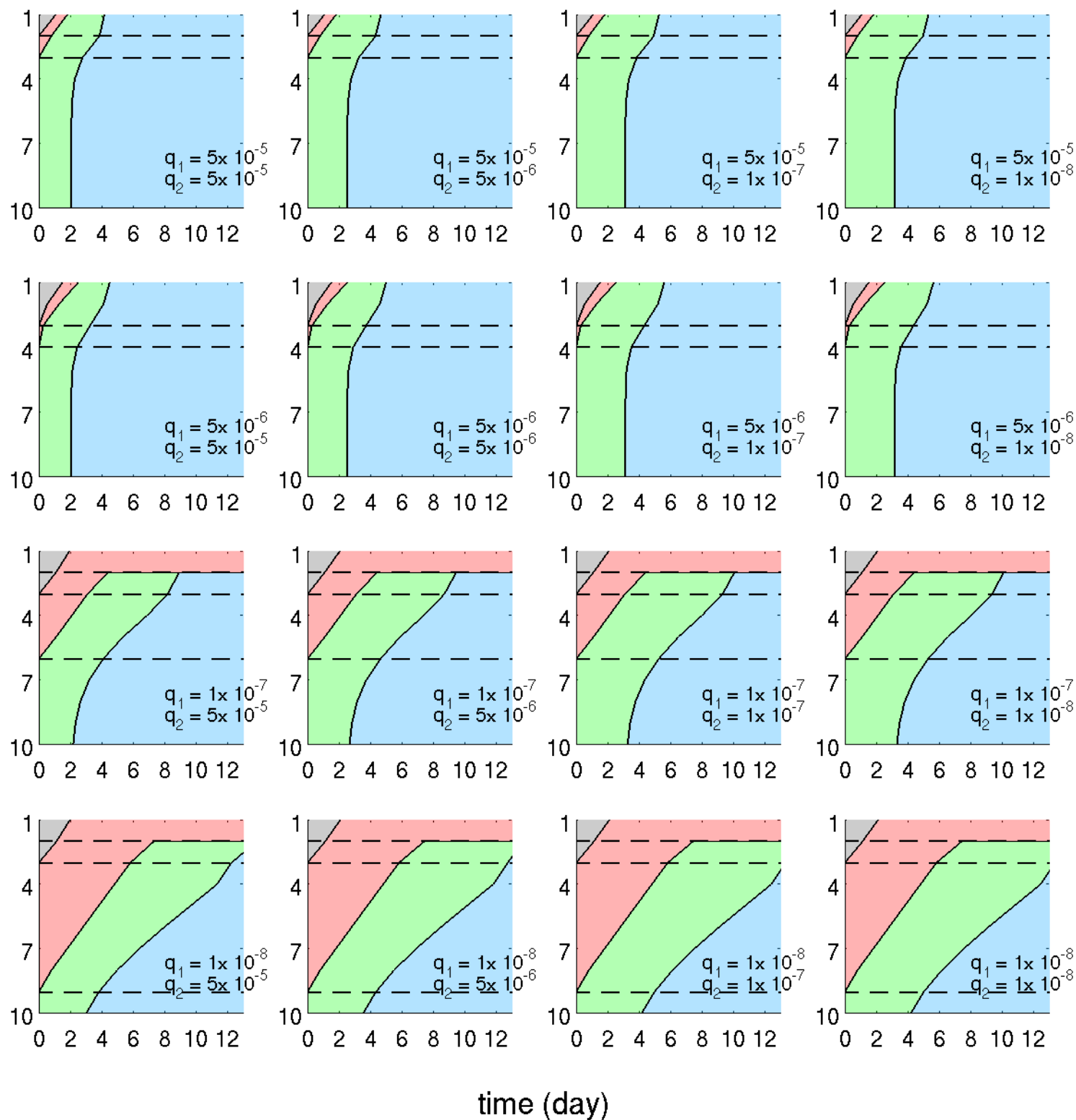

Supplement: S12 Fig — Different IFN production rates for the primary virus for Model R3 lead to qualitatively different patterns of infection upon re-exposure. The pattern is driven by q 1 and independent of the choice of q 2. The meaning of each colour is explained in Fig 8 in the main text. This figure is an extension of Fig 11 where only two intermediate q values are presented. We assume here that κ 1 = κ 2 = 3. (PDF) [file pcbi.1004334.s014.pdf]

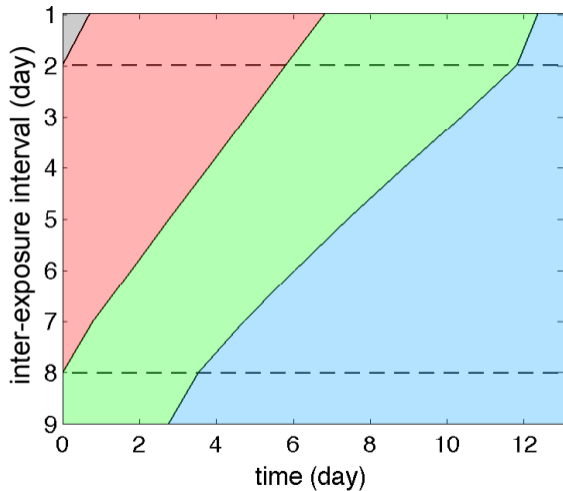

(A)  $s_1 = 1, s_2 = 10$

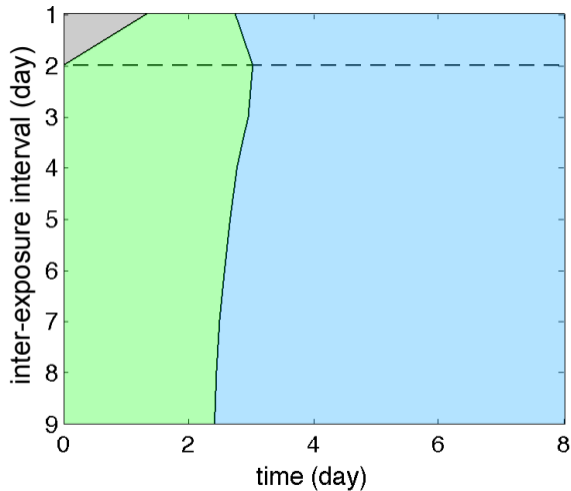

(B)  $s_1 = 10, s_2 = 1$

Supplement: S13 Fig — Varying the IFN-sensitivity parameter s in Model R2 leads to different patterns from that with the same IFN-sensitivity shown in Fig 9 in the main text. Note that q needs also to change accordingly for large s to maintain the change of viral load qualitatively similar to experimental observations. Thus, we choose q = 5 × 10^−6^ when s = 1 and q 2 = 5 × 10^−7^ when s = 10. Dashed lines separate different model behaviours in terms of the timing of the second virus challenge. (PDF) [file pcbi.1004334.s015.pdf]

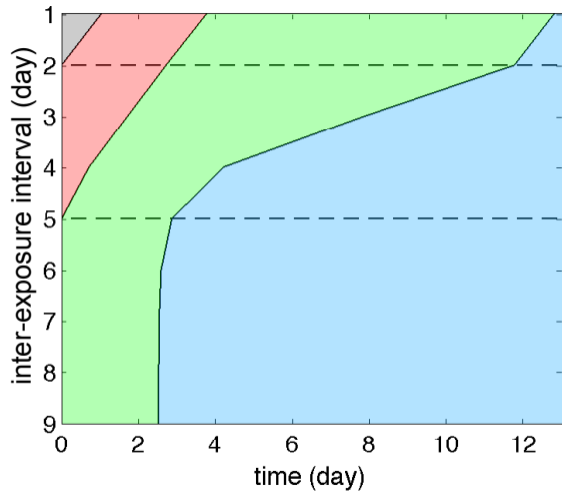

(A)  $\kappa_1 = 3, \kappa_2 = 15$

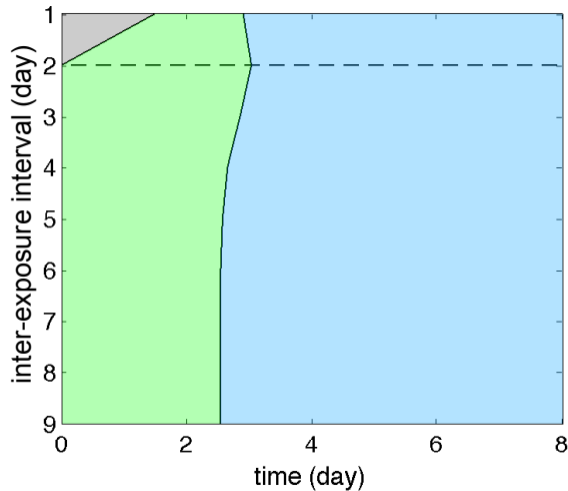

(B)  $\kappa_1 = 15, \kappa_2 = 3$

Supplement: S14 Fig — Varying the killing rate of virus by IFN-activated NK cells κ in Model R3 leads to different patterns from that with the same IFN-sensitivity shown in Fig 10 in the main text. Note again that q needs also to change accordingly for large κ to maintain the change of viral load qualitatively similar to experimental observations. Thus, we choose q = 5 × 10^−6^ when κ = 3 and q 2 = 1 × 10^−6^ when κ = 15. Dashed lines separate different model behaviours in terms of the timing of the second virus challenge. (PDF) [file pcbi.1004334.s016.pdf]
